# Apolipoprotein E Mimetic Peptide CN-105 and Postoperative Delirium in Older Patients: The Phase 2 MARBLE Randomized Clinical Trial

**DOI:** 10.1001/jamanetworkopen.2026.2289

**Published:** 2026-04-03

**Authors:** Noah J. Timko, Mary Cooter Wright, Melody R. Smith, Piper C. Boykin, Jacqueline M. Emerson, Marty G. Woldorff, Sarada Eleswarpu, J. Taylor Herbert, Eugene W. Moretti, Michael J. Devinney, Chakib M. Ayoub, Frank W. Rockhold, David Ryu, Ayesha Syed, Keith W. VanDusen, Thomas Bunning, Bethany J. Hsia, Michael P. Bolognesi, William A. Jiranek, Henrik Zetterberg, Kaj Blennow, Janet L. Huebner, Zhong Li, Tyler H. Reekes, Jeffrey N. Browndyke, Daniel T. Laskowitz, Miles Berger

**Affiliations:** 1 Department of Anesthesiology, Duke University School of Medicine, Durham, North Carolina; 2 Now with Department of Anesthesiology, Perioperative and Pain Medicine, Stanford University School of Medicine, Palo Alto, California; 3 Duke University School of Medicine, Durham, North Carolina; 4 Center for Cognitive Neuroscience, Duke University Medical Center, Durham, North Carolina; 5 Duke Institute for Brain Sciences, Duke University, Durham, North Carolina; 6 Department of Psychology and Neuroscience, Duke University, Durham, North Carolina; 7 Department of Neurobiology, Duke University School of Medicine, Durham, North Carolina; 8 Department of Psychiatry and Behavioral Sciences, Duke University Medical Center, Durham, North Carolina; 9 Duke-University of North Carolina (UNC) Alzheimer’s Disease Research Center, Duke University School of Medicine, Durham, North Carolina; 10 Department of Biostatistics and Bioinformatics, Duke University Medical Center, Durham, North Carolina; 11 Duke Clinical Research Institute, Duke University School of Medicine, Durham, North Carolina; 12 Now with University of North Carolina School of Medicine, Chapel Hill; 13 Department of Orthopaedic Surgery, Duke University School of Medicine, Durham, North Carolina; 14 Department of Psychiatry and Neurochemistry, Institute of Neuroscience and Physiology, Sahlgrenska Academy, University of Gothenburg, Mölndal, Sweden; 15 Department of Neurodegenerative Disease, University College London (UCL) Institute of Neurology, Queen Square, London, United Kingdom; 16 Hong Kong Center for Neurodegenerative Diseases, Clear Water Bay, Hong Kong, China; 17 Wisconsin Alzheimer’s Disease Research Center, University of Wisconsin School of Medicine and Public Health, University of Wisconsin-Madison, Madison; 18 United Kingdom (UK) Dementia Research Institute at UCL, London, United Kingdom; 19 Clinical Neurochemistry Laboratory, Sahlgrenska University Hospital, Mölndal, Sweden; 20 Paris Brain Institute, Institut du Cerveau (ICM), Pitié-Salpêtrière Hospital, Sorbonne University, Paris, France; 21 Neurodegenerative Disorder Research Center, Division of Life Sciences and Medicine, and Department of Neurology, Institute on Aging and Brain Disorders, University of Science and Technology of China (USTC) and First Affiliated Hospital of USTC, Hefei, China; 22 Duke Molecular Physiology Institute, Duke University, Durham, North Carolina; 23 Duke University Proteomics and Metabolomics Core Facility, Durham, North Carolina; 24 Durham Veterans Affairs Medical Center, Durham, North Carolina; 25 Department of Neurology, Duke University School of Medicine, Durham, North Carolina; 26 Department of Neurosurgery, Duke University School of Medicine, Durham, North Carolina; 27 Center for the Study of Aging and Human Development, Duke University, Durham, North Carolina

## Abstract

**Importance:**

The apolipoprotein E (*APOE*) gene ε4 allele leads to increased Alzheimer disease risk and neuroinflammation and is also believed to play a role in postoperative delirium. However, the safety and feasibility of modulating apoE protein signaling to reduce postoperative neuroinflammation and delirium in older adults are unclear.

**Objective:**

To assess the safety and feasibility of the apoE mimetic peptide CN-105 for reducing delirium incidence and severity and neuroinflammation after noncardiac or nonintracranial surgery in older adults.

**Design, Setting, and Participants:**

This triple-blind, escalating dose, phase 2 randomized clinical trial enrolled patients from April 17, 2019, to December 28, 2022, at a tertiary academic medical center. Included patients were 60 years or older and scheduled for a noncardiac or nonintracranial surgery. Exclusion criteria were incarceration, planned chemotherapy within 6 weeks after surgery, or inability to undergo lumbar punctures. Data analyses were based on a modified intention-to-treat approach and were performed from August 14, 2023, to August 22, 2025.

**Interventions:**

Patients were randomly assigned 3:1 to the CN-105 group or placebo group. The CN-105 group received intravenous CN-105 doses of 0.1, 0.5, or 1 mg/kg starting within 1 hour before surgery and administered every 6 hours afterward until hospital discharge or 13 doses were received. Patients in the placebo group followed the same administration schedule.

**Main Outcomes and Measures:**

The primary outcome was safety—the incidence and number of postoperative adverse events (AEs). Secondary outcomes included feasibility (rate of drug doses administered within 90 minutes of schedule), postoperative delirium incidence and severity, and postoperative changes in cerebrospinal fluid (CSF) cytokine levels (interleukin [IL] 6, granulocyte-colony stimulating factor [G-CSF], monocyte chemoattractant protein-1 [MCP-1], and IL-8).

**Results:**

Among 203 enrolled patients, 186 (mean [SD] age, 68.7 [5.2] years; 119 males [64.0%]) were randomized (137 to the CN-105 group, 49 to the placebo group) and underwent surgery. The rates of grade 2 or higher AEs among patients in the CN-105 and placebo groups were 76.6% and 87.8% (relative risk [RR], 0.87; 95% CI, 0.76-1.00; *P* = .10). The CN-105 vs placebo group had fewer grade 2 or higher AEs per patient (median [IQR], 1 [1-3] vs 2 [1-5]; *P* = .03). The percentage of CN-105 doses administered within the time window was 94.6% (860 of 909; 95% CI, 92.9%-96.0%) in the CN-105 group and 93.8% (346 of 369; 95% CI, 90.8%-96.0%) in the placebo group. Among patients in the CN-105 vs placebo group, the postoperative delirium incidence was 19.3% vs 26.5% (odds ratio [OR], 0.66; 95% CI, 0.31-1.42; *P* = .29); the median (IQR) postoperative delirium severity scores were 1 (1-2) vs 2 (1-2) (*P* = .19); and the median difference in preoperative to 24-hour postoperative CSF cytokine-level changes were as follows: −0.39 pg/mL (95% CI, −0.93 to 0.14 pg/mL, *P* = .12) for IL-6, −0.84 pg/mL (95% CI, −3.06 to 1.40 pg/mL; *P* = .18) for G-CSF,−23.32 pg/mL (95% CI, −94.36 to 44.93 pg/mL; *P* = .57) for IL-8, and −2.36 pg/mL (95% CI, −58.57 to 58.62 pg/mL; *P* = .50) for MCP-1.

**Conclusions and Relevance:**

In this phase 2 randomized clinical trial of older surgical patients, CN-105 (vs placebo) administration was feasible and did not increase AEs. A phase 3 trial is warranted to further evaluate the efficacy of CN-105 for reducing postoperative AEs and to more precisely determine its effects on postoperative delirium incidence and severity.

**Trial Registration:**

ClinicalTrials.gov Identifier: NCT03802396

## Key Points

**Question:**

Is the apolipoprotein E mimetic peptide CN-105 safe and feasible for reducing postoperative delirium in older surgical patients?

**Findings:**

In this phase 2 randomized clinical trial involving 186 patients, those who received intravenous CN-105 (vs placebo) experienced fewer grade 2 or higher postoperative adverse events, and more than 90% of CN-105 doses were administered within the appropriate time window. The postoperative delirium rates in the CN-105 and placebo groups were 19.3% vs 26.5%, respectively.

**Meaning:**

The findings suggest that CN-105 was feasible to administer and did not lead to any meaningful safety issues; a phase 3 trial is warranted to study the efficacy of CN-105 for reducing postoperative adverse events and to more precisely determine its effects on postoperative delirium.

## Teaser

This randomized clinical trial investigates the safety and feasibility of the apolipoprotein E mimetic peptide CN-105 vs placebo for reducing postoperative delirium in older patients.

## Introduction

Delirium is the most common postoperative complication in older adults, characterized by fluctuations in attention and consciousness, an increased risk for Alzheimer disease (AD), related dementias, longer hospital stays, mortality, and high health care costs (approximately $82.4 billion per year in the US).^1^ Yet, there are no US Food and Drug Administration (FDA)–approved drugs to treat or prevent delirium, in part due to the inadequate understanding of its pathophysiological processes.

Evidence suggests that neuroinflammation plays a core role in delirium,^2,3^ which has led to the idea that delirium can be viewed as an acute model of inflammation-evoked cognitive impairments that occur over longer periods in AD. One risk factor for both AD and delirium is the apolipoprotein E *(APOE)* gene ε4 allele, which is itself associated with neuroinflammation.^4^ Thus, decreasing neuroinflammation by modulating apoE protein signaling may reduce delirium. However, the apoE protein does not cross the blood-brain barrier, which precludes peripheral administration of the protective apoE2 protein variant.^5^ To circumvent these limitations, a small pentapeptide (CN-105) was developed from the apoE receptor–binding domain that does cross the blood-brain barrier.^6,7^

Studies have demonstrated that apoE and apoE mimetic peptides, such as CN-105, bind to low-density lipoprotein receptor–related protein-1 (LRP1) on microglia,^8^ which inhibits microglial activation and inflammatory mediator release.^9,10^ CN-105 was designed to mimic the hydrophilic receptor binding face of the amphipathic apoE receptor binding region that interacts with LRP1, to block microglial activation and neuroinflammation.^11,12^ CN-105 blocked neuroinflammation and improved functional outcomes in multiple murine models of brain injury^11,12,13,14,15,16^ (regardless of *APOE* genetic background), which raises the possibility that CN-105 could reduce delirium incidence by blocking neuroinflammation. Two phase 1 studies have demonstrated the safety, linear pharmacokinetic profile, and tolerability of CN-105,^17,18^ and CN-105 was safely administered to patients with intracerebral hemorrhage in a phase 2 study.^19^ Thus, we conducted the phase 2 MARBLE (Modulating ApoE Signaling to Reduce Brain Inflammation, Delirium, and Postoperative Cognitive Dysfunction) trial to assess the safety and feasibility of CN-105 for reducing delirium incidence and severity and neuroinflammation after noncardiac or nonintracranial surgery in older adults.

## Methods

### Trial Design and Setting

MARBLE was a single-center, escalating dose, triple-blind, placebo-controlled phase 2 randomized clinical trial conducted at the tertiary academic medical center Duke University Medical Center in Durham, North Carolina. Since Duke University owns the intellectual property on CN-105, the Duke Office of Scientific Integrity–Conflict of Interest Office reviewed the study and required external Institutional Review Board regulatory oversight. Thus, the Western Institutional Review Board approved this study prior to patient recruitment. The trial protocol^20^ and statistical analysis plan are available in Supplement 1. Participants provided written informed consent before enrollment. We followed the Consolidated Standards of Reporting Trials (CONSORT) reporting guideline.^14^

### Participants and Randomization

Enrollment occurred between April 17, 2019, and December 28, 2022. Eligible individuals spoke English, were aged 60 years or older, and were scheduled for a 2-hour or more elective major noncardiac or nonintracranial surgery with overnight hospitalization. Exclusion criteria were incarceration, planned systemic chemotherapy before the 6-week postoperative study visit, and inability to undergo lumbar punctures.^21^ There were no preoperative cognitive status exclusions. Race and ethnicity were self-identified by patients and then confirmed during prospective review of their EPIC electronic medical record (EMR) classifications (Epic Systems Corp). Race and ethnicity data (race: American Indian or Alaska Native, Asian, Black or African American, Native Hawaiian or Other Pacific Islander, White, more than 1 race, unknown or not reported; ethnicity: Hispanic or Latino, not Hispanic or Latino, and unknown or not reported) were collected according to reporting guidelines of the FDA and ClinicalTrials.gov.

The MARBLE trial enrolled 3 successive randomized cohorts of 67 patients, of whom 50 were assigned to CN-105 and 17 were assigned to placebo. To ensure the safety of each CN-105 dose before moving to a higher dose: cohort 1 received placebo or 0.1 mg/kg CN-105, cohort 2 received placebo or 0.5 mg/kg CN-105, and cohort 3 received placebo or 1 mg/kg CN-105; these doses were chosen based on phase 1 trial safety data.^17,18^ The trial was powered to compare outcomes between combined CN-105 dose groups and placebo groups; it was not designed or powered to investigate dose-specific effects.

**Figure 1.**
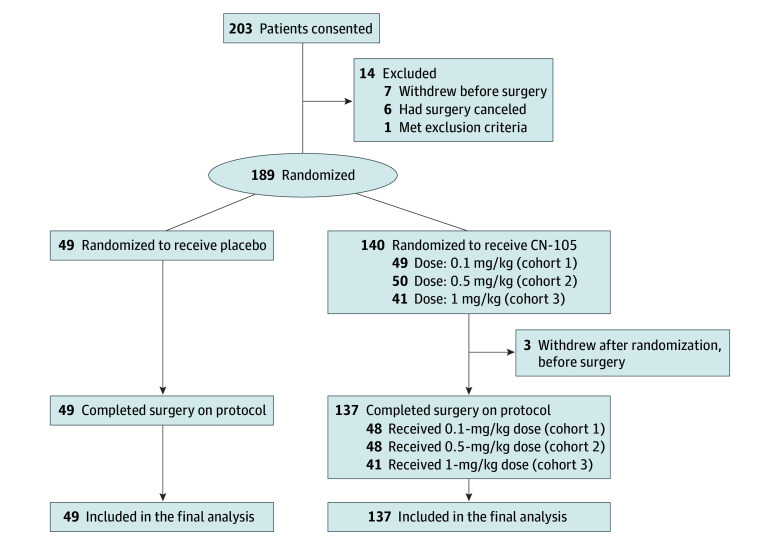
Trial Flow Diagram

The MARBLE trial used a permuted block (block size of 8, plus 1 randomly placed block size of 3) randomization list. Due to human error, 2 additional patients were enrolled in cohort 1 (n = 69), which increased total enrollment to 203 (Figure 1). Data analyses used a modified intention-to-treat approach among randomized patients who completed surgery on protocol (Statistical Analysis Plan in Supplement 1).

### CN-105 or Placebo Preparation, Masking, and Administration 

The Duke University Hospital Investigational Drug Service provided CN-105 or placebo in plastic intravenous (IV) bags with opaque shrouds that appeared identical whether they contained CN-105 or placebo (eMethods in Supplement 2). Patients received IV CN-105 or placebo, administered by clinical bedside nurses, within 1 hour before surgery and every 6 hours (within 90 minutes of schedule) after surgery start for up to 3 days (13 doses) or hospital discharge, whichever occurred first. Other than CN-105 or placebo administration, there were no restrictions on intraoperative or postoperative drug administration, which was performed per standard clinical care by the clinicians caring for these patients. Clinicians determined when patients were ready for hospital discharge, without input from the study team.

### Safety Assessment

The a priori–specified primary outcome was safety—that is, the incidence and number of postoperative adverse events (AEs) per patient in the CN-105 and placebo groups until the 6-week study follow-up visit.^20^ We assessed grade 2 or higher AEs according to the Common Terminology Criteria for Adverse Events, version 5.0 (CTCAE v5.0)^22^ because grade 2 or higher AEs reflect events that warrant clinical intervention or changes in postoperative patient management (whereas grade 1 AEs typically do not warrant any intervention and would not be considered AEs in the postoperative setting by surgeons or anesthesiologists). Secondary analyses included severe AEs (SAEs) and grade 2 or higher AEs by CTCAE v5.0–classified body systems. AEs were monitored via participant reports and by blinded study physician review of the EMR, from first CN-105 or placebo administration until the 6-week postoperative study visit. An independent data safety monitoring board consisting of external university faculty evaluated unblinded patient safety data after completion of cohorts 1 and 2 (and could have recommended study termination based on specific criteria) (eMethods in Supplement 2).^20^

### Blood and Cerebrospinal Fluid Sampling and Biomarkers

Blood and cerebrospinal fluid (CSF) samples were collected within 2 months before surgery and 24 hours and 6 weeks after surgery.^20^ Blood was used for CN-105 measurements and *APOE* genotyping.^23^ CSF was collected for CN-105 measurements, AD biomarker, and cytokine assays (eMethods in Supplement 2).

### CN-105 Administration Feasibility and Measurements

Feasibility of perioperative CN-105 administration was measured by the rate of doses given within the protocol-specified time window during hospitalization.^20^ CN-105 levels in plasma and CSF were measured using ultraperformance liquid chromatography-tandem mass spectrometry (eMethods in Supplement 2).

### Delirium and Cognitive Assessments

Delirium was assessed using the 3-Minute Diagnostic Confusion Assessment Method (3D-CAM) in nonintubated patients^24^ and the CAM for the Intensive Care Unit in intubated or nonverbal patients^25^ given the greater sensitivity of each of these assessments in these respective groups.^26^ Delirium assessments were performed at the baseline preoperative visit and twice daily in the morning and evening on postoperative days 1 to 5 or until hospital discharge, whichever occurred first. At the start of cohort 2 and onward, based on new clinical guidelines,^27^ delirium was also assessed in the postanesthesia care unit (using 3D-CAM) and in the evening following surgery. Patients were defined as having delirium if any postoperative delirium assessment indicated the presence of delirium, per the established criteria for a positive assessment for each of the delirium screening tools.^24,25^ Delirium severity was assessed via the 20-point 3D-CAM severity scale^28^ (score range: 0-20, with higher scores indicating greater severity) using the peak value observed across all completed 3D-CAM assessments conducted up to and on postoperative day 5. Supplement 2 provides information on delirium assessor training.

Patients also underwent a cognitive test battery^20,29,30^ before surgery and 6 weeks after surgery.^20^ Scores from individual battery tests were standardized (*z* score transformation) and combined into 4 cognitive domains, which were averaged to provide the Continuous Cognitive Index (CCI), a summary global measure of cognition. The range of CCI scores obtained in our cohort was –1.56 (from baseline assessment) to 1.17 (from 6-week assessment). A score of 0 indicates average, negative scores indicate below-average, and positive scores indicate above-average cognitive performance. The eMethods in Supplement 2 provides details on CCI.

### Statistical Analysis

For the primary end point of safety (incidence and number of postoperative AEs), we anticipated an AE incidence of 5% among patients in the placebo group based on AE rates in older (aged ≥60 years) noncardiac surgical patients (according to email communication between Miles Berger, MD, PhD, and Grant Garrigues, MD, November 30, 2016). Thus, 156 patients (117 in the CN-105 group, 39 in the placebo group) would provide greater than 80% power (with α = .05) in a 2-sample, 2-sided unpooled variance χ^2^ test to detect an absolute difference of 14.8% or higher AE rates between CN-105 and placebo groups. We assumed that approximately 80% of enrolled patients would complete the study, and we planned to enroll 201 patients; thus, at completion there would be at least 39 patients randomized to placebo and at least 117 patients randomized to CN-105 (≥39 patients per dose level). If completion rates were greater than 80%, we planned to evaluate whether enrollment could end early.

No imputation was performed for missing data. A χ^2^ test and a Wilcoxon rank sum test were used to evaluate AE incidence and number of AEs per patient, respectively, between the 2 groups. Feasibility was evaluated via binomial 95% CIs for within-window dose administration rate, with an a priori threshold of 90% or greater for feasible drug administration.^20^

For the other secondary end points beyond feasibility (delirium incidence and severity, CSF cytokine levels, and cognitive outcomes), we performed unpaired, 2-tailed *t* tests, Wilcoxon rank sum tests, or χ^2^ tests, as appropriate, to compare group outcomes. The 95% CIs for median CSF cytokine differences were generated via bootstrapping. Post hoc multivariable regression was used to compare AE and delirium incidence (Firth-corrected binary logistic), number of AEs per patient (negative binomial regression), CCI (linear regression), and delirium severity (ordinal logistic) outcomes. All multivariable models were adjusted for age, sex, race, *APOE* ε4 carrier status, Elixhauser-van Walraven Comorbidity Index^31^ (range: –19 to 89, with higher scores indicating greater in-hospital mortality risk), surgery duration, and intraoperative blood loss. To investigate whether CN-105’s effect differed by *APOE* ε4 carrier status, we added a drug by *APOE* ε4 interaction effect to the multivariable models. All hypothesis testing was 2-sided, with a statistical significance threshold set at .05.

Each MARBLE trial participant was assigned a unique study identification number. Data were securely stored in REDCap (Research Electronic Data Capture; Vanderbilt University).^32^ Data analyses were performed from August 14, 2023, to August 22, 2025, using SAS version 9.4 (SAS Institute) and R version 4.2 or higher (R Project for Statistical Computing).

## Results

### Patient Characteristics

Study enrollment is depicted in Figure 1. Due to higher than anticipated completion in cohorts 1 and 2, 55 (instead of 67) patients were enrolled in cohort 3. Among 186 participants (137 in the CN-105 group, 49 in the placebo group) who completed surgery on protocol, the mean (SD) age was 68.7 (5.2) years, 119 (64.0%) were male and 67 (36.0%) were female, and 58 (31.5%) were *APOE* ε4 carriers (additional details on *APOE* genotype distributions are provided in eTable 1 in Supplement 2). No patients were intubated at study enrollment. Moreover, none of the surgical procedures involved operating on the central nervous system. Baseline and perioperative patient characteristics are listed in the Table, showing that the CN-105 and placebo groups were generally well randomized. Additional preoperative patient characteristics are shown in eTable 1 in Supplement 2.

**Table.** Baseline and Perioperative Patient Characteristics by Randomization Group (Modified Intention to Treat)

Patient characteristic	Patients, No. (%)		
	Placebo group (n = 49)	CN-105 group (n = 137)	Total (n = 186)
**Baseline**			
Age, mean (SD), y	69.5 (5.7)	68.4 (5.1)	68.7 (5.2)
Sex			
Female	16 (32.7)	51 (37.2)	67 (36.0)
Male	33 (67.3)	86 (62.8)	119 (64.0)
Weight, mean (SD), kg	90.8 (19.4)	87.5 (17.8)	88.4 (18.2)
Height, mean (SD), cm	173.7 (12.4)	171.4 (10.0)	172.1 (10.7)
BMI, mean (SD)	29.8 (4.0)	29.7 (5.2)	29.7 (4.9)
Race^a^			
Black or African American	5 (10.2)	9 (6.6)	14 (7.5)
White	42 (85.7)	122 (89.1)	164 (88.2)
Other^a^	2 (4.1)	6 (4.4)	8 (4.3)
Ethnicity^a^			
Hispanic or Latino	1 (2.0)	2 (1.5)	3 (1.6)
Not Hispanic or Latino	47 (95.9)	135 (98.5)	182 (97.8)
Unknown or not reported	1 (2.0)	0 (0.0)	1 (0.5)
No. of y of education, median (IQR)^b^	16 (14-18)	16 (14-18)	16 (14-18)
*APOE* ε4 carrier status^b^^,^^c^	14 (29.2)	44 (32.4)	58 (31.5)
Family history of cognitive disease^b^	27 (55.1)	79 (57.7)	106 (57.0)
Mild cognitive impairment^d^	1 (2.0)	1 (0.7)	2 (1.1)
Alzheimer disease^d^	0	0	0
Diabetes^d^	10 (20.4)	28 (20.4)	38 (20.4)
Hypertension^d^	27 (55.1)	83 (60.6)	110 (59.1)
High cholesterol^d^	20 (40.8)	59 (43.1)	79 (42.5)
Elixhauser-van Walraven Comorbidity Index, mean (SD)^d^^,^^e^	1.7 (3.2)	2.5 (5.2)	2.3 (4.7)
MoCA score, mean (SD)^b^^,^^f^	24.2 (3.5)	24.6 (3.2)	24.5 (3.3)
CCI (global cognition), mean (SD)^b^^,^^g^	0.01 (0.54)	0.01 (0.55)	0.01 (0.55)
Attention domain score, mean (SD)^b^^,^^g^	−0.03 (0.97)	0.01 (0.82)	0.00 (0.86)
Verbal memory domain score, mean (SD)^b^^,^^g^	0.03 (0.73)	0.02 (0.88)	0.02 (0.84)
Visual memory domain score, mean (SD)^b^^,^^g^	0.02 (0.77)	0.02 (0.87)	0.02 (0.85)
Executive functioning or processing speed domain score, mean (SD)^b^^,^^g^	0.04 (0.55)	0.00 (0.52)	0.01 (0.52)
**Perioperative**			
Surgical service			
Thoracic	3 (6.1)	8 (5.8)	11 (5.9)
General	6 (12.2)	24 (17.5)	30 (16.1)
Gynecologic	4 (8.2)	3 (2.2)	7 (3.8)
Orthopedic	18 (36.7)	59 (43.1)	77 (41.4)
Otolaryngologic	3 (6.1)	6 (4.4)	9 (4.8)
Plastic	1 (2.0)	7 (5.1)	8 (4.3)
Urologic	14 (28.6)	30 (21.9)	44 (23.7)
Surgery duration, median (IQR), h	2.9 (2.1-3.9)	2.2 (1.6-3.4)	2.4 (1.6-3.7)
Estimated blood loss, median (IQR), mL	125 (30-200)	100 (10-200)	100 (10-200)
Spinal anesthetic placement	15 (30.6)	42 (30.7)	57 (30.6)
Peripheral nerve block	17 (34.7)	58 (42.3)	75 (40.3)
Epidural placement	3 (6.1)	11 (8.0)	14 (7.5)

Abbreviations: *APOE* ε4, apolipoprotein E gene ε4 allele; BMI, body mass index (calculated as weight in kilograms divided by height in meters squared); CCI, Continuous Cognitive Index; MoCA, Montreal Cognitive Assessment.

^a^ Self-reported by patients and then confirmed during prospective review of electronic medical record classifications. Other category included unknown or not reported (n = 2) in the placebo group and American Indian or Alaska Native (n = 2), Asian (n = 3), and unknown or not reported (n = 1) in the CN-105 group.

^b^ Characteristic obtained prospectively by research staff.

^c^ *APOE* ε4 genotype could not be obtained for 2 patients. eTable 1 in Supplement 2 provides detailed *APOE* genotypes.

^d^ Diagnosis obtained from the electronic medical record.

^e^ Elixhauser-van Walraven Comorbidity Index, modified from the Elixhauser Comorbidity Index, is predictive of hospital mortality, with a range of –19 to 89 (higher scores indicating greater in-hospital mortality risk).

^f^ MoCA score range: 0 to 30, with higher scores indicating better cognitive performance.

^g^ Included enrolled patients who completed baseline cognitive testing (n = 194). Scores from individual battery tests were standardized (z score transformation) and combined into 4 cognitive domains, which were averaged to provide the Continuous Cognitive Index (CCI), a summary global measure of cognition. The range of CCI scores obtained in our cohort was –1.56 (from baseline assessment) to 1.17 (from 6-week assessment). A score of 0 indicates average, negative scores indicate below-average, and positive scores indicate above-average cognitive performance. For additional information, see the eMethods in Supplement 2.

### CN-105 Level Measurements

Patients received a median (IQR) of 5 (5-9) doses in the CN-105 group and 6 (5-11) in the placebo group (*P* = .17). In 24-hour postoperative plasma and CSF samples, CN-105 was detectable in proportionately increasing levels in the 3 dose groups, with lower levels detected in CSF vs plasma (cohort 1, 0.1-mg/kg dose: 4.8 [2.7-7.9] ng/mL vs 151.8 [74.2-209.2] ng/mL; cohort 2, 0.5-mg/kg dose: 29.1 [25.0-52.7] ng/mL vs 685.4 [455.9-1303.6] ng/mL; cohort 3, 1-mg/kg dose: 60.2 [48.9-91.4] ng/mL vs 1448.6 [905.1-2791.0] ng/mL) (eFigure in Supplement 2).

### Safety

Grade 2 or higher AE incidence was 76.6% (105 of 137) in the CN-105 group vs 87.8% (43 of 49) in the placebo group (relative risk [RR], 0.87; 95% CI, 0.76-1.00; *P* = .10); the results were similar in a multivariable model (eTable 2 in Supplement 2). The median (IQR) number of grade 2 or higher AEs per patient was lower in the CN-105 group than the placebo group (1 [1-3] vs 2 [1-5]; *P* = .03). These results remained consistent when controlling for covariates (incidence rate ratio for number of grade ≥2 AEs per patient, 0.65; 95% CI, 0.47-0.91; *P* = .01) (eTable 3 in Supplement 2). Furthermore, when an interaction term for the effect of *APOE* ε4 carrier status by randomization group was added to this multivariable model, it had no significant effect on number of grade 2 or higher AEs per patient.

SAE incidence was lower in the CN-105 group than the placebo group (8.0% [11 of 137] vs 22.4% [11 of 49]; χ^2^ *P* = .007). The median (IQR) number of SAEs per patient was also lower in the CN-105 vs placebo group (0 [0-0] vs 0 [0-0]; Wilcoxon *P* = .007).

**Figure 2.**
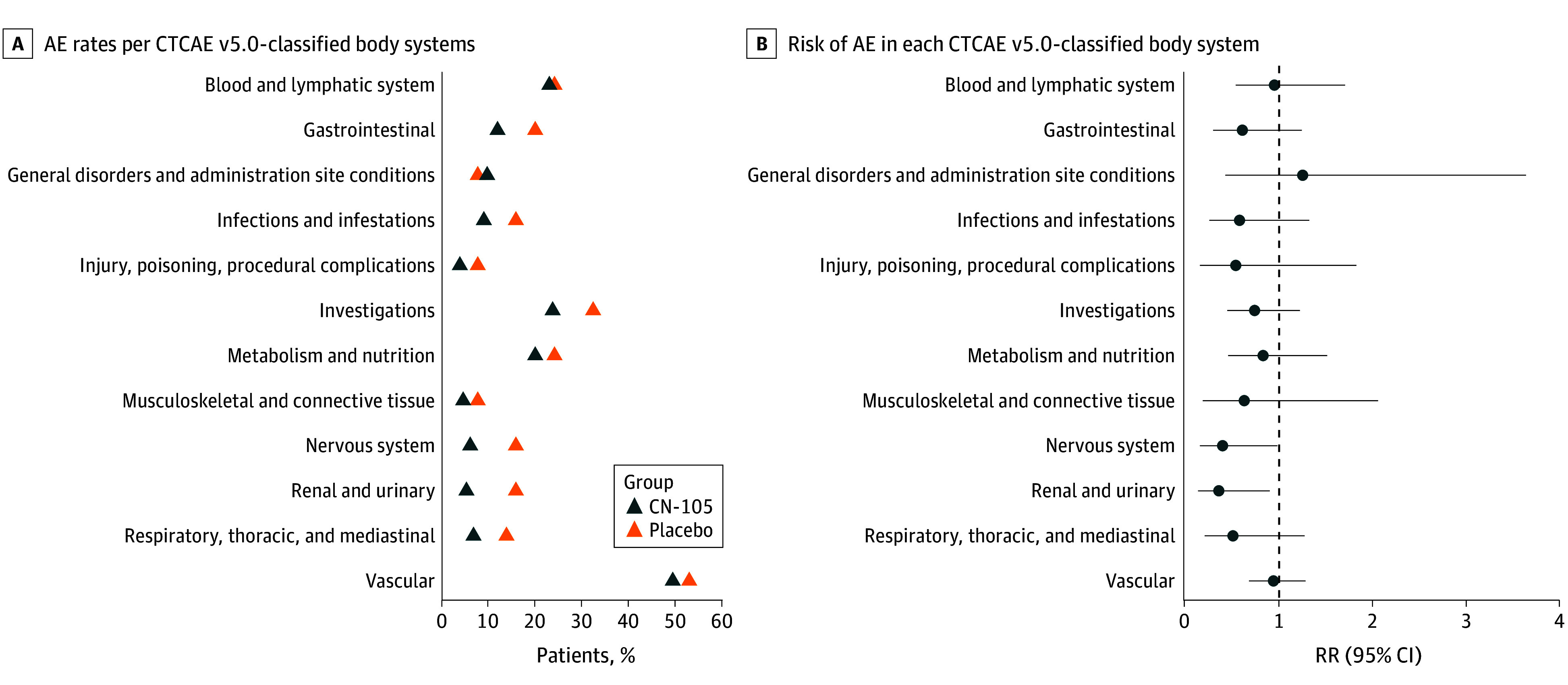
Dot Plots of Adverse Event (AE) Rates Per Common Terminology Criteria for Adverse Events, Version 5.0 (CTCAE v5.0)–Classified Body Systems With AE Rates of At Least 5% A, Comparison of the percentage of CN-105 vs placebo patients who had 1 or more AEs. B, The relative risk (RR) of an adverse event in each body system by randomization group is shown. RR 95% CI ranges that do not cross 1 (dashed line), as shown for the nervous system and renal and urinary body systems, were statistically significant. The investigations body system refers to laboratory or physiologic measurements.

There was no difference in AE rates by CTCAE v5.0–classified body system between the CN-105 and placebo groups (Figure 2). Patients in the CN-105 group compared with the placebo group had a lower RR of grade 2 or higher AEs within the renal and urinary systems (RR, 0.36; 95% CI, 0.14-0.90; *P* = .04, adjusted *P* = .43) and nervous system (RR, 0.40; 95% CI, 0.16-0.98; *P* = .04, adjusted *P* = .43) (Figure 2). A summary of AEs by surgical service, randomization group, and CN-105 dose level in the renal and urinary systems and nervous system is reported in eTables 4 to 6 in Supplement 2.

The CN-105 group compared with the placebo group had a lower incidence of SAEs within the vascular system (RR, 0.09; 95% CI, 0.01-0.78; *P* = .02, adjusted *P* = .13) and respiratory, thoracic, and mediastinal systems (RR, 0.09; 95% CI, 0.01-0.78; *P* = .02, adjusted *P* = .13) (eTable 7 in Supplement 2). There were no deaths in either group during study follow-up. The eResults in Supplement 2 provide additional information on hospital and intensive care unit length of stay as well as incidence and number of grade 3 or greater AEs

### Feasibility

The rate of study doses administered within the time window was 94.6% (860 of 909; 95% CI, 92.9%-96.0%) in the CN-105 group and 93.8% (346 of 369; 95% CI, 90.8%-96.0%) in the placebo group (χ^2^ *P* = .65 for between-groups comparison). These rates exceeded our prespecified feasibility threshold of greater than 90% of doses administered within the time window, demonstrating that CN-105 was feasible to administer in this perioperative setting.

### Postoperative Delirium and Cognitive Function

**Figure 3.**
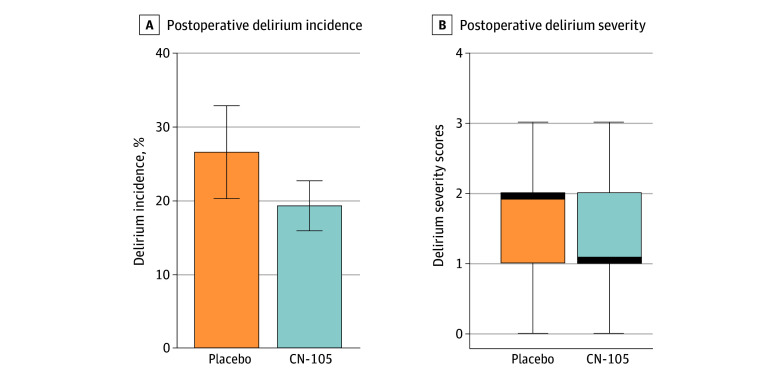
Bar and Box and Whisker Plots of CN-105 vs Placebo Effect on Postoperative Delirium Incidence and Postoperative Delirium Severity A, Error bars represent the SEs. B, The thick dark lines represent the median delirium severity scores in each group, the upper and lower ends of the boxes represent the 25th and 75th percentiles of the data, and the error bars represent 1.5 times the IQRs.

Two patients did not undergo delirium assessments due to early postoperative hospital discharge, and the overall delirium rate in this study was 21.2%. Delirium rates were 19.3% (26 of 135) in the CN-105 group and 26.5% (13 of 49) in the placebo group (Figure 3), with an RR for delirium of 0.73 (95% CI, 0.41-1.30) and odds ratio (OR) of 0.66 (95% CI, 0.31-1.42; χ^2^ *P* = .29) among patients in the CN-105 vs placebo group. In a multivariable logistic regression, the OR for delirium incidence among the CN-105 vs placebo group was 0.61 (95% CI, 0.27-1.37; *P* = .23) (eTable 8 in Supplement 2). Furthermore, when an interaction term for the effect of *APOE* ε4 carrier status by randomization group was added to this model, it was not significant.

In a sensitivity analysis excluding day-of-surgery delirium assessments (ie, conducted in the postanesthesia care unit and in the evening on the day of surgery, which were not performed in cohort 1), similar results were observed. Delirium rates were 10.2% (13 of 127) for the CN-105 group and 17.4% (8 of 46) for the placebo group, with an RR for delirium of 0.59 (95% CI, 0.26-1.33; χ^2^ *P* = .20).

The median (IQR) peak delirium severity scores for CN-105 vs placebo groups were 1 (1-2) vs 2 (1-2) (Wilcoxon *P* = .19) (Figure 3). In a univariable proportional odds model, the OR for an increase in delirium severity in the CN-105 vs placebo groups was 0.66 (95% CI, 0.37-1.20; *P* = .18). Similarly, in a multivariable proportional odds model, the OR for an increase in delirium severity in the CN-105 vs placebo groups was 0.69 (95% CI, 0.37-1.28; *P* = .24) (eTable 9 in Supplement 2). Furthermore, when an interaction term for the effect of *APOE* ε4 carrier status by randomization group was added to this model, it was insignificant. eTables 10 and 11 in Supplement 2 show CN-105 dose-specific effects on delirium incidence and severity.

There was no difference in mean (SD) global CCI change from before surgery to 6 weeks after surgery between the CN-105 and placebo groups (0.06 [0.34] vs 0.08 [0.33]; *P* = .80). There was also no effect of CN-105 on individual cognitive domain score changes over this interval (eTable 12 in Supplement 2). CN-105 also had no effect on postoperative CCI change in a multivariable-adjusted model (eTable 13 in Supplement 2), and individual CN-105 dose levels had no effect on postoperative CCI change (eTable 14 in Supplement 2).

The rate of mild postoperative neurocognitive disorder (NCD-postoperative) was 14.8% (16 of 108) in the CN-105 group vs 17.1% (6 of 35) in the placebo group (OR, 0.84; 95% CI, 0.30-2.35; χ^2^ *P* = .74). There were no instances of major NCD-postoperative.

### CSF Neuroinflammation and AD-Related Biomarkers

**Figure 4.**
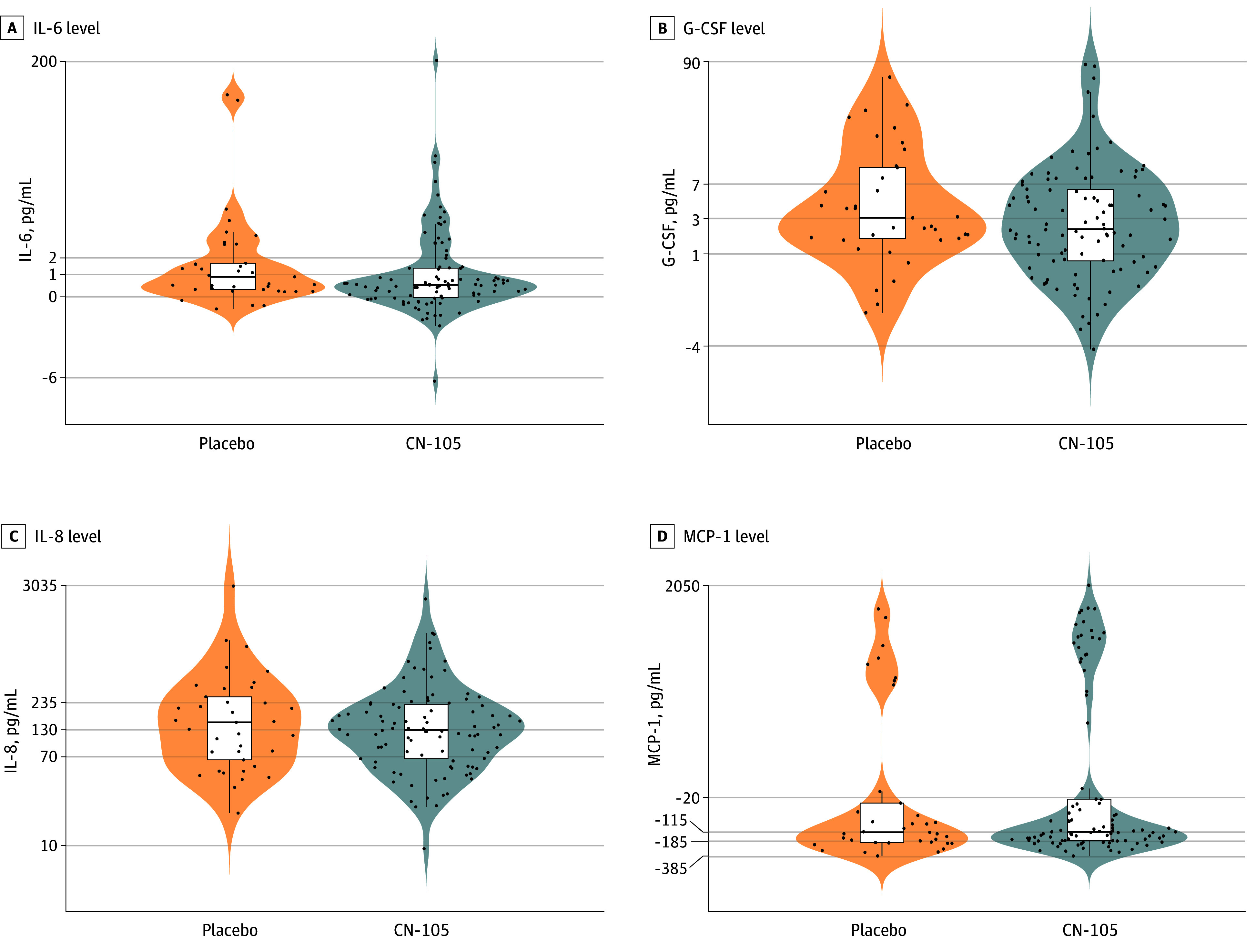
Violin and Box and Whisker Plots of the CN-105 Effect on Preoperative to 24-Hour Postoperative Changes in Cerebrospinal Fluid Cytokines The shaded violins represent the overall data distributions, the lines in the middle of the box plots represent group medians, the upper and lower ends of the boxes represent the 25th and 75th percentiles of the data, and the error bars represent 1.5 times the IQRs. The y axes were scaled via the pseudo-log function for data visualization. Tick marks were placed at the quantiles of the cytokine distribution. G-CSF indicates granulocyte-colony stimulating factor; IL-6 and IL-8, interleukin 6 and interleukin 8; and MCP-1 indicates monocyte chemoattractant protein–1.

The median difference in preoperative to 24-hour postoperative CSF cytokine-level changes for the CN-105 group vs placebo group were as follows: −0.39 pg/mL (95% CI, −0.93 to 0.14 pg/mL; *P* = .12) for interleukin (IL) 6 (IL-6); −0.84 pg/mL (95% CI, −3.06 to 1.40 pg/mL; *P* = .18) for granulocyte-colony stimulating factor (G-CSF); −23.32 pg/mL (95% CI, −94.36 to 44.93 pg/mL; *P* = .57) for IL-8; and −2.36 pg/mL (95% CI, −58.57 to 58.62 pg/mL; *P* = .50) for monocyte chemoattractant protein 1 (MCP-1) (Figure 4; eResults in Supplement 2 provides additional information). Consistent with recent work,^33^ there were no significant differences in preoperative to 24-hour postoperative changes between groups in the AD-related CSF biomarkers tau, p-tau181, or Aβ42 (eTable 15 in Supplement 2).

## Discussion

Older patients with noncardiac or nonintracranial surgery who were randomized to receive the apoE mimetic peptide CN-105 vs placebo had no increase in AE incidence, and fewer AEs and SAEs per patient were observed in this group. CN-105 levels in plasma and CSF rose with increasing CN-105 dose, and within-window administration rates of both CN-105 and placebo doses met our prespecified threshold (>90%) for feasibility of 6-hour postoperative dosing. Furthermore, there were no interaction effects between *APOE* ε4 genotype and CN-105 on the outcomes evaluated in this trial, consistent with animal studies in which CN-105’s effects were independent of *APOE* background.^11,12,13,14,15,16^

Postoperative AEs often stem from surgery-induced inflammation,^34,35,36,37^ and anti-inflammatory agents (ie, nonsteroidal anti-inflammatory drugs and corticosteroids) reduce postoperative pain and other AEs after major surgery.^38,39,40,41^ To provide a similar anti-inflammatory effect and to retain the neuroprotective properties of the endogenous apoE protein, CN-105 was designed to mimic the receptor binding face of the native helical apoE receptor binding region, yet it is small enough to cross the blood-brain barrier.^42^ Consistent with this design, although not statistically significant, the absolute magnitude of postoperative increases in each of the 4 CSF cytokines (IL-6, G-CSF, IL-8, and MCP-1) we measured was lower among patients in the CN-105 vs placebo group. Thus, the reduction in AEs per patient in the CN-105 group (vs placebo) was consistent with the anti-inflammatory mechanism of CN-105 demonstrated in preclinical studies.^11,12,13^

The overall delirium rate of 21.2% was similar to the rate reported in other large studies of delirium after noncardiac or nonintracranial surgery,^43^ and there were no significant effects on delirium in this phase 2 trial, which was not powered for efficacy outcomes. Nonetheless, the 95% CIs for the effect of CN-105 on delirium incidence ranged from a 59% decrease to a 30% increase (RR of 0.41-1.30; point estimate of a 27% reduction). A reduction in delirium incidence of more than 25% would be clinically relevant if demonstrated with statistical significance in a future trial, particularly since delirium is the most common postoperative complication among older adults and there are currently no FDA-approved drugs to prevent delirium.^44^ Prior trials have argued that even a 2% absolute reduction in delirium risk (ie, a 10% relative reduction from a 20% delirium incidence) would be a clinically important effect size.^45^ Thus, the safety, feasibility, and preliminary efficacy findings in this phase 2 trial support a phase 3 trial to determine whether CN-105 reduces postoperative delirium among older surgical patients.

### Limitations

This study has several limitations. First, generalizability may be limited due to recruitment from a single tertiary academic medical center serving predominantly non-Hispanic White older adults. Second, this cohort underwent a wide range of procedures, which may have increased variability and limited our ability to detect the effects of CN-105 on delirium and neuroinflammatory measures. Third, the sample size was modest, both overall and particularly within individual dose-level groups. The MARBLE trial was not designed or powered to draw conclusions about the most efficacious or safest CN-105 dose, although we found no evidence that any of the CN-105 dose levels (vs placebo) increased the incidence of grade 2 or higher AEs. Finally, the observed AE incidence rates were higher than anticipated, likely because rigorous CTCAE v5.0–based EMR review identified postoperative events (eg, mild hypertension) that occur commonly and are treated without major involvement by attending surgeons, who provided AE estimates on which this phase 2 trial was designed. Nevertheless, we did not observe an increase in AE incidence but found a decrease in AEs per patient in the CN-105 group vs placebo group.

## Conclusions

Overall, this MARBLE trial did not detect any meaningful safety issues for CN-105 compared with placebo in older surgical patients, showed that CN-105 resulted in fewer AEs per patient, and raised the possibility that CN-105 may lead to clinically relevant reductions in delirium incidence and severity. Several features of CN-105 are attractive in the perioperative setting, such as its linear pharmacokinetics, demonstrated safety in critically ill patients, and ability to cross the blood-brain barrier.^16,46^ Thus, properly powered phase 3 trials are warranted to definitively test the efficacy of the apoE mimetic peptide CN-105 for reducing postoperative AEs and delirium incidence and severity among older patients who underwent noncardiac or nonintracranial surgery.

## Notes

Supplement 1.
Trial Protocol and Statistical Analysis Plan

Supplement 2.
eMethods

eResults
**eTable 1.** Additional Preoperative Patient Characteristics (Intention to Treat) **eTable 2.** Multivariable Logistic Regression Results with Firth Correction for Incidence of Grade 2 or Higher Adverse Events **eTable 3.** Multivariable Negative Binomial Regression Results for the Number of Grade 2 or Higher Adverse Events per Patient **eTable 4.** Summary of Adverse Events by Surgical Service for the CTCAEv5.0-Classified Body Systems, Renal/Urinary and Nervous System **eTable 5.** Summary of Adverse Events by CTCAEv5.0-Classified Body System (Renal/Urinary and Nervous System) and Surgical Service **eTable 6.** Summary of Adverse Events by CTCAEv5.0-Classified Body System (Renal/Urinary and Nervous System) and CN-105 Dose Level, for Rates > 0 **eTable 7.** Summary of Severe Adverse Event Rates by CTCAEv5.0-Classified Body System, for Systems with ≥1 SAE **eTable 8.** Multivariable Logistic Regression Model with Firth Correction for Delirium Incidence **eTable 9.** Multivariable Proportional Odds Model for Delirium Severity **eTable 10.** Delirium Incidence by CN-105 Dose Level vs Placebo **eTable 11.** Delirium Severity by CN-105 Dose Level vs Placebo **eTable 12.** Effect of CN-105 on Change in Cognitive Domain Scores From Before to 6-Weeks After Surgery **eTable 13.** Multivariable Linear Regression Model of Preoperative to 6 Week Postoperative Cognitive Change (Continuous Cognitive Index Change) **eTable 14.** Preoperative to 6 Week Postoperative Cognitive Change (Continuous Cognitive Index Change) by CN-105 Dose Level vs Placebo **eTable 15.** Preoperative and 24 Hour Postoperative CSF Alzheimer’s Disease-Related Biomarkers (Intention to Treat) **eFigure.** Box and Whisker Plots of Blood Plasma and Cerebrospinal Fluid (CSF) Levels of CN-105 at 24 Hours After Surgery by Group (Placebo vs CN-105 Dose Levels)
eReferences

Supplement 3.
Nonauthor Collaborators

Supplement 4.
Data Sharing Statement
