# Supplementary material for: Apolipoprotein E Mimetic Peptide CN-105 and Postoperative Delirium in Older Patients: The Phase 2 MARBLE Randomized Clinical Trial
Source: JAMA Netw Open. 2026 Apr 3;9(4):e262289. doi: 10.1001/jamanetworkopen.2026.2289 (PMC13049496; doi:10.1001/jamanetworkopen.2026.2289)
Supplement: Supplement 1. — Trial Protocol and Statistical Analysis Plan [file jamanetwopen-e262289-s001.pdf]

IRB Conditions
Satisfied at the Protocol
Level
Jan 22, 2019

Modulating ApoE signalling to Reduce Brain inflammation, deLirium and postopErative cognitive dysfunction (MARBLE):

A Phase 2 Trial to Evaluate the Efficacy and Feasibility of the Apolipoprotein E Mimetic Peptide, CN-105, in Preventing Postoperative Cognitive Dysfunction and Delirium and Blocking Their Underlying Neural Mechanisms

Protocol Identifying Number: 20183129

Protocol version: 1

Principal Investigator: Miles Berger, MD, PhD

IND Sponsor: Miles Berger, MD, PhD

Funded by: Alzheimer's Drug Discovery Foundation

Draft Date: 11/13/2018

TABLE OF CONTENTS

TABLE OF CONTENTS 2
LIST OF ABBREVIATIONS 5
STATEMENT OF COMPLIANCE 7
PROTOCOL SUMMARY 8
SCHEMATIC OF TRIAL DESIGN 10
1 KEY ROLES 11
2 INTRODUCTION: BACKGROUND AND RATIONALE 11
2.1 Background Information 11
2.2 Rationale 12
2.3 Potential Risks and Benefits 12
2.3.1 Known Potential Risks 12
2.3.2 Known Potential Benefits 13
3 OBJECTIVES AND PURPOSE 13
3.1 Primary Objectives 13
3.2 Secondary Objectives 13
4 TRIAL DESIGN AND ENDPOINTS 14
4.1 Description of Trial Design 14
4.2 Trial Endpoints 15
4.2.1 Primary Endpoint 15
4.2.2 Secondary Endpoints 15
5 TRIAL ENROLLMENT AND WITHDRAWAL 16
5.1 Participant Inclusion Criteria 16
5.2 Participant Exclusion Criteria 16
5.3 Strategies for Recruitment and Retention 17
5.4 Participant Withdrawal or Termination 18
5.4.1 Reasons for Withdrawal or Termination 18
5.4.2 Handling of Participant Withdrawals or Terminations 18
5.4.3 Premature Termination or Suspension of Trial 18
6 STUDY AGENT 19
6.1 Study Agent and Control Description 19
6.1.1 Acquisition 19
6.1.2 Formulation, Appearance, Packaging, and Labeling 19
6.1.3 Product Storage and Stability 19
6.1.4 Preparation 20
6.2 Dosage and Administration 20
6.2.1 Route of Administration 20
6.2.2 Starting Dose and Escalation Schedule 20
6.2.3 Dose Adjustments/Modifications/Delays 20
6.2.4 Duration of Therapy 20
6.2.5 Tracking of Doses 21
6.3 Study Agent Accountability Procedures 21
7 TRIAL PROCEDURES AND SCHEDULE 21
7.1 Trial-Specific Procedures 21
7.1.1 Initial Lumbar Puncture and Blood Draw 21
7.1.2 Postoperative Lumbar Puncture and Blood Draws 22

7.1.3 Neurocognitive Testing, Delirium Testing, and Other Assessments 23
7.1.4 Quality of Life Assessment 24
7.1.5 Electroencephalographic Recording 24
7.2 Standard-of-Care Trial Procedures 25
7.2.1 Anesthetic Treatment 25
7.3 Laboratory Procedures/Evaluations 25
7.3.1 Clinical Laboratory Evaluations 25
7.3.2 Other Assays or Procedures 25
7.3.3 Specimen Preparation, Handling, and Storage 25
7.3.4 Specimen Shipment 26
7.4 Trial Schedule 26
7.4.1 Initial Consent and Visit 26
7.4.2 Day 0 (Day of Surgery) 26
7.4.3 Day 1 (First Day After Surgery, Hospitalization) 27
7.4.4 Days 2-4 (Hospitalization) 27
7.4.5 6 weeks (+/- 21 Days) Follow-Up 28
7.4.6 Schedule of Events 29
8 ASSESSMENT OF SAFETY 31
8.1 Specification of Safety Parameters 31
8.1.1 Definition of Adverse Events 31
8.1.2 Definition of Serious Adverse Events 31
8.1.3 Definition of Unanticipated Problems 31
8.2 Classification of an Adverse Event 32
8.2.1 Severity of Event 32
8.2.2 Relationship of Study Agent 32
8.2.3 Expectedness 33
8.3 Time Period and Frequency for Event Assessment and Follow-up 33
8.4 Reporting Procedures 33
8.4.1 Serious Adverse Event Reporting 34
8.5 Trial Halting Rules 34
8.6 Safety Oversight 34
9 CLINICAL MONITORING 35
10 STATISTICAL CONSIDERATIONS 35
10.1 Description of Statistical Methods and Analysis Plans 35
10.1.1 General Approach 35
10.1.2 Safety and Feasibility Endpoints 35
10.1.3 Analysis of the Secondary Endpoints 36
10.2 Sample Size 37
10.3 Measures to Minimize Bias 37
10.3.1 Enrollment/Randomization/Masking Procedures 37
10.3.2 Procedures to Ensure the Success of Blinding 38
11 SOURCE DOCUMENTS AND ACCESS TO DATA/DOCUMENTS 38
12 QUALITY ASSURANCE AND QUALITY CONTROL 39
13 ETHICS/PROTECTION OF HUMAN SUBJECTS 40
13.1 Ethical Standard 40
13.2 Institutional Review Board 40
13.3 Informed Consent Process 40

13.3.1 Consent and Other Informational Documents Provided to Participants 40
13.3.2 Consent Procedures and Documentation 40
13.4 Participant and Data Confidentiality 41
13.5 Research Use of Stored Human Samples, Specimens, or Data 41
14 DATA HANDLING AND RECORDKEEPING 42
14.1 Data Collection and Management Responsibilities 42
14.2 Trial Records Retention 42
14.3 Protocol Deviations 42
14.4 Publication and Data Sharing Policy 42
15 CONFLICT OF INTEREST POLICY 43
16 LITERATURE REFERENCES 43

LIST OF ABBREVIATIONS

AD	Alzheimer's disease
AE	adverse event
apoE	apolipoprotein E
BIAC	Brain Imaging and Analysis Center (Duke University)
BVMT-R	Brief Visuospatial Memory Test, Revised
CES-D	Center for Epidemiological Studies-Depression
CFR	United States Code of Federal Regulations
COWAT	Controlled Oral Word Association Test
CRF	case report form
CSF	cerebrospinal fluid
DCC	data coordinating committee
DSMB	Data Safety Monitoring Board
DUHS	Duke University Health System
DUMC	Duke University Medical Center
EC	ethics committee
EEG	electroencephalogram
FDA	Food and Drug Administration (US)
GCP	Good Clinical Practice
HSCL	Hopkins Symptom Checklist
HVLT-R	Hopkins Verbal Learning Test, Revised
ICF	informed consent form
ICMJE	International Committee of Medical Journal Editors
IRB	institutional review board
IV	intravenous
LAR	legally authorized representative
LP	lumbar puncture
MADCO-PC	Markers of Alzheimer's Disease and neuroCognitive Outcomes after Perioperative Care
MoCA	Montreal Cognitive Assessment
NIH	National Institutes of Health (US)
PI	principal investigator
POCD	postoperative cognitive decline
QOL	quality of life
SAE	serious adverse event

SC	steering committee
SF-36	Short Form-36
STAI	State Trait Anxiety Inventory
TMT	Trail Making Test
VAS	visual analog scale
WAIS-III	Wechsler Adult Intelligence Scale, 3rd Revision
WTAR	Wechsler Test of Adult Reading

STATEMENT OF COMPLIANCE

The trial will be carried out in accordance with Good Clinical Practice as required by the following:

- United States Code of Federal Regulations (CFR) applicable to clinical studies (45 CFR Part 46, 21 CFR Part 50, 21 CFR Part 56, 21 CFR Part 312 and/or 21 CFR Part 812)
- International Council on Harmonisation E6

I agree to ensure that all staff members involved in the conduct of this trial are informed about their obligations in meeting the above commitments.

Principal Investigator: Miles Berger, MD, PhD

Signed:____ Date: ____________________

PROTOCOL SUMMARY

**Title:**	**M**odulating **A**poE signaling to **R**educe **B**rain inflammation, de**L**irium and postop**E**rative cognitive dysfunction (MARBLE): A Phase 2 Trial to Evaluate the Efficacy and Feasibility of the Apolipoprotein E Mimetic Peptide CN-105 in Preventing Postoperative Cognitive Dysfunction and Delirium and Blocking Their Underlying Neural Mechanisms
**Summary:**	Participants age 60 and over who are scheduled to undergo non-cardiac, non-neurologic surgical procedures that will take more than 2 hours, and who will be admitted to the hospital after surgery, will be prospectively approached for enrollment in this trial. Participants will undergo preoperative cognitive testing and will be randomized to receive either the apolipoprotein E (apoE) mimetic peptide CN-105 or placebo. Three sequential cohorts of 67 patients will be randomized to receive CN-105 vs placebo; the CN-105 patients in each successive cohort will receive a higher/escalated dose of CN-105. Thus, we will enroll up to 201 participants to reach the target of 150 participants who complete the trial.<br>Trial participants will undergo cognitive testing and complete quality-of-life (QOL) assessments before surgery and again 6 weeks +/- 3 weeks after surgery. These participants will receive either 1 of 3 dose levels of CN-105 or placebo, starting immediately before surgery and continuing every 6 hours until postoperative day 3 or hospital discharge (whichever occurs first). We will also record noninvasive scalp electroencephalogram (EEG) data from these participants immediately before and during surgery. Cerebrospinal fluid (CSF) (up to 20 ml) and blood samples (up to 10 mL) will be collected prior to surgery (between the time of consent and the time the surgery will start), 24 +/- 2 hrs after the scheduled surgery start/anesthesia induction and 6 weeks +/- 3 weeks after the scheduled surgery start/anesthesia induction. Cerebrospinal fluid samples will be assayed for amyloid beta, tau, and other Alzheimer‘s disease (AD)-associated markers, as well as inflammatory cytokines and markers; blood samples will be assayed for serum inflammatory markers and used for APOE genotyping studies. CN-105 levels will also be measured in both CSF and peripheral blood samples. Trial participants will return to the hospital 6 weeks +/- 3 weeks later to undergo repeat cognitive testing and CSF/blood draws.
**Endpoints:**	<u>Primary:</u><br>To determine if there is significant difference in adverse events (AEs) between participants who receive CN-105 vs those who receive placebo and to determine whether it is feasible to administer 90% of study doses within the required time window (every 6 hours +/- 90 minutes).<br><u>Secondary:</u><br>1. Compare the increase in CSF inflammatory cytokines (i.e. IL-6, IL-8, MCP-1, G-CSF) from before to 24 hrs and 6 weeks after anesthesia and surgery in patients who receive CN-105 vs placebo.<br>2. Compare serum chemistries (Na, K, Cl-, HCO3-, BUN, Cr, Glucose) and blood counts (white blood cell count, hemoglobin and hematocrit, and platelet count) before, 24 hours and 6 weeks after anesthesia and surgery in patients who received CN-105 vs placebo.<br>3. Compare the change in CSF tau, phospho-tau and amyloid beta from before to 24 hours after anesthesia and surgery in patients who receive CN-105 vs placebo.

	4. Compare the change in cognition (i.e. continuous cognitive change index) from before surgery to 6 weeks after surgery in patients who receive CN-105 vs placebo.<br>5. Compare post-operative delirium rates in patients who receive CN-105 vs placebo (using the CAM-ICU in intubated patients and the 3D-CAM in non-intubated patients).<br>6. Compare changes in quality of life from before to 6 weeks after surgery in patients who receive CN-105 vs placebo (using the SF-36 instrument).<br>7. Compare intraoperative EEG spectral patterns, including "anteriorization" of 32-channel EEG alpha power and levels of burst suppression, in patients who receive CN-105 vs placebo.<br>8. To determine if the other primary and secondary outcomes differ among patients who carry the APOE4 allele or other genetic polymorphisms related to inflammation, neuroimmune function, metabolism, brain function, and/or AD.<br>Exploratory:<br>9. Describe the relationship between anesthetic exposure (duration, average hourly exposure, total exposure) and CSF inflammatory cytokine changes and demographics (age, sex, and race).<br>10. Describe the relationship between CSF changes in AD and inflammatory biomarkers, anesthetic exposure, and serum markers of metabolic and/or inflammatory status.<br>11. Describe the relationship between anesthetic exposure, CSF and serum biomarker changes, and intraoperative 32 channel EEG parameters.<br>12. Compare physical function (DASI) and mental health (STAI, SCL-90, and CES-D) before and after surgery in patients who receive CN-105 vs placebo.
**Population:**	201 participants age 60 and over who are scheduled to undergo non-cardiac, non-neurologic surgical procedures that will take more than 2 hours and who will be admitted to the hospital after surgery.
**Phase:**	2
**Sites Enrolling Participants**	Duke University Medical Center<br>Duke Regional Hospital<br>Duke Raleigh Hospital
**Description of Study Agent:**	CN-105 is an ApoE mimetic pentapeptide (AC-VSRRR-NH2). It will be supplied as a frozen liquid in 10 mL amber glass vials with a Flurotec™ stopper and a white aluminum flip off cap. Each vial contains 4 mL of CN-105 at a concentration of 12.5 mg/mL (50 mg CN-105 drug product per vial).
**Trial Duration:**	26 months
**Participant Duration:**	6 weeks +/- 3 weeks

SCHEMATIC OF TRIAL DESIGN

Patient scheduled for surgical procedures in the operation room.

↓

Consent, enrollment, randomization to CN-105 vs placebo, and preoperative cognitive testing.

↓

Collect blood and cerebrospinal fluid samples between the time of consent and anesthesia/surgery start (preoperative sample), and 24 +/- 2 hrs after anesthesia/surgery start; conduct delirium screening on each perioperative day that the participant remains in the hospital. Participants will receive either CN-105 or placebo at 6 hr intervals (for up to 13 consecutive doses) from the start of anesthesia/surgery until postoperative day 3 or hospital discharge, whichever comes first.

↓

Participants will return 6 weeks +/- 3 weeks after surgery for repeat cognitive testing, lumbar puncture, and blood draw.

↓

Intraoperative course variables from anesthesia record, EEG setup, and physiologic monitors will be obtained after the completion of the surgery.

↓

Run biomarker and genotyping assays after CSF and blood samples obtained, analyze electroencephalographic and other data.

↓

Study is complete after all assays run and all data analyzed

1 KEY ROLES

Principal Investigator:
Miles Berger, MD, PhD
Assistant Professor
Neuroanesthesiology Division
Anesthesiology Department
Duke University Medical Center
Durham, NC

Sub-Investigators:
Jeffrey Browndyke, PhD
Daniel Laskowitz, MD
Roberto Cabeza, PhD
Marty Woldorff, PhD
Niccolo Terrando, PhD

Biostatisticians:
Primary Study Statistician- Mary Cooter, MS
Independent (DSMB) Statistician- Mathew Fuller, MS
Advisory Statistician- Frank Rockhold, PhD

Clinical Laboratory:
ADNI Biomarker Core Lab, Otherwise TBD

Data Management:
Per REDCap database, under the supervision of Mary Cooter, MS

2 INTRODUCTION: BACKGROUND AND RATIONALE

2.1 Background Information

Alzheimer's disease (AD), the most common type of dementia, is an increasingly rising and alarming public health issue afflicting more than 5 million Americans (Corriveau RA et al, 2017). Epidemiologic studies have linked anesthetic and surgery exposure to the development of dementia and AD, as well as milder forms of cognitive decline (i.e., postoperative cognitive decline [POCD] and delirium) (reviewed in Berger M. et al, 2014; Berger M. et al, 2015). Furthermore, animal and human studies have shown that anesthesia and surgery increase cerebrospinal fluid (CSF) levels of AD biomarkers, such as tau, and induce neuroinflammation (see Supplemental Appendix, Figures 1 & 2, respectively). However, to date, no studies have examined the effects of interventions to block increases in these CSF AD and inflammatory biomarkers induced by anesthesia and surgery or have determined whether blocking changes in these biomarkers can improve postoperative cognitive function. We hypothesize that blocking changes in AD and neuroinflammatory biomarkers could prevent and/or at least ameliorate the risk of POCD and delirium, thus improving postoperative recovery and quality of life (QOL).

We propose to conduct a phase 2 trial in older adults undergoing non-cardiac surgery to evaluate the safety, feasibility, and efficacy of administering the apolipoprotein E (apoE) mimetic peptide, CN-105, to prevent postoperative cognitive dysfunction, delirium, and underlying pathologic neuroinflammatory and brain activity changes.

2.2 Rationale

Postoperative cognitive dysfunction (POCD) and delirium occur in up to 40% of the ≥16 million Americans over age 60 who undergo anesthesia and non-cardiac surgery each year. POCD and delirium are each associated with decreased quality of life, increased one-year postoperative mortality risk, long term cognitive decline, and a possible increased risk for dementia. There are currently no FDA-approved drugs to prevent POCD or delirium, largely because we do not understand their underlying mechanisms. Neuro-inflammation and an exacerbation of Aβ and tau pathology (i.e. AD pathology) are two mechanisms that have been identified in mouse models of POCD and delirium. Further, preliminary data (see Supplemental Appendix) suggest that neuro-inflammation and AD pathogenesis increase post-operatively in humans, potentially leading to POCD and delirium. Thus, blocking both neuro-inflammation and the acceleration of AD pathogenesis may help prevent POCD and delirium, as well as their long term sequelae. Several lines of evidence suggest that postoperative increases in neuro-inflammation and AD pathogenesis/biomarkers occur in a synergistic fashion, and together, likely contribute to delirium and POCD. Thus, an ideal drug to prevent delirium and POCD would block postoperative increases in both neuro-inflammation and AD pathogenesis/biomarkers.

The APOE4 allele, the most common genetic risk factor for late onset AD, promotes neuro-inflammation, as well as amyloid beta and tau pathology. The APOE4 allele has also been associated with long term cognitive dysfunction after anesthesia and surgery. Thus, AegisCN LLC developed the 5-amino acid peptide CN-105 to block both of these APOE4–mediated effects. AegisCN LLC's phase I study found that CN-105 is safe to administer at 1 mg/kg IV every 6 h for 14 days (Guptill J et al, 2017), which is 24-100-fold the therapeutic dose in mouse models. Here, we will conduct a phase II randomized controlled study in older surgical patients to evaluate the safety, feasibility, and efficacy of using CN-105 to prevent POCD and delirium, as well as their underlying brain function and biomarker changes. This work rests upon rigorous methods we developed in the Markers of Alzheimer's Disease and neuroCognitive Outcomes after Perioperative Care (MADCO-PC) trial to evaluate neuro-inflammation, CSF AD biomarker changes, and functional brain connectivity alterations in POCD and delirium (Giattino et al, 2017; Berger et al, manuscript in preparation, 2018; see Supplemental Appendix). In MADCO-PC, 140 older non-cardiac surgery patients underwent pre- and post-operative CSF biomarker measurements, intraoperative EEG recordings, and cognitive testing/delirium screening. Here, we will apply these methods to evaluate CN-105's efficacy in blocking perioperative increases in neuro-inflammation and CSF AD biomarkers, intraoperative brain activity alterations, and in reducing the incidence of POCD and delirium.

2.3 Potential Risks and Benefits

2.3.1 Known Potential Risks

There are currently no known potential risks associated with CN-105 administration. CN-105 was not associated with any significant adverse safety events during a completed phase I trial (Guptill J

et al, 2017) or a currently ongoing multicenter Phase II study. Both studies are evaluating CN-105 using a similar dosing regimen as that described in this protocol.

Except for LPs (discussed below), all other study procedures (e.g. blood draws, EEG recordings, etc) are standard practice and pose minimal risk to patients.

Lumbar punctures (LPs) are associated with the potential risk of a postdural headache, although this risk is less than 1% in patients over age 60 who have an LP performed with a 25g pencil-point spinal needle. Nonetheless, all participants will be given contact information for Dr. Miles Berger or the trial physician on call and encouraged to be in touch at any time if they develop a headache after the LP or any other concerning signs or symptoms. If the trial participant calls and reports that he or she has developed a headache related to the LP (i.e., a postdural puncture headache), the trial physician will assess whether this headache is likely related to the LP. If the headache is thought to be related to the LP, then per standard of care, the trial physician will then initiate conservative treatment (bed rest, increased fluid and caffeine intake, etc) for several days and/or perform an epidural blood patch (free of charge to the patient and his or her insurance).

There is also a theoretical risk of developing an infection after a lumbar puncture, although this risk is minimal when using a clean sterile kit, sterile gloves and sterile skin prep, having all individuals in the procedure area where hats and masks, and using strictly sterile technique. We have previously performed over 600 LPs with no LP-related infections in the MADCO-PC and **I**nvestigating **N**euroinflamma**T**ion **U**nderly**I**ng Postoperative Brain Connectivi**T**y Changes, POCD, Delirium in Older Adults (INTUIT) studies, further demonstrating the extremely low risk of post-LP infections.

2.3.2 Known Potential Benefits

There are currently no known benefits of CN-105 administration. Since CN-105 has been shown to block neuro-inflammation in animal models and since neuro-inflammation is thought to play an etiologic role in postoperative delirium and cognitive dysfunction, CN-105 has the potential to reduce postoperative delirium and/or cognitive dysfunction severity and/or incidence.

3 OBJECTIVES AND PURPOSE

For additional details describing the objectives, please also see the endpoints described in the Protocol Summary, as well as the Statistical Considerations in Section 10.

3.1 Primary Objectives

The primary objective of this trial is to evaluate the safety and feasibility of administering the apoE mimetic peptide, CN-105, to older adults undergoing major non-cardiac or non-neurologic surgery.

3.2 Secondary Objectives

Secondary objectives include the following:
- To determine whether CN-105 blocks postoperative neuroinflammation.
- To determine whether CN-105 has effects on serum chemistries or blood counts.

- To determine whether CN-105 blocks the increase in CSF tau levels from before to 24 hours after anesthesia and surgery and whether it modulates changes in CSF phospho-tau and amyloid beta levels from before to 24 hours after anesthesia and surgery.
- To determine whether CN-105 blocks or reduces the risk (or severity) of postoperative cognitive dysfunction.
- To determine whether participants who receive CN-105 have lower postoperative delirium rates than those who receive placebo.
- To determine whether participants who receive CN-105 (vs placebo) have improved QOL scores 6 weeks after surgery.
- To determine whether participants who receive CN-105 (vs placebo) have reduced abnormalities in intraoperative EEG spectral power patterns.
- To determine if the other primary and secondary outcomes differ among patients who carry the ApoE4 allele or other genetic polymorphisms related to inflammation, neuroimmune function, metabolism, brain function, and/or AD.

Additional exploratory objectives include:
- To describe the relationship between anesthetic exposure (both duration, average hourly exposure, total exposure) and CSF inflammatory cytokine changes and demographics (age, sex, and race).
- To describe the relationship between CSF changes in AD and inflammatory biomarkers, anesthetic exposure, and serum markers of metabolic and/or inflammatory status.
- To describe the relationship between anesthetic exposure, CSF and serum biomarker changes, and intraoperative changes in 32-channel (whole head) EEG parameters.

4 TRIAL DESIGN AND ENDPOINTS

4.1 Description of Trial Design

Trial initiation will start with the enrollment of the first participant, which will occur only after the external institutional review board (IRB) as well as the Duke University Medical Center IRB has approved the protocol.

Participants age 60 and over who are scheduled to undergo non-cardiac, non-neurologic surgical procedures that will take more than 2 hours and who are scheduled to be admitted to the hospital after surgery at Duke University Medical Center (DUMC) or one of its affiliate hospitals (Duke Regional Hospital, Duke Raleigh Hospital) will be prospectively enrolled in this trial.

Trial participants will undergo cognitive testing and complete QOL assessments within 1 month before surgery and again 6 weeks +/- 3 weeks after surgery. These participants will receive either CN-105 or placebo, starting immediately before surgery and continuing every 6 hours through postoperative day 3 (up to 13 doses maximum) or hospital discharge, whichever occurs first.

We will enroll 201 patients total in this phase 2 escalating dose study, with escalating CN-105 doses occurring in three successive groups of 67 patients each. In each group of 67 patients, 50 will receive a given dose of CN-105 and 17 will receive placebo. In our recent MADCO-PC study (Duke IRB protocol #45180), ~80% of patients returned for the 6 week assessment. Thus, enrolling 67 patients in each group ensures ≥52 patients (i.e. 39 CN-105-treated; 13 placebo treated) will

complete the 6 week +/- 3 weeks postop assessment. There will be no baseline preoperative cognitive-status inclusion/exclusion criteria. Our prior work suggests ~15% of Duke orthopedic surgery patients over age 60 have MCI, ~5% have dementia, and ~80% are cognitively normal. All primary analyses will be performed on patients grouped together independent of baseline cognitive status and dose level; secondary analyses will examine effects stratified by baseline cognitive strata, dose level, and by APOE4 carrier status. In each successive 67-patient group, the 50 drug-treated patients will receive a higher CN-105 dose: 0.1 mg/kg in the first group, then 0.5 mg/kg in the second group, and then 1 mg/kg in the third group. In each case, CN-105 (or placebo) will be given IV every 6 hours (i.e. q6 h) from 1 h prior to surgery until just prior to hospital discharge or 3 days after surgery (up to 13 doses maximum), whichever occurs first.

We will also record noninvasive electroencephalogram (EEG) data from the scalps of these participants both just before and then during anesthesia and surgery. Cerebrospinal fluid and blood samples will each be collected before surgery (between the time of consent and the time of scheduled anesthesia/surgery start), 24 hours +/- 2 hours later after the start of anesthesia/surgery, and 6 weeks +/- 3 weeks later. Cerebrospinal fluid (CSF) samples (up 20 mL each—approximately 4 teaspoons) will be collected via a sterile LP using a standard spinal anesthesia kit with a 25-g needle. Blood samples (up to 10 mL each—approximately 2 teaspoons) will be obtained at these time points as well. Blood samples will be drawn from an IV, intra-arterial line, or from a sterile venipuncture. Cerebrospinal fluid samples will be assayed for amyloid beta, tau, and other AD-associated markers, as well as for inflammatory cytokines and markers; blood samples will be assayed for serum inflammatory markers and used for genotyping studies. Trial participants will return to the hospital 6 weeks +/- 3 weeks later to undergo repeat cognitive testing, as well as CSF and blood draws.

Trial duration will be ~26 months total for participant enrollment and completion of all trial activities, with approximately 6-8 weeks duration for each individual participant, assuming an enrollment rate of ~2 patients per week. If the actual enrollment rate is slower than ~2 patients per week, the trial duration will be longer until we reach full enrollment, though the study duration per patient (~6-8 weeks) will be unchanged.

Surgical patients may participate in the trial more than once if they desire and are having a second surgery after they have already completed all trial procedures (including the 6 week postoperative time point). There are no known added risks to participants from taking part in the trial additional times, beyond the risks discussed in the informed consent form (ICF).

4.2 Trial Endpoints

4.2.1 Primary Endpoint

The primary endpoint is to determine whether there is a significant difference in adverse events (AEs) between participants in each dose cohort of CN-105 vs. those who receive placebo and to determine whether 90% of study doses are administered within the required time window (every 6 hours +/- 90 minutes).

4.2.2 Secondary Endpoints

1. Measure the increase in CSF inflammatory cytokines (i.e. IL-6, IL-8, MCP-1, G-CSF) from before to 24 hrs after and 6 weeks +/- 3 weeks after anesthesia and surgery in patients who receive CN-105 vs placebo.
2. Measure serum chemistries (Na, K, Cl-, HCO3-, BUN, Cr, Glucose) and blood counts (white blood cell count, hemoglobin and hematocrit, and platelet count) before, 24 hours after, and 6 weeks after anesthesia and surgery in patients who received CN-105 vs placebo.
3. Measure the change in CSF tau, phospho-tau, and amyloid beta from before to 24 hours (and to 6 weeks +/- 3 weeks) after anesthesia and surgery in patients who receive CN-105 vs placebo.
4. Measure the change in cognition (i.e. continuous cognitive change index) from before, immediately after, and 6 weeks +/- 3 weeks after surgery in patients who receive CN-105 vs placebo.
5. Measure postoperative delirium rates in patients who receive CN-105 vs placebo (using the CAM-ICU in intubated patients and the 3D-CAM in non-intubated patients).
6. Measure changes in quality of life from before to 6 weeks +/- 3 weeks after surgery in patients who receive CN-105 vs placebo (using the SF-36 instrument).
7. Measure intraoperative EEG spectral patterns, including previously observed "anteriorization" of 32-channel EEG alpha power (e.g., Giattino et al., 2017) and levels of burst suppression in patients who receive CN-105 vs placebo.
8. To determine if the other primary and secondary outcomes differ among patients who carry the ApoE4 allele or other genetic polymorphisms related to inflammation, neuroimmune function, metabolism, brain function, and/or AD.

Exploratory Secondary Endpoints:

1. To describe the relationship between anesthetic exposure (duration, average hourly exposure, total exposure) and CSF inflammatory cytokine changes and demographics (age, sex, and race).
2. To describe the relationship between CSF changes in AD and inflammatory biomarkers, anesthetic exposure, and serum markers of metabolic and/or inflammatory status.
3. To describe the relationship between anesthetic exposure, CSF and serum biomarker changes, and intraoperative 32 channel EEG patterns.

5 TRIAL ENROLLMENT AND WITHDRAWAL

5.1 Participant Inclusion Criteria

In order to be eligible to participate in this trial, an individual must meet all of the following criteria:

1. Age ≥ 60
2. Undergoing non-cardiac, non-neurologic surgical procedures; surgery scheduled to last > 2 hours; due to be admitted to the hospital following surgery

5.2 Participant Exclusion Criteria

An individual who meets any of the following criteria will be excluded from participation in this trial:

1. Inmate of a correctional facility
2. Scheduled to receive systemic chemotherapy between the time of the two cognitive testing sessions
3. Known inability to undergo LPs due to anticoagulant use, severe anxiety, or other clinical contraindication known ahead of time.
4. Inappropriate for study inclusion based on the judgement of the principal investigator.
5. If a patient undergoes major head trauma that occurs between the pre- and post-operative cognitive testing sessions, then they will be withdrawn from the study.

5.3 Strategies for Recruitment and Retention

Surgical patients will initially be approached in their hospital room (if they are admitted before surgery) and asked whether they are interested in hearing about the study. If the patient is not admitted before surgery, he or she will be approached in the preoperative screening clinic, preoperative optimization of surgical health, one of the surgery clinics, or approached via a phone call by a member of the clinical care team. Patients will be consented no later than the day before surgery.

We will enroll participants of both sexes and all ethnicities and racial groups. Based on the established literature regarding enrolling African-Americans and other historically underprivileged minority groups in research studies and to encourage trust and intrinsic human connections (Yancey et al, 2006), we will make every effort to have an African-American study coordinator or assistant contact African-American participants.

To help encourage study patient retention, study patients will be compensated $22 at baseline and $50 at the 6 week +/- 3 weeks follow up study visit ($25 for the 6 week postoperative cognitive testing and questionnaire completion, and $25 for undergoing the 6 week postoperative blood draw and lumbar puncture). Study patients will also receive up to $9 at each study visit (consent, pre-op, 24hr, and 6 weeks +/- 3 weeks post-op) for valet and/or parking costs. Additionally, for study patients who need transportation, the study will also pay for a wheelchair accessible van or other appropriate transportation to bring them to and from Duke for study visits. If the study pays for transportation for the patient, then the patient will not receive a parking pass or parking voucher. For patients who wish to drive themselves to Duke and who say that paying for gas would be an issue, the study will provide reimbursement for gas mileage at $0.50 per mile (up to 150 miles each way). Total possible direct subject reimbursement is thus approximately $108 for patients who live close by and do not request gas compensation, up to $72 for patients whose transportation is provided for by the study, or up to a theoretical maximum of $708 for a study subject who lives 150 miles away and requests gas compensation.

5.4 Participant Withdrawal or Termination

5.4.1 Reasons for Withdrawal or Termination

Participants may withdraw from the study at any time by contacting the study team via email or in writing. Participants who prematurely discontinue study agent treatment will still be encouraged to return for the 42-day (6 week) follow-up study visit.

Participants may also be withdrawn from the study at any time per the discretion of the principal investigator (PI).

The PI will withdraw legally authorized representative (LAR)-consented participants if they display significant distress, are uncooperative, or if the LAR-consented participant indicates in any way that he or she does not wish to proceed with the LP or other procedures. The LAR should be present during trial procedures to act as an advocate and to request withdrawal if needed.

5.4.2 Handling of Participant Withdrawals or Terminations

A recent phase I trial did not identify any specific adverse events or safety concerns with CN-105 (Guptill J et al, 2017). Nonetheless, if at any time during the dosing period, participant safety is considered at risk due to study agent-related adverse events per the judgement of the external Data Safety Monitoring Board (or DSMB+) and the PI, then the study agent will be immediately discontinued. Although the DSMB+ is only scheduled to convene and review reports (including AE and SAE information) at the completion of each 67 patient cohort, the PI may also request that the independent statistician prepare additional interim analyses and provide these to the DSMB+ at any time. For example, this may be useful and/or appropriate if there is a severe SAE (such as a patient death) and the PI wishes to have the DSMB+ review AE rates and data up to an including this severe SAE, and make interim recommendations to the study team.

Since CN-105 modulates the inflammatory response, it could theoretically impair the response to infection. Thus, in an abundance of caution, the study agent will be discontinued in any patient who develops any intracerebral or systemic infection.

If a participant has clinically significant laboratory and/or electrocardiographic abnormalities that are considered to be related to the study agent in the judgement of the Principal Investigator or the DSMB, study agent treatment discontinuation will be evaluated.

Primary study analyses will be conducted using intention to treat analysis (i.e. with patients included in the groups to which they were randomized even if they did not correctly receive all doses to which they were randomized); secondary analysis may be conducted using as treated analyses (i.e. with patients grouped by the drug doses actually received).

5.4.3 Premature Termination or Suspension of Trial

At the end of each 67 patient dose cohort, AEs will be provided to and reviewed by the DSMB+ and the PI, who will then consider whether the study can continue or whether it needs to be stopped

and/or whether other corrective action needs to take place. The external DSMB+ or the PI may also request additional interim analyses.

There will be no formal pre-specified stopping rules, particularly given the small number of patients in each successive study cohort- just 50 drug treated and 17 placebo treated patients per each 67 patient cohort. Nonetheless, the DSMB+ will have the power to halt the trial if in its judgement there is an apparent, consistent and persistent evidence of net harm that overwhelms any benefit from study drug treatment and the overall knowledge to be obtained from the study. The study may be modified at any time by the sponsor, the FDA, or the external IRB as part of their duties to ensure that research subjects are protected.

6 STUDY AGENT

6.1 Study Agent and Control Description

6.1.1 Acquisition

CN-105 will be supplied by AegisCN and manufactured under contract by University of Iowa Pharmaceuticals, Iowa City IA. Study drug will be shipped directly from the manufacturer to the Duke pharmacy, whose investigational drug service will then dispense the drug at each participating study site (Duke Hospital, Duke Regional Hospital, Duke Raleigh Hospital).

6.1.2 Formulation, Appearance, Packaging, and Labeling

CN-105 will be supplied in amber glass vials containing 4 mL of a concentrated, 12.5-mg/mL, clear-to-slightly-yellow solution. The vials are stoppered and capped with over-seals. Cloudiness, change in color, and/or the presence of any particulate matter may indicate that the product has deteriorated; if any of these or other changes are noted, the affected vials will not be used, and the study team will communicate with AegisCN and the University of Iowa Pharmaceuticals as soon as possible.

Vials of CN-105 will have the following labeling information:

- CN-105 12.5 mg/mL (50 mg/vial)
- Store frozen at -70°C
- Caution: New Drug—Limited by Federal (U.S.) Law to Investigational Use

Dosing labels will be attached to each dosing syringe/infusion bag. The individual participant syringe/infusion bag label will include both preprinted fields and handwritten fields as follows:

- Protocol number
- Personal identification number
- Participant initials
- Date
- Time
- Volume

6.1.3 Product Storage and Stability

If dosing solutions are prepared more than 1 hour before the planned start of the study drug infusion, the infusion bags containing the dosing solutions are to be refrigerated at 2 to 8°C in a secure and

locked refrigerator (and/or in a refrigerator in a secure and locked room). The drug can be refrigerated for up to 24 hours until approximately 60 minutes before the planned start of the infusion.

6.1.4 Preparation

Study drug will be diluted in normal saline by the Investigational Drug Service (IDS); placebo will be normal saline.

6.2 Dosage and Administration

We will enroll 201 patients total in this phase 2 study in three successive dose groups of 67 patients each. In each group of 67 patients, 50 will receive a given dose of CN-105, and 17 will receive placebo.

In each successive 67 patient group, participants will be randomized the week or day of surgery to receive either CN-105 (see Table 1 for dose levels) or placebo, starting immediately before surgery and continuing every 6 hours until postoperative day 3 or hospital discharge (whichever occurs first), up to a maximum of 13 doses.

6.2.1 Route of Administration

Study drug or placebo will be administered intravenously over 30 minutes.

6.2.2 Starting Dose and Escalation Schedule

Patients who receive CN-105 will receive one of three dose levels depending on their cohort: 0.1 mg/kg, 0.5 mg/kg, or 1 mg/kg, depending on whether they are in the first, second or third group of 67 patients, respectively. The 6 week +/- 3 week follow up visits for patients in the first two cohorts may be completed while the next cohort (higher dose level) is starting to be enrolled.

Table 1: Dose levels of CN-105

Cohort	Dose of CN-105	Patients receiving drug	Patients receiving placebo
Dose level 1	0.1 mg/kg	50	17
Dose level 2	0.5 mg/kg	50	17
Dose level 3	1 mg/kg	50	17

6.2.3 Dose Adjustments/Modifications/Delays

At the end of each 67-patient enrollment group, AE rates and safety information will be provided to the DSMB. Upon evaluating this information, the DSMB plus will then consider whether the study should be stopped or whether other corrective action should take place. While AE and safety information from a given 67 patient enrollment group is being analyzed and provided to the DSMB plus, and while the DSMB plus is evaluating this data (as described above), the trial will continue with enrollment of the next 67 patient group (who will receive either placebo or the next higher dose of CN-105).

There is no data from either preclinical studies or our recent phase I study suggesting any dose-dependent toxicity or side effects from CN-105. Thus, there is no reason to halt the enrollment of a subsequent 67 patient group (which will include patients receiving a higher dose of CN-105) while the DSMB is awaiting and then analyzing data from the prior 67 patient group. Nonetheless, the DSMB plus will be empowered to make recommendations about whether the trial should be stopped or modified based on their evaluation of data in the prior 67 patient group(s).

6.2.4 Duration of Therapy

Study drug will be administered every 6 hours, with the first dose 1 hour prior to surgery, until postoperative day 3 or hospital discharge, whichever occurs first, up to a maximum of 13 doses.

6.2.5 Tracking of Doses

Since the immediate pre- and post-operative periods are busy and nurses frequently have numerous tasks to perform, it is important to measure how many CN-105 (or placebo) doses are given as ordered to assess feasibility of q6 h dosing. We will track whether each CN-105 (or placebo) dose is given within +/-90 min of the scheduled administration time per nursing records.

We will report the observed rate of doses administered within the required time window and the 95% confidence interval. Each dose will be counted as an independent observation. The total number of doses correctly administered (i.e. within 90 min of the scheduled administration time) divided by the number of prescribed doses will define our dosing feasibility rate. Post-hoc feasibility analysis will be performed both among all study patients (including those receiving placebo), and specifically among CN-105 drug patients only, as well as by dose level to determine if drug dose level affects feasibility.

6.3 Study Agent Accountability Procedures

For each participant dose, details of study agent preparation and administration, i.e., time, date, dose level, volume, infusion time, etc., will be recorded in the source documents.

Upon receipt of CN-105 shipments, the pharmacist/designee will verify the condition of the vials and document per instructions in the Manual of Operational Procedures.

All used study agent vials, syringes, infusion sets, etc., will be kept in a secure location. After accountability is complete, used supplies may be discarded according to the site's normal procedures. All study vials (used/unused) remaining at the completion of the study will either be returned to the sponsor/designee or will be destroyed at the site. In case of the latter, the site will maintain destruction records.

7 TRIAL PROCEDURES AND SCHEDULE

7.1 Trial-Specific Procedures

7.1.1 Initial Lumbar Puncture and Blood Draw

An LP will be performed prior to surgery (between the time of consent and the time of scheduled anesthesia/surgery start), with a standard spinal anesthesia kit containing a 25 gauge pencil-point

needle (or other appropriate spinal needle per Good Clinical Practice) under strictly sterile conditions. This preoperative LP will be performed by an anesthesiology attending physician or senior anesthesiology resident or fellow under attending supervision, or a nurse practitioner or physician assistant under appropriate supervision.

The LP will be performed using a standard spinal anesthesia kit per the manufacturer's directions under sterile conditions at the L4–5 spinal level or the next interspace above or below this level, depending on which interspace appears larger by palpation. A curvilinear ultrasound probe will be used when necessary to help visualize the interspace. An initial 1 ml of CSF will be discarded (to reduce blood contamination of the CSF) before collecting a study sample of up to 20ml of CSF. Blood (up to 10 mL) will be collected via sterile venipuncture, from an arterial line, or by the IV line placed before surgery. To assess their level of pain following the lumbar puncture procedure, subjects will be asked to answer the following question: How much did the lumbar puncture hurt (on a scale of 0-10, where 0 is no pain at all and 10 is the worst pain imaginable?'

7.1.2 Postoperative Lumbar Puncture and Blood Draws

LPs at 24 hours and at 6 weeks +/- 3 weeks after the start of anesthesia/surgery will be performed by an anesthesiology attending physician or senior anesthesiology resident or fellow under attending supervision, or a nurse practitioner or physician assistant under appropriate supervision. To assess their level of pain following each lumbar puncture procedure, subjects will be asked to answer the following question: How much did the lumbar puncture hurt (on a scale of 0-10, where 0 is no pain at all and 10 is the worst pain imaginable?'

If the participant is hospitalized overnight after the surgery, the 24-hour postoperative LP may be performed in the participant's hospital room, in the preoperative holding area, or in other appropriate space for performing a sterile neuraxial procedure per Good Clinical Practice (GCP) guidelines. If the participant is unexpectedly discharged after surgery on the same day and does not spend the first night in the hospital, the patient will be asked to return for these procedures, and then the 24-hour postoperative LP will be performed in the same location as the 6 week +/- 3 weeks postoperative LPs are performed or in another safe location for performing sterile neuraxial anesthetic procedures per GCP guidelines. As for the preop LP, an initial 1 ml of CSF will be discarded (to reduce blood contamination of the CSF) before collecting up to a 20ml CSF sample for the study.

The 6 week +/- 3 week postoperative LPs will also be performed in the preoperative or postoperative holding area, or in other clinical research space of the Duke Anesthesiology department that is appropriately outfitted for human subject procedures (i.e. presence of medical gases, suction, resuscitation equipment. As with the the preop and 24 hr postop LPs, an initial 1 ml of CSF will be discarded (to reduce blood contamination of the CSF) before collecting up to a 20ml CSF sample for the study.

Vital signs will be taken from standard American Society of Anesthesiology monitors (heart rate, noninvasive blood pressure cuff, and pulse oximeter) by the trial team before and after the LP at the 6 week +/- 3 week postoperative time point. The LP will then be performed at the L4–5 interspace or the interspace above or below this level, depending on which space feels more open by palpation and/or appears more open by ultrasound visualization. The LP will be performed using a spinal

anesthesia kit per the manufacturer's directions under sterile conditions. The participant will then be continuously monitored for 15 minutes or longer if deemed necessary.

Blood will be drawn by sterile venipuncture, or from an arterial or an IV line if already in place, at the same time as the lumbar punctures.

7.1.3 Neurocognitive Testing, Delirium Testing, and Other Assessments

Neurocognitive testing and assessments will occur preoperatively (baseline) and at 6 weeks +/- 3 weeks after surgery. The following standardized tests and assessments will be utilized, which have equivalent alternate forms (to minimize test-retest practice effects) and have English, French and Spanish language versions:

a. Hopkins Verbal Learning Test, Revised (HVLT-R) - an auditory-verbal word-list task used to assess learning, short-term recall, long-term recall and recognition memory that is co-normed with the BVMT-R (see immediately below).
b. Brief Visuospatial Memory Test, Revised (BVMT-R) – a visual measure of constructional praxis, spatial/figure learning, short-term recall, long-term recall and recognition memory that is co-normed with the HVLT-R (see immediately above).
c. Digit Span subtest from the Wechsler Adult Intelligence Scale, 3rd Revision (WAIS-III) – a test of immediate auditory-verbal recall and complex attention (i.e., working memory).
d. Digit Symbol Coding subtest from the Wechsler Adult Intelligence Scale, 3rd Revision (WAIS-III) - a matching-to-sample visual scanning and visuomotor production task.
e. Trail Making Test (TMT), Parts A & B – a test of simple visuomotor scanning and complex sequencing and set-switching executive demands.
f. Controlled Oral Word Association Test (COWAT) – a test of lexical verbal fluency and information retrieval efficiency.
g. Grooved Pegboard Test – a timed test of bilateral manual dexterity and motor speed.
h. Wechsler Test of Adult Reading (WTAR) – a non-phonemic reading test used to assess crystalized pronunciation knowledge; a reliable estimate of premorbid intellectual functioning.
i. MoCA: used to screen for cognitive impairment.
j. STOP-Bang brief questionnaire: to assess sleep apnea risk during one of the participants' trial visits.
k. Visual analogue scale (VAS) pain assessment at each trial visit (preoperative, 6 weeks +/- 3 weeks postop.

Other assessments performed at both the preoperative and 6 week (+/- 3 week) postop visits will include:

a. List of medications participants are currently taking at each trial visit.
b. Timed up and Go task/test, Romberg assessment, and falls assessments. In particular, patients will undergo a brief falls risk assessment questionnaire, which contains questions from the Elderly Falls Screening test (Cwikel J et al, Disabil Rehab, 1998) and the Fall Risk Screening test (Tromp et al, J Clin Epidemiology, 2001), and questions about whether they have fallen recently.

Relevant tests listed above may be recorded using a digital voice recorder for quality assurance purposes. Quality assurance activities will include reviewing of testing audio files, generating performance review documents, and providing feedback on accuracy and completeness. There is no risk to the trial participants since participant identifiers will not be provided. Trial participants will not be identified by name on the recording. Only the trial number, test date, and initials of the tester will be provided on the digital file label. The link between trial number and participant identifiers will be maintained. These files will be stored on locked computers within locked offices to which only authorized research personnel will have access. These files will be destroyed after the primary outcome analysis of this research trial is complete.

Delirium Testing will be performed twice daily starting on the first day after surgery, as well as at the baseline study visit and the 6 week +/- 3 weeks postoperative study visit, using the CAM-ICU instrument in intubated patients (Ely EW et al, 2001) and the 3D-CAM instrument in non-intubated patients (Marcantonio E et al, 2014), based on the enhanced sensitivity of these instruments in these respective patient populations (Kuczmarska A et al, 2016). Inter-rater reliability for these assessments will be assessed as previously described (Marcantonio E et al, 2014).

7.1.4 Quality of Life Assessment

Our approach to measuring QOL for this trial involves the use of a battery of well-tested and well-validated instruments that together cover the relevant domains of interest. For this trial, we propose to use an assessment instrument that the Randt group developed, the SF-36 generic QOL instrument, from their longer instruments used in the Health Insurance Experiment and in the Medical Outcomes Study, as well as functional status measures (the Duke Activity Status Index [DASI]); psychological measures, including depression (the Center for Epidemiological Studies- Depression [CES-D]), the State Trait Anxiety Inventory [STAI], and the Hopkins Symptom Checklist [HSCL] (SCL-90); and social/role functioning (including employment status).

Participants will complete these questionnaires during the baseline trial visit after completing all consent paperwork and again at the 6 week +/- 3 weeks postoperative visit. Any participant who is unable to complete this questionnaire at either trial visit will be given an e-copy or a paper copy of the questionnaire to fill out at home and to mail back to our trial team at his or her convenience (in the case of paper questionnaires). Mail in questionnaires will be accepted from patients as long as they are completed by the patient within +/- 3 weeks of the preoperative study visit and before the surgery, or if they are completed within +/- 3weeks of the 6 week +/- 3 week postoperative study visit.

7.1.5 Electroencephalographic Recording

Scalp recordings of electrical activity produced by the brain (EEGs) will be obtained for 3-15 minutes before the start of anesthesia, as well as during anesthesia and surgery. The EEG will be recorded from 32 electrodes embedded in an elastic scalp electrocap, using a state-of-the-art, battery-operated, portable EEG system (Live-amps, Brain Vision LLC, Morrisville, NC). The electroencephalographic data will be analyzed to determine the effect of CN-105 on EEG correlates of postoperative cognitive function and post-operative delirium, as well as to identify possible novel pre- and intraoperative EEG correlates of POCD, delirium, and/or neuroinflammation (with the latter as measured by CSF inflammatory cytokine levels at 24 hours after anesthesia and surgery).

7.2 Standard-of-Care Trial Procedures

7.2.1 Anesthetic Treatment

All participants will receive either general anesthesia or sedation (i.e. monitored anesthesia care); patients may also receive regional or neuraxial nerve blocks as adjuncts.

The duration of anesthetic exposure shall be recorded for each participant, and intraoperative anesthetic medication administration and physiologic monitoring data will be extracted from the electronic anesthetic records for post-hoc analyses.

7.3 Laboratory Procedures/Evaluations

7.3.1 Clinical Laboratory Evaluations

A basic metabolic panel (i.e. Na^+, K^+, Cl^-, CO^2, BUN, Cr and glucose) and a complete blood count (with differential) will be measured at three time points: within 2 weeks before anesthesia and surgery (and before the first dose of CN-105), 1 day after the start of anesthesia/surgery (i.e., after ~4 doses of CN-105), and at the 6 week +/- 3 weeks postoperative study visit.

7.3.2 Other Assays or Procedures

We will measure CN-105 levels in both the serum and CSF at the 24-hour postoperative time point. This will help us determine how the CN-105 doses administered to patients in this study translate into serum drug concentrations, and will help us determine the extent of CN-105 penetration in the human CNS (as assayed in the CSF). We note that we do not plan to perform these measurements at the preoperative time point (as there should be no CN-105 in the serum or blood before the first dose is administered), or at the 6 week +/- 3 weeks postoperative study appointment (as the last CN-105 dose will be given no later than on postoperative day 3, and thus CN-105 levels would be expected to be undetectable ~6 weeks later).

We will perform serum and CSF CN-105 measurements together with our colleagues at MPI research (Mattawan, MI; now part of Charles River Laboratories), with whom AegisCN has previously performed these measurements. In brief, CN-105 will be detected via a previously validated high-performance liquid chromatography-tandem mass spectrometry assay (LC-MS/MS). The liquid chromatography system uses an ACE 3 C8 column, 2.1 x 50 mm (3 µm particle size) with an isocratic flow consisting of water/acetonitrile/heptafluorobutyric acid (76/24/0.05, v/v/v) at a flow rate of 200 µL/minute (Agilent 1200 Binary Pump) and a post column infusion consisting of isopropanol/acetic acid (100/1, v/v) at a flow rate of 800 µL/minute (Agilent 1100 LC Quaternary Pump). The analyte and internal standard (acebutolol hydrochloride) are detected using a SCIEX API 5000 triple quadrupole LC-MS/MS system equipped with an ESI (TurboIonSpray®) ionization source operated in the positive ion mode. Multiple reaction monitoring transitions of the respective $[M+H]^+$ ions are used to monitor CN-105 and acebutolol hydrochloride.

7.3.3 Specimen Preparation, Handling, and Storage

Blood samples will be used for genotyping and metabolic/inflammatory marker assays. Whole-blood specimens (10 mL total volume) will be collected sometime between the patient being consented and scheduled anesthesia start time, 24 +/- 2 hours later, and 6 weeks +/- 3 weeks later.

Whole-blood specimens will be collected with EDTA as the anticoagulant, obtained either from an arterial or IV catheter or from sterile venipuncture.

Cerebrospinal fluid specimens will be analyzed for amyloid beta, tau, and other related AD-associated neural markers and inflammatory cytokines. Cerebrospinal fluid specimens will be collected when the LP is performed before surgery (i.e. between the time of consent anesthesia/surgery start), 24 hours after the start of anesthesia/surgery, and at the 6 week +/- 3 weeks follow up visit.

Cerebrospinal fluid and blood samples will be collected by a trained care provider under sterile conditions. Blood and CSF samples will be stored at -80°C. All assay measurements will be performed at DUMC or in the accredited laboratory of a collaborator. All CSF and blood samples will be held in freezers in the Department of Anesthesiology and/or DUMC research facilities.

7.3.4 Specimen Shipment

Relevant specimens will be shipped on dry ice by FedEx overnight express to relevant collaborating labs for measurements, such as to MPI/Charles River and to the ADNI biomarker core lab at the University of Pennsylvania for tau, phospho-tau, and Aβ measurements.

7.4 Trial Schedule

7.4.1 Initial Consent and Visit

The following procedures and assessments will be conducted at the time of initial study consent:

- Informed consent
- Enrollment
- Demographics (age, sex, and race)
- Medical history
- Neurocognitive testing (including WTAR; HVLT-R; BVMT-R; WAIS-III —Digit Span, Digit Symbol Coding subtests, TrailTMT, Parts A & B, COWAT and Grooved Pegboard; MoCA) as well as the STOPBang assessment, VAS pain assessment, Falls assessments, Timed Up and Go Test, and Romberg Test
- DASI
- Psychological measures (including CES-D, STAI, SCL-90, SF-36)
- Record concomitant medications administered per standard of care
- Delirium Screening with the 3D-CAM instrument

-Randomization assignments will be made within 1 week prior to the patient's scheduled surgery.

7.4.2 Day 0 (Day of Surgery)

The following procedures and assessments will be conducted at this visit:

- Vital signs will be obtained prior to surgery in preoperative holding, per standard of care.
- Standard preoperative and intraoperative ECG

- LP prior to surgery (i.e. between the time of consent and the time of scheduled anesthesia/surgery start, which means it could be done at the initial visit described above, or anytime between the time of consent at the initial visit and the time of scheduled anesthesia/surgery start on the day of surgery)
- Concomitant medications administered per standard of care.
- Collect blood sample
- Collect CSF samples
- Study drug or placebo administration (1 hour before anesthesia induction and every 6 hours post-surgery until 3 days after surgery or hospital discharge, whichever comes first, up to a maximum of 13 doses)
- EEG (within 2 hours before surgery and during surgery)
- Vital signs (including heart rate) will be obtained during surgery and entered into the anesthetic record as per GCP and standard of care per the American Society of Anesthesiology guidelines.
- Continuous heart rate monitoring will be obtained for up to 15 minutes prior to surgery and during the surgery, to assess heart rate variability (R-R interval) as an index of vagal nerve function.
- AEs will be monitored and recorded from study enrollment (before anesthesia/surgery) until the end of the 6 week +/- 3 weeks postoperative study follow up visit.

7.4.3 Day 1 (First Day after Surgery, Hospitalization)

The following procedures and assessments will be conducted at this visit:

- LP
- Concomitant medications will be administered according to standard of care
- Collect blood sample
- Collect CSF samples
- Study drug or placebo administration (every 6 hours until 3 days after surgery or hospital discharge, whichever comes first, up to 13 doses maximum)
- AEs recorded
- 3D-CAM or CAM-ICU administered

7.4.4 Days 2-4 (Hospitalization)

The following procedures and assessments will be conducted at this visit:

- Concomitant medications will be administered according to standard of care
- Study drug or placebo administration (every 6 hours until the 3rd day after surgery or hospital discharge, whichever comes first, for a maximum of 13 doses)
- AEs recorded
- 3D-CAM or CAM-ICU administered

7.4.5 6 weeks (+/- 3 weeks) Follow-Up

The following procedures and assessments will be conducted at this visit:

- Neurocognitive testing (including HVLT-R; BVMT-R; WAIS-III —Digit Span, Digit Symbol Coding subtests, TMT Parts A & B, COWAT, MoCA, and Grooved Pegboard; STOPBang (only if it wasn't performed at the preoperative visit) VAS pain assessment, Falls assessments, Timed Up and Go Test, Romberg Test)
- Laboratory assessments
- DASI
- Psychological measures (including CES-D, STAI, Hopkins SCL-90)
- SF-36
- LP
- Collect blood samples
- Collect CSF samples
- AEs recorded
- Delirium assessment (using either the 3D-CAM instrument, or the CAM-ICU instrument in the unlikely case that the patient is intubated at the time of this study visit.
- Perform STOP-Bang sleep apnea screening assessment, if it was not already completed at the baseline study visit or at another point during the study.

7.4.6 Schedule of Events

Table 2: Schedule of Study Events for Trial

	**Screening/ Baseline Study Visit**	**Within 1 week before surgery**	**Day 0 Surgery**	**Day 1 [a] Hospitalization**	**Days 2-4 Hospitalization**	**6 weeks (+/- 21 days) Follow-up**
Informed consent	X					
Enrollment	X					
Randomization		X				
Demographics (age, sex, and race)	X					
Vital signs						X [b]
Medical history	X					
Neurocognitive testing [c]	X					X
Continuous Heart Rate Recording			X			
Laboratory assessments (additional routine clinical pre- + post-op labs will also be recorded)			X	X		X
Duke Activity Status Index	X					X
Psychological/physical measures [d]	X					X
Short Form-36	X					X
Lumbar puncture, Collect CSF samples			X [e]	X		X
Concomitant medications	X		X	X	X	X
Collect blood sample			X [f]	X		X
Drug administration [g]			X	X	X	
Electroencephalogram (EEG)			X [h]			
AEs/serious AEs			X	X	X	X
3D-CAM or CAM-ICU for delirium screening	X			X	X	X
Biomarker assays			X	X		X
Genotyping assays	X					
Inflammatory protein analysis			X	X		X
CSF protein analysis			X	X		X

	AE, adverse event; CSF, cerebrospinal fluid;<br>QOL, quality of life<br>[a] 24 (+/- 2) hours postsurgery.<br>[b] Before and after the LP, vital signs will be recorded by the study team. At other time points when the patient is already in the hospital, vital signs will already be obtained in the hospital per standard of care for inpatients.<br>[c] Includes Hopkins Verbal Learning Test-Revised; BVMT-R ; Wechsler Adult Intelligence Scale-III—Digit Span, Digit Symbol subtests; Wechsler Test of Adult Reading; Trail Making Test Parts A & B; Grooved Pegboard; Montreal Cognitive Assessment (MoCA); STOPBang Questionnaire; visual analog scale pain assessment;<br>[d] Includes Center for Epidemiological Studies-Depression, State Trait Anxiety Inventory, Hopkins Symptom Checklist, Falls assessments, Timed Up and Go, and Romberg Assessments.<br>[e] Between the time of consent and the scheduled anesthesia/surgery start time on the day of surgery.<br>[f] Between the time of consent and the scheduled anesthesia/surgery start time on the day of surgery.<br>[g] 1 hour before anesthesia induction and every 6 hours postsurgery until 3 days after surgery or hospital discharge, whichever comes first, for a maximum of 13 doses.<br>[h] Immediately before and during surgery/anesthesia.

8 ASSESSMENT OF SAFETY

8.1 Specification of Safety Parameters

8.1.1 Definition of Adverse Events (AEs)

An AE is any untoward medical occurrence associated with the use of a drug in a participant, whether or not considered drug related. An AE can, therefore, be any unfavorable and unintended sign, including an abnormal laboratory finding, for example, symptom, or disease temporally associated with the use of a pharmaceutical product. Diseases, signs, symptoms, or laboratory abnormalities already existing at randomization are not considered AEs unless they worsen (i.e., increase in intensity or frequency). Surgical procedures planned before randomization and the conditions leading to the need for these surgical procedures are not AEs.

8.1.2 Definition of Serious Adverse Events

An AE is considered serious if any of the following occur or the investigator believes any of the following outcomes may occur:

1. Death.
2. Life-threatening AE: places the participant, in the view of the investigator, at immediate risk of death at the time of the event as it occurred. It does not include an AE that, had it occurred in a more severe form, might have caused death.
3. Persistent or significant disability or incapacity or substantial disruption of the ability to conduct normal life functions.
4. Congenital abnormality or birth defect.
5. Requires inpatient hospitalization or prolongation of existing hospitalization with the following exceptions:
    - Preplanned (before the trial) hospital admissions, unless the hospitalization is prolonged
    - Planned admissions (as part of a trial, e.g. routine biopsies)
    - Re-hospitalizations (which occur less than 24 hours after discharge from index hospitalization)
    - Elective procedures
    - Emergency room visits
6. Important medical events that may not result in death, be life threatening, or require inpatient hospitalization may be considered a serious AE (SAE) when, based on appropriate medical judgment, they may jeopardize the participant and may require medical or surgical intervention to prevent one of the serious outcomes listed above. This determination is based on the opinion of the investigator.

8.1.3 Definition of Unanticipated Problems

Unanticipated problems involving risks to participants or others include, in general, any incident, experience, or outcome that meets all of the following criteria:

- Unexpected in terms of nature, severity, or frequency given (a) the research procedures that are described in the protocol-related documents, such as the IRB-approved research protocol and informed consent document; and (b) the characteristics of the participant population being studied
- Related or possibly related to participation in the research ("possibly related" means there is a reasonable possibility that the incident, experience, or outcome may have been caused by the procedures involved in the research)
- Suggests that the research places participants or others at a greater risk of harm (including physical, psychological, economic, or social harm) than was previously known or recognized

8.2 Classification of an Adverse Event

8.2.1 Severity of Event

Assessment of severity of an event will be based on the following:

- **Mild**: Is generally transient, does not interfere with usual activities, and is easily tolerated by the participant.
- **Moderate**: Causes sufficient discomfort and interferes with usual activities.
- **Severe**: Causes considerable interference with usual activities and may be incapacitating.

Whenever appropriate and/or possible, we will define AE's by the NIH Common Terminology Criteria for Adverse Events (available online at :https://ctep.cancer.gov/protocolDevelopment/electronic_applications/docs/CTCAE_v5_Quick_Reference_8.5x11.pdf)

8.2.2 Relationship of Study Agent

Assessment of the relationship of an event to the study agent will be based on the following:

- Unrelated = None, Remote
    - None (unrelated, not related, no relation)
        - Unreasonable temporal relationship between administration of the study agent and the onset of the AE (e.g., the event occurred either before, or too long after, administration of the study agent for it to be considered drug related)
        - Causal relationship between the study agent and the AE is biologically implausible (e.g., death as a passenger in an automobile accident)
        - Clearly more likely alternative explanation for the AE is present (e.g., typical adverse reaction to a concomitant drug and/or typical disease-related event)
    - Remote (unlikely, doubtful, improbable)

      Little evidence to suggest there is a causal relationship and there is another reasonable explanation for the event.

- Related = Possible, Probable

- Possible
  - There is some evidence to suggest a causal relationship (e.g., the event occurred within a reasonable time frame after administration of the study agent); however, the influence of other factors may have contributed to the event (e.g., the participant's clinical condition, other concomitant medications, or concurrent illness)
- Probable
  - There is evidence to suggest a causal relationship between the event and the study agent, and the influence of other factors is unlikely.

The investigator should consider the following when assessing causality:

- Temporal associations between the study agent and the event
- Pre-existing risk factors or concurrent illness
- Plausible mechanism

8.2.3 Expectedness

The investigator will be responsible for determining whether an AE is expected or unexpected. An AE will be considered unexpected if the nature, severity, or frequency of the event is not consistent with the risk information previously described for the study agent in the protocol.

8.3 Time Period and Frequency for Event Assessment and Follow-up

The investigator will record all reportable events with start dates occurring any time after informed consent is obtained through the last trial-related procedure/visit. At each trial visit, the investigator will inquire about the occurrence of AE/SAEs since the last visit. Events will be followed for outcome information until resolution or stabilization. Adverse events for participants who discontinue trial participation should be collected/reported through at least the time of discontinuation.

When additional relevant information (final diagnosis, outcome, results of specific investigations, etc.) becomes available, the investigator will submit this additional information to the DSMB at the time of the next DSMB report (or earlier, if deemed appropriate by the PI).

8.4 Reporting Procedures

All AEs that occur between the time informed consent is obtained and the last study-related procedure completed as part of the three month follow up visit will be recorded on the appropriate CRF forms. Adverse events may be volunteered by subjects and/or solicited at each assessment by the investigator or designee.

Recording will be performed in a concise manner using standard, acceptable medical terms. The research team (and the Principal Investigator) will make every effort to establish a diagnosis of the event based on the presenting signs, symptoms, and/or clinical information. In such cases, the diagnosis will be documented as an AE/SAE.

Diseases, signs, symptoms, or laboratory abnormalities already existing at enrollment will not considered AEs unless they worsen (i.e., increase in intensity or frequency). Surgical procedures planned before enrollment and the conditions leading to these measures are not AEs.

If, in the investigator's judgment, a clinically significant worsening from baseline is observed in any laboratory or other test parameter (e.g., ECG, angiogram), physical examination finding, or vital sign, a corresponding clinical AE should be recorded on the AE pages of the CRF. If the cause is not known, the abnormal test or finding should be recorded as an AE, using appropriate medical terminology (e.g., thrombocytopenia, peripheral edema, QT prolongation).

8.4.1 Serious Adverse Event Reporting

8.4.1.1 Reporting to the DSMB and IRB

All SAEs occurring between the time informed consent is obtained through the last study-related procedure completed as part of the 6 week (+/-3 week) follow up visit, whether or not deemed drug-related or expected, will be reported by the investigator or qualified designee within 1 working day of first becoming aware of the event to the IRB and DSMB.

8.4.1.2 Reporting to Regulatory Authorities

All SAEs will be reviewed for expectedness by the Principal Investigator or another investigator designated by the Principal Investigator. SAEs determined to be related to the investigational drug and that are unexpected according to the protocol will be reported in an expedited manner to the FDA, in accordance with 21 CFR 312.32. For unexpected drug-related SAEs that result in death or are considered life threatening, they will be reported to the FDA as soon as possible but no later than 7 days after the event. For all other unexpected, drug-related SAEs, reporting to the FDA will occur no later than 15 days after the event.

8.5 Trial Halting Rules

At the end of each 67 patient dose cohort, AEs will be provided to and reviewed by the DSMB, which will then consider whether the study can continue or whether it needs to be stopped and/or other corrective action needs to take place.

8.6 Safety Oversight

A DSMB composed of clinical/translational neuroscience investigators from outside of Duke (i.e. a DSMB+) will be convened before trial initiation and will evaluate the incidence of AEs in the placebo vs CN-105 treatment groups at the completion of each 67 patient cohort, as enrollment for the next 67 patient study cohort (and the next successive higher dose of CN-105) is beginning.

The DSMB+ will be empowered to halt the study during enrollment of the next 67 patient cohort if the following two conditions are met during the DSMB+ review of the prior 67 patient study cohort. First, that there is an apparent, consistent and persistent evidence of net harm (i.e. AE's or SAEs that occur at significantly increased frequency in patients who receive CN-105 vs placebo and which are viewed as likely resulting from CN-105 treatment rather than other factors or chance), and second that this net harm is perceived to overwhelms any benefit from study drug treatment and the overall knowledge to be obtained from the study. In this way, participants can be prevented from receiving

higher doses of CN-105 if we determine that lower doses are consistently associated with an increased incidence of AEs.

9 CLINICAL MONITORING

Clinical site monitoring will be conducted by the on-site study team to ensure that the rights and well-being of human subjects are protected, that the reported trial data are accurate, complete, and verifiable, and that the conduct of the trial is in compliance with the currently approved protocol/amendments, with GCP, and with applicable regulatory requirements. The study team will perform this monitoring at least on a quarterly basis.

10 STATISTICAL CONSIDERATIONS

10.1 Description of Statistical Methods and Analysis Plans

10.1.1 General Approach

Data will be analyzed using standard analysis programs, including SAS and SPSS. R may also be used if necessary.

10.1.2 Safety and Feasibility Endpoints

Safety Analysis: Our DSMB will examine all AEs in CN-105 and placebo treated patients. The expected rate of moderate and severe adverse events in this patient population is 5% each, based on historical Duke orthopedic surgery data; thus we expect similar AE rates in this trial in placebo-treated patients. At the end of each 67 patient enrollment group, the DSMB will examine AE rates in drug-treated vs placebo-treated patients, and consider whether the study should be stopped or whether other corrective action should take place. An independent statistician will prepare summary reports (including all AE rates in drug vs placebo treated patients) for review by the DSMB+ at the end of each 67 patient enrollment group. Thus, the study team (and the primary study statistician) will thus remain blinded to patient treatment assignment throughout the course of the study.

Assuming that we continue with all three dose groups, at study completion we will have ≥117 total CN-105 treated patients and ≥39 placebo-treated patients. Given the aforementioned expected AE rates in placebo-treated patients, at completion we will have >80% power (with a=0.05) in a two-sample two-sided unpooled variance chi-square test to detect an absolute difference of 14.8% between AE rates seen in placebo vs CN-105 treated patients. We will also track the rates of other AEs recorded by the patient's surgical team in the medical record; at study completion we will use 2 sided t tests to compare the rates of any other AEs between placebo vs CN-105 treated patients.

Feasibility Analysis: We will report the observed rate of doses administered within the required time window and the 95% confidence interval. Each dose will be counted as an independent observation. The total number of doses correctly administered (i.e. within 90 min of the scheduled administration time) divided by the number of prescribed doses will define our dosing feasibility rate. Post-hoc feasibility analysis will be performed among all patients, as well as specifically in patients receiving the study drug only, and by dose level to determine if the drug itself or drug dose level affects feasibility.

10.1.3 Analysis of the Secondary Endpoints

CSF Inflammatory Cytokine Analysis: Non-parametric 2-sided tests will be used to detect differences in CSF inflammatory cytokine increases from before surgery to 24 h after surgery in CN-105 vs placebo treated patients. For our primary analysis, we will group all three CN-105 drug dose groups together into one larger group of ≥117 CN-105 treated patients. In unpublished data from the MADCO-PC study, we observed a pre- to 24 h post-op CSF IL-6 change of 21.55 pg/ml (SD=44.72; log normal distribution; see Supplement Appendix Figure 2). Markov-chain Monte Carlo simulations based on these data demonstrate that this study (with ≥ 117 CN-105 treated and ≥ 39 placebo treated patients) will have >80% power (with α=0.05) in a 2-sided Wilcoxon Rank Sum test to detect a 34% smaller increase in IL-6 levels from before to 24 h after surgery in CN-105 vs placebo treated patients. Based on our preliminary MADCO-PC data, this sample size will also provide >80% power to detect a 36% smaller increase in G-CSF, a 32% smaller decrease in IL-8, and a 44% smaller decrease in MCP-1 levels from before surgery to 24 h after surgery in CN-105 vs placebo-treated patients. A post-hoc Kruskal-Wallis analysis will be used to compare the change in each of these inflammatory cytokines from before surgery to 24 h after surgery by CN-105 dose level. We will also examine CSF inflammatory cytokine levels 6 weeks +/- 3 weeks after surgery in all study patients to evaluate whether CN-105 treatment results in a long lasting reduction of CSF inflammatory cytokine levels.

Cognitive Data Analysis: We will compare continuous cognitive index change scores (Newman MF et al, 2001) between patients who receive CN-105 vs placebo using a 2-sided t-test. Based on the cognitive change variance seen in MADCO-PC patients (SD=0.21), this study (with ≥117 CN-105 treated and ≥ 39 placebo treated patients) will provide 80% power with α=0.05 to detect a half SD difference (i.e. 0.1 units) in CCI score between CN-105 vs placebo treated patients. Post-hoc ANOVA will determine if there are differences in cognitive index values by CN-105 dose level. We will also perform a chi-squared analysis (or Fisher's exact test if needed) to determine whether the rate of POCD as a dichotomous outcome (defined by a 1 SD drop in any of the 4 cognitive domains we have previously studied- 1- verbal memory, 2- visual memory, 3- attention/concentration, and 4- executive function) differs between CN-105 vs placebo treated patients.

Delirium analysis: Patients will be divided into those with delirium (i.e., 1 or more positive delirium assessments) vs those without delirium (no positive delirium assessments). We will compare delirium rates between CN-105 treated and placebo treated patients using a two-sided chi-square test. With an expected delirium rate of 40% in placebo treated patients, this study (with >117 CN-105 treated patients, and >39 placebo treated patients) will provide 80% power to detect a 25% lower absolute rate of delirium in CN-105 vs placebo treated patients. Post-hoc ANOVA testing will evaluate for differences in delirium rates between CN-105 drug dose level groups, with post-hoc pairwise comparison tests as appropriate. Additional post-hoc analyses will evaluate delirium duration and severity between CN-105 vs placebo treated patients.

Quality of Life Analysis: We will compare SF-36 QOL scores via 2-sided parametric or non-parametric tests as appropriate. Based on the prelim data described above, this study with >117 CN-105 treated and >39 placebo treated patients will provide >95% power with α=0.05 to detect a difference of 8.25 (16.5%) between SF-36 scores 6 weeks after surgery in CN-105 vs placebo treated patients. This effect would be similar in size to the QOL improvement seen after etanercept treatment in older adults with rheumatoid arthritis (Bae et al, 2013; Almeida et al 2016), and would thus be

clinically significant. Since etanercept, like CN-105, works by blocking inflammation, it is thus plausible to expect a similar effect size of CN-105 treatment on postoperative QOL in older adults. Post-hoc analyses will be used to confirm that there were no baseline/preoperative differences in SF-36 scores between placebo vs CN105 treated patients; a post-hoc ANOVA will examine potential differences in postoperative SF36 scores by CN-105 dose level.

10.2 Sample Size

We will enroll 201 patients total in this phase 2 escalating dose study, in three successive groups of 67 patients each. In each group of 67 patients, 50 will receive a given dose of CN-105 and 17 will receive placebo. In our recent MADCO-PC study, ~80% of patients returned for the 6 week assessment. Thus, enrolling 67 patients in each group ensures ≥52 patients (i.e. 39 CN-105-treated; 13 placebo-treated) will complete the 3 month postop assessment. There will be no baseline preoperative cognitive status inclusion/exclusion criteria. Our prior work suggests ~15% of Duke orthopedic surgery patients over age 60 have MCI, ~5% have dementia, and ~80% are cognitively normal. All primary analyses here will be performed on patients grouped together (independent of baseline cognitive status); secondary analyses will examine effects stratified by baseline cognitive strata, dose level, and by APOE4 carrier status.

Given the aforementioned expected 5% AE rates in placebo-treated patients, at completion we will have >80% power (with a=0.05) in a two-sample two-sided unpooled variance chi-square test to detect an absolute difference of 14.8% between AE rates seen in placebo vs CN-105 treated patients (pooling the three dose levels of CN-105 together). We will also track the rates of other AEs recorded by the patient's surgical team in the medical record; at study completion we will use 2-sided t tests to compare the rates of any other AEs between placebo vs CN-105 treated patients.

10.3 Measures to Minimize Bias

10.3.1 Enrollment/Randomization/Masking Procedures

Participants will be randomized to receive either CN-105 or placebo after they consent to participate in the trial, within 1 week prior to their index surgery. Participants will be enrolled in 1 of 3 dose cohorts (see Table 1) of 67 subjects each. An independent statistician will prepare dose stratified mixed block size randomization lists in a 3 to 1 ratio to achieve the desired distribution of drug and placebo. In each block of 67 trial participants, 50 will receive a given dose of CN-105 and 17 will receive placebo, in a double-blind fashion. CN-105 will be dissolved in normal saline, and either it or normal saline placebo will be administered as an IV drip over 15-30 minutes.

CN-105 and placebo will be prepared in identical bags labelled simply with the trial participant's number (1–201) to keep all trial staff and clinicians blinded to randomization. Only the investigational pharmacist will have the randomization list that explains which drug (CN-105 or placebo) each participant is to receive and what dose of CN-105 or placebo will be administered every 6 hours (+/- 90 minutes) starting within 1 h before the start of anesthesia and continuing until the end of postoperative day 3 (or when the participant is discharged home, whichever comes first), up to a maximum of 13 doses. In order to minimize bias, investigators conducting the laboratory assays on the CSF and blood samples will be blinded to trial group assignment during the course of the trial.

10.3.2 Procedures to Ensure the Success of Blinding

All personnel directly involved with study subjects (e.g., the PI, nurses, study coordinator, laboratory staff) will be blinded to treatment assignment (i.e. CN-105 vs placebo). All assays will be performed by laboratory staff blinded to treatment group/randomization.

The independent study statistician will prepare a randomization assignment log/schedule for the study, which only he and the investigational pharmacy staff will have access to- neither the PI, the primary study statistician, the co-investigator(s) or any other members of the study team will have access to the randomization assignment log/schedule. The randomization assignment log/schedule will be saved in a password protected file, to which only the independent study statistician and the investigational pharmacy staff will have access. The randomization assignment log/schedule will not be pulled up on a computer screen anytime the PI, primary study statistician, co-investigator(s) or any other members of the study team are in the room. Pharmacy personnel who will prepare the study drug will be un-blinded (by necessity). Pharmacy personnel will prepare infusion bags of CN-105 and placebo and label each bag with the subject number for whom it was intended.

For DSMB reports, the primary study statistician will pull data in a blinded fashion and give it to the independent study statistician, who will prepare a report containing analysis of the unblinded data for the DSMB plus.

In the event of blinding failure, we will document the exact nature of the unblinding incident and take it into account during study analysis.

The independent statistician will share the randomization list with the primary study statistician only upon the completion of all 6 week postoperative follow-up visits, and only after all data has been double-entered into the study redcap database and checked for accuracy by both the primary study statistician and/or the PI (or another individual blinded to randomization and selected/designated to complete this task by the PI). The primary study statistician (Ms. Mary Cooter) will remain blinded through the duration of the study, and will be unblinded (and receive a copy of the randomization assignment log/schedule) only once all assays have been completed and all data have been double entered into the study database (with any disagreements resolved), i.e. once the final study data set is completed and locked. Only at this point will the primary study statistician be unblended, in order to complete the final study data analysis.

11 SOURCE DOCUMENTS AND ACCESS TO DATA/DOCUMENTS

Source data are all information, original records of clinical findings, observations, or other activities in a clinical trial necessary for the reconstruction and evaluation of the trial. Examples of these original documents and data records include, but are not limited to, CRFs, hospital records, clinical and office charts, laboratory notes, memoranda, participant records, recorded audio tapes of counseling sessions, recorded data from automated instruments, copies or transcriptions certified after verification as being accurate and complete, microfiches, photographic negatives, microfilm or magnetic media, x-rays, and participant files and records kept at the pharmacy, laboratories, and medico-technical departments involved in the clinical trial. Access to source data will be limited to members of the research team who have undergone appropriate regulatory training and who are listed on the IRB protocol for this study.

To ensure that anyone who would access the patient medical record has adequate knowledge that the patient is participating in a clinical trial, the study coordinator will enter a note into each consented patient's Duke medical record indicating that the patient has been consented into this study.

12 QUALITY ASSURANCE AND QUALITY CONTROL

The principle investigator and study team will ensure that this clinical trial is conducted according to written SOPS and that data are generated, documented (recorded), and reported in compliance with the protocol, GCP, and the applicable regulatory requirements (e.g., Good Laboratory Practices, Good Manufacturing Practices).

The study team will provide direct access to all trial-related sites, source data/documents, and reports for the purpose of monitoring and auditing by local and regulatory authorities.

The clinical trial personnel will receive training in maintenance of all of the following:

1. Personnel signature log
2. Correspondence log (e-mail and phone)
3. Trial protocol and amendments signed by investigator
    - Copy of IRB/EC-approval
    - Copy of IRB/EC-approved ICF and amendments
    - Copy of laboratory certification
    - Copy of investigator's curriculum vita
    - Copy of IRB/EC membership that approved the trial and name of the chair (if possible)
    - Copy of training and audit reports
4. Updated subject enrollment/completed log
5. File with the identification numbers of case report forms (CRFs) corresponding to serum/CSF samples
6. File of completed CRFs and signed ICFs

Case report forms will be locked in the PI's office, the offices of Clinical Anesthesiology Research Endeavors staff, or stored on Duke University Health System (DUHS) or Department of Anesthesiology computer servers within the Duke firewall.

Quality control procedures will be implemented beginning with the data entry system, and data QC checks that will be run on the database will be generated. Any missing data or data anomalies will be communicated to the site for clarification/resolution.

The accuracy of data analysis will be verified by each investigator reviewing the primary data and its analysis. The accuracy and precision of laboratory assays will be assured with the measurement of appropriate controls for each assay. Investigators conducting the laboratory assays will be blinded as to a participant's (and sample's) trial arm assignment, thus eliminating the possibility of investigator bias while conducting these assays.

13 ETHICS/PROTECTION OF HUMAN SUBJECTS

13.1 Ethical Standard

The investigator will ensure that this trial is conducted in full conformity with regulations for the protection of human subjects of research codified in 45 Code of Federal Regulations [CFR] Part 46, 21 CFR Part 50, 21 CFR Part 56, and/or the International Council on Harmonisation E6.

13.2 Institutional Review Board

The protocol, ICF, recruitment materials, and all participant materials will be submitted to the IRB for review and approval. Approval of both the protocol and the consent form must be obtained before any participant is enrolled. Any amendment to the protocol will require review and approval by the IRB before the changes are implemented to the trial. All changes to the consent form will be IRB approved; a determination will be made regarding whether previously consented participants need to be reconsented.

13.3 Informed Consent Process

13.3.1 Consent and Other Informational Documents Provided to Participants

Consent forms describing in detail the study agent, trial procedures, and risks are given to the participant, and written documentation of informed consent is required before administering study product.

13.3.2 Consent Procedures and Documentation

Patients will be consented by one of the trial investigators or coordinators, all of whom are trained in participant enrollment. If the patient consents to participate in the trial, the trial coordinator will then set up an appointment for the patient to have preoperative or baseline cognitive testing.

Informed consent will be obtained from each participant himself or herself whenever possible. In those situations in which a participant is not competent to give informed consent, then the LAR will provide the consent on behalf of the participant. We will initially use consent of the LAR for participants deemed not to have medical decision-making capacity; however, should these participants regain medical decision-making capacity after enrollment, they will be reconsented before continuation of participation in trial. The participant’s capacity to consent will be assessed at each research visit by a qualified licensed person per DUHS policy.

If a participant is able to give informed consent to participate in the trial but physically cannot sign the consent form (due to blindness, hand injury, or other physical impairment), the participant’s LAR may sign the consent form on behalf of and at the direction of the participant. In such cases, the participant must be able to understand the potential risks, benefits, and alternatives to trial participation, and all questions and concerns of the participant will be addressed. Participants or their LARs will sign all consent paperwork before taking part in any trial procedures or cognitive testing.

13.4 Participant and Data Confidentiality

Participant confidentiality is strictly held in trust by the participating investigators, their staff, and the sponsor and their agents. This confidentiality is extended to cover testing of biological samples in addition to the clinical information relating to participants. Therefore, the trial protocol, documentation, data, and all other information generated will be held in strict confidence. No information concerning the trial or the data will be released to any unauthorized third party without previous written approval of the sponsor.

Representatives of the IRB or pharmaceutical company supplying study product may inspect all documents and records required to be maintained by the investigator, including but not limited to, medical records (office, clinic, or hospital) and pharmacy records for the participants in this trial. The clinical trial site will permit access to such records.

The trial participant's contact information will be securely stored for internal use during the trial. At the end of the trial, all records will continue to be kept in a secure location for as long a period as dictated by the Duke IRB and institutional regulations.

Trial participant research data, which is for purposes of statistical analysis and scientific reporting, will be kept in the Duke Anesthesiology department. This will not include the participant's contact or identifying information. Rather, individual participants and their research data will be identified by a unique trial identification number. The trial data entry and trial management systems used by clinical sites and by research staff will be secured and password protected. At the end of the trial, all trial databases will be de-identified and archived in the Duke Anesthesiology Department.

13.5 Research Use of Stored Human Samples, Specimens, or Data

Data collected for this trial will be analyzed and stored at the Duke Anesthesiology Clinical Anesthesia Research Endeavours offices, as appropriate. After the trial is completed, the deidentified, archived data will be stored for use by other researchers, including those outside of the trial. Permission to transmit data to this data repository will be included in the informed consent.

With the participant's approval and as approved by local IRBs, de-identified biological samples will be stored at Duke with the same goal as the sharing of data with collaborating investigators. These samples could be used for research into the causes of POCD and delirium, as well as AD, their complications and other conditions for which older surgical patients are at increased risk, and to improve treatment. Our repository will also be provided with a code-link that will allow linking the biological specimens with the phenotypic data from each participant, maintaining the masking of the identity of the participant.

During the conduct of the trial, an individual participant can choose to withdraw consent to have biological specimens stored for future research. However, withdrawal of consent with regard to biosample storage will not be possible after the trial is completed.

When the trial is completed, access to trial data and/or samples will be controlled by the Duke Neurologic Outcomes Research Group.

14 DATA HANDLING AND RECORDKEEPING

14.1 Data Collection and Management Responsibilities

Forms describing the collection of CSF and plasma aliquots will be completed at every sample draw. Case report forms will be completed at the time of enrollment and during the operative procedure. Data collected will incorporate variables reported in the subject's medical record and anesthetic record as dictated by standard of care practices and will include, but not be limited to, vital signs, imaging studies, neurological exam, records of any postoperative infections or antibiotic usage, microbiological culture data, records of any delayed wound healing or other wound healing issues, medications, presence of mechanical ventilation and/or tracheostomy, laboratory profiles, ECGs, and type of diet.

14.2 Trial Records Retention

Trial documents will be retained for a minimum of 2 years after a marketing application is approved for the drug, or, if an application is not approved for the drug, until 2 years after shipment and delivery of the drug for investigational use is discontinued and FDA has been notified. Per Duke IRB regulations, all trial documents will be retained for 6 years after the completion of the study.

14.3 Protocol Deviations

A protocol deviation is any noncompliance with the clinical trial protocol, GCP, or manual of procedures requirements. The noncompliance may be on the part of either the participant, the investigator, or the trial site staff. As a result of deviations, corrective actions are to be developed by the site and implemented promptly.

These practices are consistent with ICH E6:

- 4.5 Compliance with Protocol, sections 4.5.1, 4.5.2, and 4.5.3
- 5.1 Quality Assurance and Quality Control, section 5.1.1
- 5.20 Noncompliance, sections 5.20.1, and 5.20.2.

It is the responsibility of the study team to use continuous vigilance to identify and promptly report deviations to the IRB. All deviations must be addressed in trial source documents. The site PI/trial staff is responsible for knowing and adhering to their IRB requirements.

14.4 Publication and Data Sharing Policy

This trial will comply with the NIH public access policy, which ensures that the public has access to the published results of NIH-funded research. It requires scientists to submit final peer- reviewed journal manuscripts that arise from NIH funds to the digital archive PubMed Central upon acceptance for publication.

FDAAA mandates that a "responsible party" (ie, the sponsor or designated PI) register and report results of certain "applicable clinical trials". Since this is an applicable clinical trial, the study team will register the trial with clinicaltrials.gov and will report all results as required.

15 CONFLICT OF INTEREST POLICY

The independence of this trial from any actual or perceived influence, such as by the pharmaceutical industry, is critical. Therefore, any actual conflict of interest of persons who have a role in the design, conduct, analysis, publication, or any aspect of this trial will be disclosed and managed. Furthermore, persons who have a perceived conflict of interest will be required to have such conflicts managed in a way that is appropriate to their participation in the trial. The trial leadership has established policies and procedures for all trial group members to disclose all conflicts of interest and will establish a mechanism for the management of all reported dualities of interest.

This is a blinded study (which should reduce bias), and the study PI (Dr. Berger) himself has no COI with respect to this study, CN-105 itself, or AEGIS CN. Nonetheless since Duke University itself owns the IP on CN-105 (which was discovered and developed at Duke), Duke itself has a conflict of interest with respect to this CN-105 trial. Because of Duke's conflict of interest, the study PI (Dr. Berger) has met with the Duke University Research Integrity Office and the Conflict of Interest Committee to discuss this. The Duke Research Integrity Office and this Committee have agreed that this study can proceed at Duke under the approval of an External Institutional Review Board and the monitoring of an external DSMB plus, provided that the DSMB plus is comprised of qualified individuals from outside Duke who themselves have no other conflicts of interest, and that the study is reviewed and approved by an external IRB.

LITERATURE REFERENCES

1. Yancey AK, Ortega AN, Kumanyika SK. Effective recruitment and retention of minority research participants. *Annu Rev Public Health*. 2006;27:1-28. doi:10.1146/annurev.publhealth.27.021405.102113.

2. Monk TG, Weldon BC, Garvan CW, et al. Predictors of cognitive dysfunction after major noncardiac surgery. *Anesthesiology*. 2008;108(1):18-30. doi:10.1097/01.anes.0000296071.19434.1e.

3. Leung JM, Sands LP, Wang Y, et al. Apolipoprotein E e4 allele increases the risk of early postoperative delirium in older patients undergoing noncardiac surgery. *Anesthesiology*. 2007;107(3):406-411. doi:10.1097/01.anes.0000278905.07899.df.

4. James ML, Sullivan PM, Lascola CD, Vitek MP, Laskowitz DT. Pharmacogenomic effects of apolipoprotein e on intracerebral hemorrhage. *Stroke J Cereb Circ*. 2009;40(2):632-639. doi:10.1161/STROKEAHA.108.530402.

5. Wang H, Christensen DJ, Vitek MP, Sullivan PM, Laskowitz DT. APOE genotype affects outcome in a murine model of sepsis: implications for a new treatment strategy. *Anaesth Intensive Care*. 2009;37(1):38-45.

6. Wilcock DM, Gharkholonarehe N, Van Nostrand WE, Davis J, Vitek MP, Colton CA. Amyloid reduction by amyloid-beta vaccination also reduces mouse tau pathology and protects from neuron loss in two mouse models of Alzheimer's disease. *J Neurosci* 2009;29(25):7957-7965. doi:10.1523/JNEUROSCI.1339-09.2009.

7. Lo RY, Hubbard AE, Shaw LM, et al. Longitudinal change of biomarkers in cognitive decline. *Arch Neurol*. 2011;68(10):1257-1266. doi:10.1001/archneurol.2011.123.

8. Berger M, Nadler JW, Friedman A, et al. The Effect of Propofol Versus Isoflurane Anesthesia on Human Cerebrospinal Fluid Markers of Alzheimer's Disease: Results of a Randomized Trial. *J Alzheimers Dis JAD*. 2016;52(4):1299-1310. doi:10.3233/JAD-151190.

9. Cwikel JG, Fried AV, Biderman A, Galinsky D. Validation of a fall-risk screening test, the Elderly Fall Screening Test (EFST), for community-dwelling elderly. *Disabil Rehabil.* 1998 May;20(5):161-7.

10. Tromp AM, Pluijm SM, Smit JH, Deeg DJ, Bouter LM, Lips P. Fall-risk screening test: a prospective study on predictors for falls in community-dwelling elderly. *J Clin Epidemiol.* 2001 Aug;54(8):837-44.

11. Guptill JT, Raja SM, Boakye-Agyeman F, Noveck R, Ramey S, Tu TM, Laskowitz DT.Phase 1 Randomized, Double-Blind, Placebo-Controlled Study to Determine the Safety, Tolerability, and Pharmacokinetics of a Single Escalating Dose and Repeated Doses of CN-105 in Healthy Adult Subjects. *J Clin Pharmacol*. 2017 Jun;57(6):770-776. doi: 10.1002/jcph.853.

12. Giattino CM, Gardner JE, Sbahi FM, Roberts KC, Cooter M, Moretti E, Browndyke JN, Mathew JP, Woldorff MG, Berger M; MADCO-PC Investigators. Intraoperative Frontal Alpha-Band Power Correlates with Preoperative Neurocognitive Function in Older Adults. *Front Syst Neurosci.* 2017 May 8;11:24. doi: 10.3389/fnsys.2017.00024

13. Corriveau Ra, Koroshetz Wj, Gladman Jt, Jeon S, Babcock D Et Al. Alzheimer's Disease-Related Dementias Summit 2016: National Research Priorities. *Neurology*. 2017 Dec 5;89(23):2381-2391. Doi: 10.1212/Wnl.0000000000004717.

14. Berger M, Burke J, Eckenhoff R, Mathew J. Alzheimer's disease, anesthesia, and surgery: a clinically focused review. *J Cardiothorac Vasc Anesth.* 2014 Dec;28(6):1609-23. doi: 10.1053/j.jvca.2014.04.014

15. Berger M, Nadler JW, Browndyke J, Terrando N, Ponnusamy V, Cohen HJ, Whitson HE, Mathew JP. Postoperative Cognitive Dysfunction: Minding the Gaps in Our Knowledge of a Common Postoperative Complication in the Elderly. *Anesthesiol Clin.* 2015 Sep;33(3):517-50. doi: 10.1016/j.anclin.2015.05.008.

16. Almeida C, Choy EH, Hewlett S, et al. Biologic interventions for fatigue in rheumatoid arthritis. *Cochrane Database Syst Rev*. 2016(6): CD008334. 69.

17. Bae SC, Gun SC, Mok CC, et al. Improved health outcomes with etanercept versus usual DMARD therapy in an Asian population with established rheumatoid arthritis. BMC *Musculoskelet Disord.* 2013; 14:13.

18. Newman MF, Kirchner JL, Phillips-Bute B, Gaver V, Grocott H, Jones RH, Mark DB, Reves JG, Blumenthal JA; Neurological Outcome Research Group and the Cardiothoracic Anesthesiology Research Endeavors Investigators. Longitudinal assessment of neurocognitive function after coronary-artery bypass surgery. *N Engl J Med*. 2001 Feb 8;344(6):395-402.

19. Marcantonio ER, Ngo LH, O'Connor M, Jones RN, Crane PK, Metzger ED, Inouye SK. 3D-CAM: derivation and validation of a 3-minute diagnostic interview for CAM-defined delirium: a cross-sectional diagnostic test study. *Ann Intern Med.* 2014 Oct 21;161(8):554-61. doi: 10.7326/M14-0865.

20. Ely EW, Margolin R, Francis J, May L, Truman B, Dittus R, Speroff T, Gautam S, Bernard GR, Inouye SK. Evaluation of delirium in critically ill patients: validation of the Confusion Assessment Method for the Intensive Care Unit (CAM-ICU). *Crit Care Med.* 2001 Jul;29(7):1370-9.

21. Kuczmarska A, Ngo LH, Guess J, O'Connor MA, Branford-White L, Palihnich K, Gallagher J, Marcantonio ER: Detection of Delirium in Hospitalized Older General Medicine Patients: A Comparison of the 3D-CAM and CAM-ICU. *J Gen Intern Med* 2016; 31: 297-303

Modulating **A**poE signalling to **R**educe **B**rain inflammation, de**L**irium and postop**E**rative cognitive dysfunction (MARBLE):

A Phase 2 Trial to Evaluate the Efficacy and Feasibility of the Apolipoprotein E Mimetic Peptide, CN-105, in Preventing Postoperative Cognitive Dysfunction and Delirium and Blocking Their Underlying Neural Mechanisms

Protocol Identifying Number: 20183129

Protocol version: 7

Principal Investigator: Miles Berger, MD, PhD

IND Sponsor: Miles Berger, MD, PhD

Funded by: Alzheimer's Drug Discovery Foundation

Draft Date: 25 – May - 2022

TABLE OF CONTENTS

TABLE OF CONTENTS 2
LIST OF ABBREVIATIONS 5
STATEMENT OF COMPLIANCE 7
PROTOCOL SUMMARY 8
SCHEMATIC OF TRIAL DESIGN 11
1 KEY ROLES 12
2 INTRODUCTION: BACKGROUND AND RATIONALE 12
2.1 Background Information 12
2.2 Rationale 13
2.3 Potential Risks and Benefits 13
2.3.1 Known Potential Risks 13
2.3.2 Known Potential Benefits 14
3 OBJECTIVES AND PURPOSE 15
3.1 Primary Objectives 15
3.2 Secondary Objectives 15
4 TRIAL DESIGN AND ENDPOINTS 16
4.1 Description of Trial Design 16
4.2 Trial Endpoints 18
4.2.1 Primary Endpoint 18
4.2.2 Secondary Endpoints 18
5 TRIAL ENROLLMENT AND WITHDRAWAL 19
5.1 Participant Inclusion Criteria 19
5.2 Participant Exclusion Criteria 19
5.3 Strategies for Recruitment and Retention 19
5.4 Participant Withdrawal or Termination 22
5.4.1 Reasons for Withdrawal or Termination 22
5.4.2 Handling of Participant Withdrawals or Terminations 22
5.4.3 Premature Termination or Suspension of Trial 22
6 STUDY AGENT 24
6.1 Study Agent and Control Description 24
6.1.1 Acquisition 24
6.1.2 Formulation, Appearance, Packaging, and Labeling 24
6.1.3 Product Storage and Stability 24
6.1.4 Preparation 25
6.2 Dosage and Administration 25
6.2.1 Route of Administration 25
6.2.2 Starting Dose and Escalation Schedule 25
6.2.3 Dose Adjustments/Modifications/Delays 25
6.2.4 Duration of Therapy 26
6.2.5 Tracking of Doses 26
6.3 Study Agent Accountability Procedures 26
7 TRIAL PROCEDURES AND SCHEDULE 27
7.1 Trial-Specific Procedures 27
7.1.1 Initial Lumbar Puncture and Blood Draw 27
7.1.2 Postoperative Lumbar Puncture and Blood Draws 27

7.1.3 Neurocognitive Testing, Delirium Testing, and Other Assessments......... 28
7.1.4 Quality of Life Assessment 29
7.1.5 Electroencephalographic Recording 30
7.2 Standard-of-Care Trial Procedures 30
7.2.1 Anesthetic Treatment 30
7.3 Laboratory Procedures/Evaluations 31
7.3.1 Clinical Laboratory Evaluations 31
7.3.2 Other Assays or Procedures 31
7.3.3 Specimen Preparation, Handling, and Storage 31
7.3.4 Specimen Shipment 32
7.4 Trial Schedule 32
7.4.1 Initial Consent and Visit 32
7.4.2 Day 0 (Day of Surgery) 33
7.4.3 Day 1 (First Day After Surgery, Hospitalization) 34
7.4.4 Days 2-4 (Hospitalization) 34
7.4.5 6 weeks (+/- 21 Days) Follow-Up 34
7.4.6 Schedule of Events 36
8 ASSESSMENT OF SAFETY 38
8.1 Specification of Safety Parameters 38
8.1.1 Definition of Adverse Events 38
8.1.2 Definition of Serious Adverse Events 38
8.1.3 Definition of Unanticipated Problems 39
8.2 Classification of an Adverse Event 40
8.2.1 Severity of Event 40
8.2.2 Relationship of Study Agent 40
8.2.3 Expectedness 42
8.3 Time Period and Frequency for Event Assessment and Follow-up 42
8.4 Reporting Procedures 42
8.4.1 Serious Adverse Event Reporting 43
8.5 Trial Halting Rules 43
8.6 Safety Oversight 44
9 CLINICAL MONITORING 44
10 STATISTICAL CONSIDERATIONS 44
10.1 Description of Statistical Methods and Analysis Plans 44
10.1.1 General Approach 44
10.1.2 Safety and Feasibility Endpoints 44
10.1.3 Analysis of the Secondary Endpoints 45
10.2 Sample Size 46
10.3 Measures to Minimize Bias 46
10.3.1 Enrollment/Randomization/Masking Procedures 46
10.3.2 Procedures to Ensure the Success of Blinding 47
11 SOURCE DOCUMENTS AND ACCESS TO DATA/DOCUMENTS 48
12 QUALITY ASSURANCE AND QUALITY CONTROL 48
13 ETHICS/PROTECTION OF HUMAN SUBJECTS 49
13.1 Ethical Standard 49
13.2 Institutional Review Board 49
13.3 Informed Consent Process 49
13.3.1 Consent and Other Informational Documents Provided to Participants 49

13.3.2 Consent Procedures and Documentation 49
13.4 Participant and Data Confidentiality 50
13.5 Research Use of Stored Human Samples, Specimens, or Data 50
14 DATA HANDLING AND RECORDKEEPING 51
14.1 Data Collection and Management Responsibilities 51
14.2 Trial Records Retention.. 51
14.3 Protocol Deviations 51
14.4 Publication and Data Sharing Policy 52
15 CONFLICT OF INTEREST POLICY 52
16 LITERATURE REFERENCES 52

LIST OF ABBREVIATIONS

AD	Alzheimer's disease
AE	adverse event
apoE	apolipoprotein E
BIAC	Brain Imaging and Analysis Center (Duke University)
BVMT-R	Brief Visuospatial Memory Test, Revised
CES-D	Center for Epidemiological Studies-Depression
CFR	United States Code of Federal Regulations
COWAT	Controlled Oral Word Association Test
CRF	case report form
CSF	cerebrospinal fluid
DCC	data coordinating committee
DSMB	Data Safety Monitoring Board
DUHS	Duke University Health System
DUMC	Duke University Medical Center
EC	ethics committee
EEG	electroencephalogram
FDA	Food and Drug Administration (US)
GCP	Good Clinical Practice
HSCL	Hopkins Symptom Checklist
HVLT-R	Hopkins Verbal Learning Test, Revised
ICF	informed consent form
ICMJE	International Committee of Medical Journal Editors
IRB	institutional review board
IV	intravenous
LAR	legally authorized representative
LP	lumbar puncture
MADCO-PC	Markers of Alzheimer's Disease and neuroCognitive Outcomes after Perioperative Care
MoCA	Montreal Cognitive Assessment
NIH	National Institutes of Health (US)
PI	principal investigator
POCD	postoperative cognitive decline
QOL	quality of life
SAE	serious adverse event

SC	steering committee
SF-36	Short Form-36
STAI	State Trait Anxiety Inventory
TMT	Trail Making Test
VAS	visual analog scale
WAIS-III	Wechsler Adult Intelligence Scale, 3rd Revision
WTAR	Wechsler Test of Adult Reading

STATEMENT OF COMPLIANCE

The trial will be carried out in accordance with Good Clinical Practice as required by the following:

- United States Code of Federal Regulations (CFR) applicable to clinical studies (45 CFR Part 46, 21 CFR Part 50, 21 CFR Part 56, 21 CFR Part 312 and/or 21 CFR Part 812)
- International Council on Harmonisation E6

I agree to ensure that all staff members involved in the conduct of this trial are informed about their obligations in meeting the above commitments.

Principal Investigator: Miles Berger, MD, PhD

Signed:____Date: ____________________

PROTOCOL SUMMARY

**Title:**	**M**odulating **A**poE signaling to **R**educe **B**rain inflammation, de**L**irium and postop**E**rative cognitive dysfunction (MARBLE): A Phase 2 Trial to Evaluate the Efficacy and Feasibility of the Apolipoprotein E Mimetic Peptide CN-105 in Preventing Postoperative Cognitive Dysfunction and Delirium and Blocking Their Underlying Neural Mechanisms
**Summary:**	Participants age 60 and over who are scheduled to undergo non-cardiac, non-neurologic surgical procedures that will take more than 2 hours, and who will be admitted to the hospital after surgery, will be prospectively approached for enrollment in this trial. Participants will undergo preoperative cognitive testing and will be randomized to receive either the apolipoprotein E (apoE) mimetic peptide CN-105 or placebo. Three sequential cohorts of 67 patients will be randomized to receive CN-105 vs placebo; the CN-105 patients in each successive cohort will receive a higher/escalated dose of CN-105. Thus, we will enroll at least 201 participants to reach the target of 150 participants who complete the trial.<br><br>Trial participants will undergo cognitive testing and complete quality-of-life (QOL) assessments before surgery and again 6 weeks +/- 3 weeks after surgery. These participants will receive either 1 of 3 dose levels of CN-105 or placebo, within one hour prior to case start time and continuing every 6 hours until postoperative day 3 or hospital discharge (whichever occurs first). We will also record noninvasive scalp electroencephalogram (EEG) data from these participants immediately before, during and after surgery. Cerebrospinal fluid (CSF) (up to 20 ml) and blood samples (up to 10 mL) will be collected prior to surgery (between the time of consent and the time the surgery will start), 24 +/- 2 hr after the scheduled surgery start/anesthesia induction and 6 weeks +/- 3 weeks after the scheduled surgery start/anesthesia induction. Cerebrospinal fluid samples will be assayed for amyloid beta, tau, and other Alzheimer‘s disease (AD)-associated markers, as well as inflammatory cytokines and markers; blood samples will be assayed for serum inflammatory markers and used for APOE genotyping studies. CN-105 levels will also be measured in both CSF and peripheral blood samples. Trial participants will return to the hospital 6 weeks +/- 3 weeks later to undergo repeat cognitive testing and CSF/blood draws.
**Endpoints:**	<u>Primary:</u><br>To determine if there is significant difference in grade II or higher adverse events (AEs) between participants who receive CN-105 vs those who receive placebo.<br><br><u>Secondary:</u><br>1. Compare the increase in CSF inflammatory cytokines (i.e. IL-6, IL-8, MCP-1, G-CSF) from before to 24 hr and 6 weeks after anesthesia and surgery in patients who receive CN-105 vs placebo.<br>2. Compare the change in cognition (i.e. continuous cognitive change index) from before surgery to 6 weeks after surgery in patients who receive CN-105 vs placebo.<br>3. To determine whether it is feasible to administer 90% of study doses within the required time window (every 6 hours +/- 90 minutes).<br>4. Compare post-operative delirium rates and severity in patients who receive CN-105 vs placebo (using the CAM-ICU in intubated patients and the 3D-CAM in non- intubated patients).

	<u>Exploratory:</u><br>5. Compare the change in CSF tau, phospho-tau and amyloid beta from before to 24 hours after anesthesia and surgery in patients who receive CN-105 vs placebo.<br>6. Compare serum chemistries (Na, K, Cl-, HCO3-, BUN, Cr, Glucose) and blood counts (white blood cell count, hemoglobin and hematocrit, and platelet count) before, 24 hours and 6 weeks after anesthesia and surgery in patients who received CN-105 vs placebo.<br>7. Compare changes in quality of life from before to 6 weeks after surgery in patients who receive CN-105 vs placebo (using the SF-36 instrument).<br>8. Compare intraoperative EEG spectral patterns, including “anteriorization” of 32-channel EEG alpha power and levels of burst suppression, in patients who receive CN-105 vs placebo.<br>9. To determine if the primary and secondary outcomes differ among patients who carry the APOE4 allele or other genetic polymorphisms related to inflammation, neuroimmune function, metabolism, brain function, and/or AD.<br>10. Describe the relationship between anesthetic exposure (duration, average hourly exposure, total exposure) and CSF inflammatory cytokine changes and demographics (age, sex, and race).<br>11. Describe the relationship between CSF changes in AD and inflammatory biomarkers, anesthetic exposure, and serum markers of metabolic and/or inflammatory status.<br>12. Describe the relationship between anesthetic exposure, CSF and serum biomarker changes, and intraoperative 32 channel EEG parameters.<br>13. Compare physical function (DASI) and mental health (STAI, SCL-90, and CES-D) before and after surgery in patients who receive CN-105 vs placebo.
**Population:**	Up to 250 participants age 60 and over who are scheduled to undergo non-cardiac, non- neurologic surgical procedures that will take more than 2 hours and who will be admitted to the hospital after surgery.
**Phase:**	2
**Sites Enrolling Participants**	Duke University Medical Center<br>Duke Regional Hospital<br>Duke Raleigh Hospital
**Description of Study Agent:**	CN-105 is an ApoE mimetic pentapeptide (AC-VSRRR-NH2). It will be supplied as a frozen liquid in 10 mL amber glass vials with a Flurotec™ stopper and a white aluminum flip off cap. Each vial contains 4 mL of CN-105 at a concentration of 12.5 mg/mL (50 mg CN-105 drug product per vial).
**Trial Duration:**	Up to 50 months
**Participant Duration:**	6 weeks +/- 3 weeks

SCHEMATIC OF TRIAL DESIGN

Patient scheduled for surgical procedures in the operation room.

Consent, enrollment, randomization to CN-105 vs placebo, and preoperative cognitive testing.

Collect blood and cerebrospinal fluid samples between the time of consent and anesthesia/surgery start (preoperative sample), and 24 +/- 2 hr after anesthesia/surgery start; conduct delirium screening on each perioperative day that the participant remains in the hospital. Participants will receive either CN-105 or placebo at 6 hr intervals (for up to 13 consecutive doses) from within one hour prior to case start time until postoperative day 3 or hospital discharge, whichever comes first.

Participants will repeat cognitive testing and return for repeat lumbar puncture and blood draw 6 weeks +/- 3 weeks after surgery.

Intraoperative course variables from anesthesia record, EEG setup, and physiologic monitors will be obtained after the completion of the surgery.

Run biomarker and genotyping assays after CSF and blood samples obtained, analyze electroencephalographic and other data.

Study is complete after all assays run and all data analyzed

1 KEY ROLES

Principal Investigator:
Miles Berger, MD, PhD
Assistant Professor
Neuroanesthesiology Division
Anesthesiology Department
Duke University Medical Center
Durham, NC

Sub-Investigators:
Jeffrey Browndyke, PhD
Daniel Laskowitz, MD
Roberto Cabeza, PhD
Marty Woldorff, PhD
Niccolo Terrando, PhD

Biostatisticians:
Primary Study Statistician- Mary Cooter, MS
Independent (DSMB) Statistician- Mathew Fuller, MS
Advisory Statistician- Frank Rockhold, PhD

Clinical Laboratory:
ADNI Biomarker Core Lab, Otherwise TBD

Data Management:
Per REDCap database, under the supervision of Mary Cooter, MS

2 INTRODUCTION: BACKGROUND AND RATIONALE

2.1 Background Information

Alzheimer's disease (AD), the most common type of dementia, is an increasingly rising and alarming public health issue afflicting more than 5 million Americans (Corriveau RA et al, 2017). Epidemiologic studies have linked anesthetic and surgery exposure to the development of dementia and AD, as well as milder forms of cognitive decline (i.e., postoperative cognitive decline [POCD] and delirium) (reviewed in Berger M. et al, 2014; Berger M. et al, 2015). Furthermore, animal and human studies have shown that anesthesia and surgery increase cerebrospinal fluid (CSF) levels of AD biomarkers, such as tau, and induce neuroinflammation (see Supplemental Appendix, Figures 1 & 2, respectively). However, to date, no studies have examined the effects of interventions to block increases in these CSF AD and inflammatory biomarkers induced by anesthesia and surgery or have determined whether blocking changes in these biomarkers can improve postoperative cognitive function. We hypothesize that blocking changes in AD and neuroinflammatory biomarkers could prevent and/or at least ameliorate the risk of POCD and delirium, thus improving postoperative recovery and quality of life (QOL).

We propose to conduct a phase 2 trial in older adults undergoing non-cardiac surgery to evaluate the safety, feasibility, and efficacy of administering the apolipoprotein E (apoE) mimetic peptide, CN-105, to prevent postoperative cognitive dysfunction, delirium, and underlying pathologic neuroinflammatory and brain activity changes.

2.2 Rationale

Postoperative cognitive dysfunction (POCD) and delirium occur in up to 40% of the ≥16 million Americans over age 60 who undergo anesthesia and non-cardiac surgery each year. POCD and delirium are each associated with decreased quality of life, increased one-year postoperative mortality risk, long term cognitive decline, and a possible increased risk for dementia. There are currently no FDA-approved drugs to prevent POCD or delirium, largely because we do not understand their underlying mechanisms. Neuro-inflammation and an exacerbation of A® and tau pathology (i.e. AD pathology) are two mechanisms that have been identified in mouse models of POCD and delirium. Further, preliminary data (see Supplemental Appendix) suggest that neuro-inflammation and AD pathogenesis increase post-operatively in humans, potentially leading to POCD and delirium. Thus, blocking both neuro-inflammation and the acceleration of AD pathogenesis may help prevent POCD and delirium, as well as their long term sequelae. Several lines of evidence suggest that postoperative increases in neuro-inflammation and AD pathogenesis/biomarkers occur in a synergistic fashion, and together, likely contribute to delirium and POCD. Thus, an ideal drug to prevent delirium and POCD would block postoperative increases in both neuro-inflammation and AD pathogenesis/biomarkers.

The APOE4 allele, the most common genetic risk factor for late onset AD, promotes neuro-inflammation, as well as amyloid beta and tau pathology. The APOE4 allele has also been associated with long term cognitive dysfunction after anesthesia and surgery. Thus, AegisCN LLC developed the 5-amino acid peptide CN-105 to block both of these APOE4–mediated effects. AegisCN LLC’s phase I study found that CN-105 is safe to administer at 1 mg/kg IV every 6 h for 14 days (Guptill J et al, 2017), which is 24-100-fold the therapeutic dose in mouse models. Here, we will conduct a phase II randomized controlled study in older surgical patients to evaluate the safety, feasibility, and efficacy of using CN-105 to prevent POCD and delirium, as well as their underlying brain function and biomarker changes. This work rests upon rigorous methods we developed in the Markers of Alzheimer’s Disease and neuroCognitive Outcomes after Perioperative Care (MADCO-PC) trial to evaluate neuro-inflammation, CSF AD biomarker changes, and functional brain connectivity alterations in POCD and delirium (Giattino et al, 2017; Berger et al, manuscript in preparation, 2018; see Supplemental Appendix). In MADCO-PC, 140 older non-cardiac surgery patients underwent pre- and post-operative CSF biomarker measurements, intraoperative EEG recordings, and cognitive testing/delirium screening. Here, we will apply these methods to evaluate CN-105's efficacy in blocking perioperative increases in neuro-inflammation and CSF AD biomarkers, intraoperative brain activity alterations, and in reducing the incidence of POCD and delirium.

2.3 Potential Risks and Benefits

2.3.1 Known Potential Risks

There are currently no known potential risks associated with CN-105 administration. CN-105 was not associated with any significant adverse safety events during a completed phase I trial (Guptill J

et al, 2017) or a currently ongoing multicenter Phase II study. Both studies are evaluating CN-105 using a similar dosing regimen as that described in this protocol.

Except for LPs (discussed below), all other study procedures (e.g. blood draws, EEG recordings, etc.) are standard practice and pose minimal risk to patients.

Lumbar punctures (LPs) are associated with the potential risk of a postdural headache, although this risk is less than 1% in patients over age 60 who have an LP performed with a 25g pencil-point spinal needle (or other appropriate spinal needle per Good Clinical Practice) under strictly sterile conditions. Nonetheless, all participants will be given contact information for Dr. Miles Berger or the trial physician on call and encouraged to be in touch at any time if they develop a headache after the LP or any other concerning signs or symptoms. If the trial participant calls and reports that he or she has developed a headache related to the LP (i.e., a postdural puncture headache), the trial physician will assess whether this headache is likely related to the LP. If the headache is thought to be related to the LP, then per standard of care, the trial physician will then initiate conservative treatment (bed rest, increased fluid and caffeine intake, etc.) for several days and/or perform an epidural blood patch (free of charge to the patient and his or her insurance).

There is also a theoretical risk of developing an infection after a lumbar puncture, although this risk is minimal when using a clean sterile kit, sterile gloves and sterile skin prep, having all individuals in the procedure area where hats and masks, and using strictly sterile technique. We have previously performed over 600 LPs with no LP-related infections in the MADCO-PC and **I**nvestigating **N**euroinflamma**T**ion **U**nderly**I**ng Postoperative Brain Connectivi**T**y Changes, POCD, Delirium in Older Adults (INTUIT) studies, further demonstrating the extremely low risk of post-LP infections.

For patients undergoing surgery who have a primary cancer, such as lung, kidney, breast or prostate, if the patient has spine imaging (presumably PET or MRI) available in his/her Duke medical record, we will review that spine imaging prior to enrolling the patient in the study to make sure that the patient does not have metastatic disease in their spinal column, which could then theoretically get seeded into the intra-thecal space if the LP needle went thru the metastasis before entering the intrathecal space. If a patient has one of these cancers (lung, kidney, breast or prostate) that tend to metastasize to bone, and the patient has imaging evidence of metastatic disease in their spinal column, we will then exclude the patient from the study to avoid even the theoretical risk of seeding the intrathecal space with cancerous cells. If the patient has cancer and has not had spine imaging, that presumably means that the patient has not had back pain or other symptoms suggestive of spine metastasis and/or that the clinical team had a low enough suspicion for spine metastasis that they didn’t feel that it was necessary to obtain spine imaging on the patient. In such cases, these patients will still remain eligible for enrollment, assuming they meet other inclusion/exclusion criteria. We will not obtain spine imaging as part of the study in these situations if the primary clinical team did not order it and did not think it was indicated.

In cases in which a patient has prior spine imaging in his or her Duke medical record, a physician member of the study team will review the images (and any official interpretations) in the patient’s chart and make a determination as to whether there is severe spinal stenosis. Patients with severe spinal stenosis will be excluded from this study due to the theoretical risk of causing myelopathy or radicular symptoms by introducing a needle into the intrathecal space.

2.3.2 Known Potential Benefits

There are currently no known benefits of CN-105 administration. Since CN-105 has been shown to block neuro-inflammation in animal models and since neuro-inflammation is thought to play an etiologic role in postoperative delirium and cognitive dysfunction, CN-105 has the potential to reduce postoperative delirium and/or cognitive dysfunction severity and/or incidence.

3 OBJECTIVES AND PURPOSE

For additional details describing the objectives, please also see the endpoints described in the Protocol Summary, as well as the Statistical Considerations in Section 10.

3.1 Primary Objectives

The primary objective of this trial is to evaluate the safety of administering the apoE mimetic peptide, CN-105, to older adults undergoing major non-cardiac or non-neurologic surgery.

3.2 Secondary Objectives

Secondary objectives include the following:

- To determine whether CN-105 blocks postoperative neuroinflammation as measured by changes in levels of the following cytokines in the CSF: IL-6, IL-8, MCP-1, and G-CSF.
- To determine whether CN-105 blocks or reduces the risk (or severity) of CCI index change between CN-105 and placebo groups.
- To determine whether participants who receive CN-105 have lower postoperative delirium rates and severity than those who receive placebo.
- To determine whether it is feasible to administer 90% of study doses within the required time window (every 6 hours +/- 90 minutes).

Additional exploratory objectives include:

- To determine whether CN-105 has effects on serum chemistries or blood counts.
- To describe the relationship between anesthetic exposure (both duration, average hourly exposure, total exposure) and CSF inflammatory cytokine changes and demographics (age, sex, and race).
- To describe the relationship between CSF changes in AD and inflammatory biomarkers, anesthetic exposure, and serum markers of metabolic and/or inflammatory status.
- To describe the relationship between anesthetic exposure, CSF and serum biomarker changes, and intraoperative changes in 32-channel (whole head) EEG parameters.
- To determine whether CN-105 blocks the increase in CSF tau levels from before to 24 hours after anesthesia and surgery and whether it modulates changes in CSF phospho-tau and amyloid beta levels from before to 24 hours after anesthesia and surgery.
- To determine whether participants who receive CN-105 (vs placebo) have improved QOL scores 6 weeks after surgery.
- To determine whether participants who receive CN-105 (vs placebo) have reduced abnormalities in intraoperative EEG spectral power patterns.
- To determine if the other primary and secondary outcomes differ among patients who carry the ApoE4 allele or other genetic polymorphisms related to inflammation, neuroimmune function, metabolism, brain function, and/or AD.
- Compare physical function (DASI) and mental health (STAI, SCL-90, and CES-D) before and after surgery in patients who receive CN-105 vs placebo.

4 TRIAL DESIGN AND ENDPOINTS

4.1 Description of Trial Design

Trial initiation will start with the enrollment of the first participant, which will occur only after the external institutional review board (IRB) as well as the Duke University Medical Center IRB has approved the protocol.

Participants age 60 and over who are scheduled to undergo non-cardiac, non-neurologic surgical procedures that will take more than 2 hours and who are scheduled to be admitted to the hospital after surgery at Duke University Medical Center (DUMC) or one of its affiliate hospitals (Duke Regional Hospital, Duke Raleigh Hospital) will be prospectively enrolled in this trial.

Trial participants will undergo cognitive testing and complete QOL assessments within 2 months before surgery and again 6 weeks +/- 3 weeks after surgery. These participants will receive either CN-105 or placebo, within one hour before the scheduled or actual start time of surgery and continuing every 6 hours through postoperative day 3 (up to 13 doses maximum) or hospital discharge, whichever occurs first. Each case start time will be defined by the study team as either the start of the surgery/procedure or the start of anesthesia.

We planned to enroll up to 250 patients in this phase 2 escalating dose study, with escalating CN-105 doses occurring in three successive groups of 67 randomized patients each. Due to a clerical error by the Investigational Drug Services that prepares our drug/placebo doses, 69 patients were randomized to the first patient cohort rather than 67. Additionally, because enrollment happens at consent, and not all patients that consent will be randomized, we will keep enrolling patients until we reach 67 consented and randomized patients in each of the last two cohorts.

In each group of 67 patients, 50 will receive a given dose of CN-105 and 17 will receive placebo. In our recent MADCO-PC study(Duke IRB protocol #45180), ~80% of patients returned for the 6 week assessment. Thus, enrolling 67 patients in each group ensures ≥52 patients (i.e. 39 CN-105-treated; 13 placebo treated) will complete the 6 week +/- 3 weeks postop assessment.

There will be no baseline preoperative cognitive-status inclusion/exclusion criteria. Our prior work suggests ~15% of Duke orthopedic surgery patients over age 60 have MCI, ~5% have dementia, and ~80% are cognitively normal. All primary analyses will be performed on patients grouped together independent of baseline cognitive status and dose level; exploratory analyses will examine effects stratified by baseline cognitive strata, dose level, and by APOE4 carrier status. In each successive 67-patient group, the 50 drug-treated patients will receive a higher CN-105 dose: 0.1 mg/kg in the first group, then 0.5 mg/kg in the second group, and then 1 mg/kg in the third group. In each case, CN-105 (or placebo) will be given IV every 6 hours (i.e. q6 h) from the first dose, which will occur within one hour of the scheduled or actual surgery start time, until just prior to hospital discharge or 3 days after surgery (up to 13 doses maximum), whichever occurs first. Each case start time will be defined by the study team as either the start of the surgery/procedure or the start of anesthesia (or the timed notes in the EMR for induction, intubation, or the start of anesthetic drug administration), or such times as planned/scheduled. If the case gets delayed by more than 6 hr and we've already given 1st dose, we will give an additional dose prior to surgery, but under no conditions will the patient be given more than 13 doses total of CN-105

We will also record noninvasive electroencephalogram (EEG) data from the scalps of these participants just before, during, and right after anesthesia and surgery. In the event that sufficient personnel or equipment are unavailable, or in the event that two MARBLE patients are having surgery at the same time, or in the event that there is insufficient time to place the EEG sensor on that patient's head before the start of surgery, the study team may not record EEG from all patients or at all points before, during, and just after surgery/anesthesia.
Cerebrospinal fluid and blood samples will each be collected before surgery (between the time of consent and the time of scheduled anesthesia/surgery start), 24 hours +/- 2 hours later after the start of anesthesia/surgery, 6 weeks +/- 3 weeks later, and potentially 1 year after surgery for some patients, depending on funding and the PIs discretion. Cerebrospinal fluid (CSF) samples (up 20 mL each—approximately 4 teaspoons) will be collected via a sterile LP using a standard spinal anesthesia kit with a 25-g needle (or other appropriate spinal needle per Good Clinical Practice) under strictly sterile conditions. The study may provide funds for patients to stay at a local hotel near the hospital overnight if the patient is discharged on the day of their surgery and if driving back to Duke on post-op day 1 would not be possible or reasonable for the patient. This service will be provided based on the PI's discretion, in part based on whether sufficient funds are available to support this expense. Blood samples (up to 10 mL each—approximately 2 teaspoons) will be obtained at these time points as well. Blood samples will be drawn from an IV, intra-arterial line, or from a sterile venipuncture. Cerebrospinal fluid samples will be assayed for amyloid beta, tau, and other AD- associated markers, as well as for inflammatory cytokines and markers; blood samples will be assayed for serum inflammatory markers and used for genotyping studies. Trial participants will complete repeated cognitive testing and return to the hospital to undergo repeat CSF and blood draws 6 weeks +/- 3 weeks later, or may complete the 6 week cognitive testing by remote video if appropriate (i.e. due to the pandemic or other issues). We created a post-LP instruction sheet for patients and caregivers advising them on activities for the 24 hours following their lumbar puncture and with Dr. Berger's phone number, should they have questions or experience any complications following the LP.

Patients may complete a post-op 1 year study visit (+/-2 months) at which time we will collect blood, perform another LP, and administer the same surveys and cognitive testing that were performed at the baseline and 6 week visits, provided that sufficient funds are available per the PI's judgement at that time. If funding become available after some 1 year time point visits have already been missed, we will potentially perform 1 year visits only on the remaining patients who are still within the correct time window. Likewise, in light of uncertainties surrounding cost and funding due to COVID-19, if other financial changes occur that make it impossible to continue 1 year study visits or any other aspect of this protocol, specific patients may be discontinued based on the PI's judgement and discretion.

Trial duration will be up to 72 months total for participant enrollment and completion of all trial activities, with approximately 6-8 weeks duration for each individual participant (and up to 1 year duration for those who have a 1 year follow up performed), assuming an enrollment rate of ~2 patients per week. If the actual enrollment rate is slower than ~2 patients per week, the trial duration will be longer until we reach full enrollment, though the study duration per patient (~6-8 weeks or 1 year for those with 1 year follow up time) will be unchanged.

Surgical patients may participate in the trial more than once if they desire and are having a second surgery after they have already completed all trial procedures (including the 6 week postoperative time point). There are no known added risks to participants from taking part in the trial additional times, beyond the risks discussed in the informed consent form (ICF).

4.2 Trial Endpoints

4.2.1 Primary Endpoint

The primary endpoint is to determine whether there is a significant difference in grade II or higher adverse events (AEs) between participants in each dose cohort of CN-105 vs. those who receive placebo.

4.2.2 Secondary Endpoints

1. Measure the increase in CSF inflammatory cytokines (i.e. IL-6, IL-8, MCP-1, G-CSF) from before to 24 hr after and 6 weeks +/- 3 weeks after anesthesia and surgery in patients who receive CN-105 vs placebo.
2. Measure the change in cognition (i.e. continuous cognitive change index) from before, immediately after, and 6 weeks +/- 3 weeks after surgery in patients who receive CN-105 vs placebo.
3. Measure postoperative delirium rates in patients who receive CN-105 vs placebo (using the CAM-ICU in intubated patients and the 3D-CAM in non-intubated patients).
4. To determine whether it is feasible to administer 90% of study doses within the required time window (every 6 hours +/- 90 minutes).

<u>Exploratory Endpoints:</u>

1. To describe the relationship between anesthetic exposure (duration, average hourly exposure, total exposure) and CSF inflammatory cytokine changes and demographics (age, sex, and race).
2. To describe the relationship between CSF changes in AD and inflammatory biomarkers, anesthetic exposure, and serum markers of metabolic and/or inflammatory status.
3. To describe the relationship between anesthetic exposure, CSF and serum biomarker changes, and intraoperative 32 channel EEG patterns.
4. Measure serum chemistries (Na, K, Cl-, HCO3-, BUN, Cr, Glucose) and blood counts (white blood cell count, hemoglobin and hematocrit, and platelet count) before, 24 hours after, and 6 weeks after anesthesia and surgery in patients who received CN-105 vs placebo.
5. Measure changes in quality of life from before to 6 weeks +/- 3 weeks after surgery in patients who receive CN-105 vs placebo (using the SF-36 instrument).
6. Measure intraoperative EEG spectral patterns, including previously observed “anteriorization” of 32-channel EEG alpha power (e.g., Giattino et al., 2017) and levels of burst suppression in patients who receive CN-105 vs placebo. If necessary due to COVID-19-related issues or other safety concerns or exigencies, 1-2 channel EEG will be performed using a BIS EEG monitor (or other appropriate frontal EEG device) rather than using our standard 32 channel EEG caps to minimize patient contact.
7. To determine if the other primary and secondary outcomes differ among patients who carry the ApoE4 allele or other genetic polymorphisms related to inflammation, neuroimmune function, metabolism, brain function, and/or AD.
8. Measure the change in CSF tau, phospho-tau, and amyloid beta from before to 24 hours (and to 6 weeks +/- 3 weeks) after anesthesia and surgery in patients who receive CN-105 vs placebo.

5 TRIAL ENROLLMENT AND WITHDRAWAL

5.1 Participant Inclusion Criteria

In order to be eligible to participate in this trial, an individual must meet all of the following criteria:

1. Age ≥ 60
2. Undergoing non-cardiac, non-neurologic surgical procedures; surgery scheduled to last > 2 hours; due to be admitted to the hospital following surgery.

Note: Informed consent will be obtained from each subject whenever possible. In situations where a subject is not competent to give informed consent, then the legally authorized representative (LAR) will provide consent on behalf of the subject. We will initially use consent of the LAR for subjects deemed not to have medical decision making capacity; however, should these subjects regain medical decision-making capacity after enrollment, they will be re-consented prior to continuation of participation in study. The PI will withdraw LAR consented subjects if they display distress, are uncooperative, or if the LAR consented subject indicates in any way that he or she does not wish to proceed with the LP and other procedures. The LAR should be present at study procedures to act as an advocate and to request withdrawal if/as needed. The subject's capacity to consent will be assessed at each research visit.

5.2 Participant Exclusion Criteria

An individual who meets any of the following criteria will be excluded from participation in this trial:

1. Inmate of a correctional facility
2. Scheduled to receive systemic chemotherapy between the time of the two cognitive testing sessions
3. Known inability to undergo LPs due to anticoagulant use, severe anxiety, or other clinical contraindication known ahead of time.
4. Inappropriate for study inclusion based on the judgement of the principal investigator.
5. If a patient undergoes major head trauma that occurs between the pre- and post-operative cognitive testing sessions, then they will be withdrawn from the study.
6. If a patient has lung, kidney, breast or prostate cancer, which tend to metastasize to bone, and the patient has imaging evidence of metastatic disease in their spinal column, the patient is excluded from the study to avoid even the theoretical risk of seeding the intrathecal space with cancerous cells.
7. Patients with existing imaging in the electronic medical record showing severe spinal stenosis will be excluded from this study due to the theoretical risk of causing myelopathy or radicular symptoms by introducing a needle into the intrathecal space.

5.3 Strategies for Recruitment and Retention

Surgical patients will initially be approached in their hospital room (if they are admitted before surgery) and asked whether they are interested in hearing about the study. If the patient is not admitted before surgery, he or she will be approached in the preoperative screening clinic, preoperative optimization of surgical health, one of the surgery clinics, or approached via a phone call by a member of the clinical care team and/or the study team using IRB approved phone script. In addition, potentially eligible patients will be identified in the electronic medical record

by a report, and those patients will be sent an automated message through Duke's MyChart application briefly describing the study and including a link to contact the study team if they are interested in participating. Per the template from the Duke MyChart recruitment team, the message will also contain a link to one of our MARBLE recruitment videos in which Dr. Berger explains the study. Patients will be consented before study activities begin.

We will enroll participants of both sexes and all ethnicities and racial groups. Based on the established literature regarding enrolling African-Americans and other historically underprivileged minority groups in research studies and to encourage trust and intrinsic human connections (Yancey et al, 2006), we will make every effort to have an African-American study coordinator or assistant contact African-American participants.

To help encourage study patient retention, study patients will be compensated $50 at baseline and $50 at the 6 week +/- 3 weeks follow up study visit ($25 for the 6-week postoperative cognitive testing and questionnaire completion, and $25 for undergoing the 6 week postoperative blood draw and lumbar puncture). Study patients will also receive up to $12 at each study visit (consent, pre-op, 24hr, and 6 weeks +/- 3 weeks post-op) for valet and/or parking costs. Additionally, for study patients who need transportation, the study will also pay for a wheelchair accessible van or other appropriate transportation to bring them to and from Duke for study visits. If the study pays for transportation for the patient, then the patient will not receive a parking pass or parking voucher. For patients who wish to drive themselves to Duke and who say that paying for gas would be an issue, the study will provide reimbursement for gas mileage at $0.50 per mile (up to 150 miles each way). Total possible direct subject reimbursement is thus approximately $148 for patients who live close by and do not request gas compensation, up to $100 for patients whose transportation is provided for by the study, or up to a theoretical maximum of $748 for a study subject who lives 150 miles away and requests gas compensation.

Once patients are contacted via phone (submitted as separate phone script) and express interest in the study, we may email them a link to one of the following videos, which features Dr. Berger introducing the MARBLE study.

https://www.dropbox.com/sh/blfaucno13w2u6e/AAAKcu0wjf1wdsuJsJow5aOHa?dl=0

Because the idea of a lumbar puncture is scary for some patients, we have also created a series of videos explaining and showing an actual lumbar puncture procedure. If patients are concerned about this procedure from a safety and adverse event perspective, we can email them a link to one of the following videos that introduce the procedure.

https://duke.app.box.com/folder/87650948771

Furthermore, to promote continued patient engagement and interest in MARBLE, we have created a quarterly MARBLE newsletter with the intent of distributing them to all patients who have been consented and enrolled in MARBLE if possible. We will attempt to distribute this newsletter to patients via email or mailed through the postal service. In addition, we will attempt to give a physical copy of the current newsletter to all newly consented patients at their baseline visit, unless this is not possible due to the covid19 crisis or other exigencies. Each newsletter may contain the following: a brief synopsis of the study, study progress (recruitment goals and current recruitment attained), tips for a healthy brain, recent presentations/accomplishments from our research group, and staff member spotlight (a picture and job description of one of our research staff members and a quote from them on their perspective on the study).

We have also created an information tri-fold flier describing the MARBLE study that includes a brief synopsis of the study, eligibility criteria, what is involved in the study, and contact information for the PI, Dr. Miles Berger. Flyers will be distributed at the clinics of Duke surgeons who may have eligible patients, at the anesthesia preoperative screening clinic, and at other relevant locations where potentially eligible patients will see these flyers.

5.4 Participant Withdrawal or Termination

5.4.1 Reasons for Withdrawal or Termination

Participants may withdraw from the study at any time by contacting the study team via email or in writing. Participants who prematurely discontinue study agent treatment will still be encouraged to return for the 42-day (6 week) follow-up study visit.

Participants may also be withdrawn from the study at any time per the discretion of the principal investigator (PI).

The PI will withdraw legally authorized representative (LAR)-consented participants if they display significant distress, are uncooperative, or if the LAR-consented participant indicates in any way that he or she does not wish to proceed with the LP or other procedures, per the PI's clinical judgement. The LAR should be present during trial procedures to act as an advocate and to request withdrawal if needed, unless this is impossible due to hospital/health system regulations during the covid19 or other exigent circumstances.

5.4.2 Handling of Participant Withdrawals or Terminations

A recent phase I trial did not identify any specific adverse events or safety concerns with CN-105 (Guptill J et al, 2017). Nonetheless, if at any time during the dosing period, participant safety is considered at risk due to study agent-related adverse events per the judgement of the external Data Safety Monitoring Board (or DSMB+) and the PI, then the study agent will be immediately discontinued. Although the DSMB+ is only scheduled to convene and review reports (including grade II or higher AE, and SAE information) at the completion of each 67 patient cohort, the PI may also request that the independent statistician prepare additional interim analyses and provide these to the DSMB+ at any time. For example, this may be useful and/or appropriate if there is a severe SAE (such as a patient death) and the PI wishes to have the DSMB+ review grade II or higher AE rates and data up to an including this severe SAE, and make interim recommendations to the study team.

Since CN-105 modulates the inflammatory response, it could theoretically impair the response to infection. Thus, in an abundance of caution, the study agent will be discontinued in any patient who develops any intracerebral or systemic infection.

If a participant has clinically significant laboratory and/or electrocardiographic abnormalities that are considered to be related to the study agent in the judgement of the Principal Investigator or the DSMB, study agent treatment discontinuation will be evaluated.

Primary study analyses will be conducted using intention to treat analysis (i.e. with patients included in the groups to which they were randomized even if they did not correctly receive all doses to which they were randomized); secondary analysis may be conducted using as treated analyses (i.e. with patients grouped by the drug doses actually received).

5.4.3 Premature Termination or Suspension of Trial

At the end of each 67 patient dose cohort, grade II or higher AEs and any SAEs will be provided to

and reviewed by the DSMB+ and the PI, who will then consider whether the study can continue or whether it needs to be stopped

and/or whether other corrective action needs to take place. The external DSMB+ or the PI may also request additional interim analyses.

The study may be stopped if after any group of 67 patients the rate of grade III or higher SAEs (per CTCAE guidelines) in drug-treated patients is at least 10% and more than three times the rate of such events in the placebo-treated group. Considering the wide range of surgical procedures and patient comorbidities that could contribute to adverse events seen in this study, the DSMB+ should use both this quantitative cutoff and its clinical judgement in considering whether to stop the study. The study may be modified at any time by the sponsor, the FDA, or the external IRB as part of their duties to ensure that research subjects are protected.

6 STUDY AGENT

6.1 Study Agent and Control Description

6.1.1 Acquisition

CN-105 will be supplied by AegisCN and manufactured under contract by University of Iowa Pharmaceuticals, Iowa City IA. Study drug will be shipped directly from the manufacturer to the Duke pharmacy, whose investigational drug service will then dispense the drug at each participating study site (Duke Hospital, Duke Regional Hospital, Duke Raleigh Hospital).

6.1.2 Formulation, Appearance, Packaging, and Labeling

CN-105 will be supplied in amber glass vials containing 4 mL of a concentrated, 12.5-mg/mL, clear-to-slightly-yellow solution. The vials are stoppered and capped with over-seals. Cloudiness, change in color, and/or the presence of any particulate matter may indicate that the product has deteriorated; if any of these or other changes are noted, the affected vials will not be used, and the study team will communicate with AegisCN and the University of Iowa Pharmaceuticals as soon as possible.

Vials of CN-105 will have the following labeling information:

CN-105 12.5 mg/mL (50 mg/vial)
Store frozen at -70°C
Caution: New Drug—Limited by Federal (U.S.) Law to Investigational Use

Dosing labels will be attached to each dosing syringe/infusion bag. The individual participant syringe/infusion bag label will include both preprinted fields and handwritten fields as follows:

Protocol number
Personal identification number
Participant initials
Date
Time
Volume

6.1.3 Product Storage and Stability

If dosing solutions are prepared more than 1 hour before the planned start of the study drug infusion, the infusion bags containing the dosing solutions are to be refrigerated at 2 to 8°C in a secure and

locked refrigerator (and/or in a refrigerator in a secure and locked room). The drug can be refrigerated for up to 24 hours until approximately 60 minutes before the planned start of the infusion.

6.1.4 Preparation

Study drug will be diluted in normal saline by the Investigational Drug Service (IDS); placebo will be normal saline. The infusion will be administered using a 1.2-micron or smaller filter.

6.2 Dosage and Administration

We planned to enroll up to 250 patients total in this phase 2 study to obtain three successive dose groups of 67 patients each. In each group of 67 patients, 50 will receive a given dose of CN-105, and 17 will receive placebo. Due to a clerical error by the Investigational Drug Services that prepares our drug/placebo doses, 69 patients were randomized to the first patient cohort rather than 67.Because enrollment happens at consent, and not all patients that consent will be randomized, we will keep enrolling patients until we reach 67 consented and randomized patients in each cohort.

In each successive 67 patient group, participants will be randomized the week or day of surgery to receive either CN-105 (see Table 1 for dose levels) or placebo, within one hour before the scheduled or actual surgery start time and continuing every 6 hours until postoperative day 3 or hospital discharge (whichever occurs first), up to a maximum of 13 doses.

6.2.1 Route of Administration

Study drug or placebo will be administered intravenously over 5-10 min, up to maximum of 30 minutes.

6.2.2 Starting Dose and Escalation Schedule

Patients who receive CN-105 will receive one of three dose levels depending on their cohort: 0.1 mg/kg, 0.5 mg/kg, or 1 mg/kg, depending on whether they are in the first, second or third group of 67 patients, respectively. The 6 week +/- 3 week follow up visits for patients in the first two cohorts may be completed while the next cohort (higher dose level) is starting to be enrolled.

Table 1: Dose levels of CN-105

Cohort	Dose of CN-105	Patients receiving drug	Patients receiving placebo
Dose level 1	0.1 mg/kg	50	17
Dose level 2	0.5 mg/kg	50	17
Dose level 3	1 mg/kg	50	17

6.2.3 Dose Adjustments/Modifications/Delays

At the end of each 67-patient enrollment/ randomization group, grade II or higher AE rates and safety information will be provided to the DSMB. Upon evaluating this information, the DSMB plus will then consider whether the study should be stopped or whether other corrective action should take place. While grade II and/or higher AE and safety information from a given 67 patient randomized group is being analyzed and provided to the DSMB plus, and while the DSMB plus is

evaluating this data (as described above), the trial will continue with enrollment of the next 67 patient group (who will receive either placebo or the next higher dose of CN-105). Due to a clerical error by the Investigational Drug Services that prepares our drug/placebo doses, 69 patients were randomized to the first patient cohort rather than 67.

There is no data from either preclinical studies or our recent phase I study suggesting any dose-dependent toxicity or side effects from CN-105. Thus, there is no reason to halt the enrollment of a subsequent 67 patient group (which will include patients receiving a higher dose of CN-105) while the DSMB is awaiting and then analyzing data from the prior 67 patient group. Nonetheless, the DSMB plus will be empowered to make recommendations about whether the trial should be stopped or modified based on their evaluation of data in the prior 67 patient group(s).

6.2.4 Duration of Therapy

Study drug will be administered every 6 hours, with the first dose within one hour of planned or actual case start time, and subsequent drug doses will be given every 6 hours thereafter until postoperative day 3 or hospital discharge, whichever occurs first, up to a maximum of 13 doses. Each case start time will be defined by the study team as either the start of the surgery/procedure or the start of anesthesia (or the timed notes in the EMR for induction, intubation, or the start of anesthetic drug administration). If the case gets delayed by more than 6 hr and we've already given the first dose, we will give an additional dose prior to surgery, but under no conditions will the patient be given more than 13 doses total of CN-105

6.2.5 Tracking of Doses

Since the immediate pre- and post-operative periods are busy and nurses frequently have numerous tasks to perform, it is important to measure how many CN-105 (or placebo) doses are given as ordered to assess feasibility of q6 h dosing. We will track whether each CN-105 (or placebo) dose is given within +/-90 min of the scheduled administration time per nursing records.

We will report the observed rate of doses administered within the required time window and the 95% confidence interval. Each dose will be counted as an independent observation. The total number of doses correctly administered (i.e. within 90 min of the scheduled administration time) divided by the number of prescribed doses will define our dosing feasibility rate. Post-hoc feasibility analysis will be performed both among all study patients (including those receiving placebo), and specifically among CN-105 drug patients only, as well as by dose level to determine if drug dose level affects feasibility.

6.3 Study Agent Accountability Procedures

For each participant dose, details of study agent preparation and administration, i.e., time, date, dose level, volume, infusion time, etc., will be recorded in the source documents.

Upon receipt of CN-105 shipments, the pharmacist/designee will verify the condition of the vials and document per instructions in the Manual of Operational Procedures.

All used study agent vials, syringes, infusion sets, etc., will be kept in a secure location. After accountability is complete, used supplies may be discarded according to the site's normal procedures. All study vials (used/unused) remaining at the completion of the study will either be returned to the sponsor/designee or will be destroyed at the site. In case of the latter, the site will

maintain destruction records.

7 TRIAL PROCEDURES AND SCHEDULE

7.1 Trial-Specific Procedures

7.1.1 Initial Lumbar Puncture and Blood Draw

An LP will be performed prior to surgery (between the time of consent and the time of scheduled anesthesia/surgery start), with a standard spinal anesthesia kit containing a 25 gauge pencil-point needle (or other appropriate spinal needle per Good Clinical Practice) under strictly sterile conditions. The patient's back will be sprayed with benzocaine numbing spray and we will wait approximately 10 minutes for analgesic relief prior to performing the lumbar puncture. This preoperative LP will be performed by an anesthesiology attending physician or senior anesthesiology resident or fellow under attending supervision, or a nurse practitioner or physician assistant under appropriate supervision.

The LP will be performed using a standard spinal anesthesia kit per the manufacturer's directions under sterile conditions at the L4–5 spinal level or the next interspace above or below this level, depending on which interspace appears larger by palpation. A curvilinear ultrasound probe will be used when necessary to help visualize the interspace. An initial 1 ml of CSF will be discarded (to reduce blood contamination of the CSF) before collecting a study sample of up to 20 ml of CSF. Blood (up to 10 mL) will be collected via sterile venipuncture, from an arterial line, or by the IV line placed before surgery. To assess their level of pain following the lumbar puncture procedure, subjects will be asked to answer the following question: How much did the lumbar puncture hurt (on a scale of 0-10, where 0 is no pain at all and 10 is the worst pain imaginable?'

LP vital signs will be collected and recorded by the study team before and after the LP if performed on outpatient basis (between the time of consent and surgery day).
If the LP is performed while inpatient (i.e. on the day of surgery, or at 24 hrs after surgery), the study team will not collect and record pre and post LP vital signs, as these will already be recorded in the patient's hospital electronic medical record per standard of care.

7.1.2 Postoperative Lumbar Puncture and Blood Draws

LPs at 24 hours and at 6 weeks +/- 3 weeks after the start of anesthesia/surgery will be performed by an anesthesiology attending physician or senior anesthesiology resident or fellow under attending supervision, or a nurse practitioner or physician assistant under appropriate supervision. To assess their level of pain following each lumbar puncture procedure, subjects will be asked to answer the following question: How much did the lumbar puncture hurt (on a scale of 0-10, where 0 is no pain at all and 10 is the worst pain imaginable?'

If the participant is hospitalized overnight after the surgery, the 24-hour postoperative LP may be performed in the participant's hospital room, in the preoperative holding area, or in other appropriate space for performing a sterile neuraxial procedure per Good Clinical Practice (GCP) guidelines. If the participant is unexpectedly discharged after surgery on the same day and does not spend the first night in the hospital, the patient will be asked to return for these procedures, and then the 24-hour postoperative LP will be performed in the same location as the 6 week +/- 3 weeks postoperative LPs are performed or in another safe location for performing sterile neuraxial anesthetic procedures per GCP guidelines. As for the preoperative LP, an initial 1 ml of CSF will be

discarded (to reduce blood contamination of the CSF) before collecting up to a 20ml CSF sample for the study.

The 6 week +/- 3 week postoperative LPs will also be performed in the preoperative or postoperative holding area, or in other clinical research space of the Duke Anesthesiology department that is appropriately outfitted for human subject procedures (i.e. presence of medical gases, suction, resuscitation equipment. As with the preop and 24 hr postop LPs, an initial 1 ml of CSF will be discarded (to reduce blood contamination of the CSF) before collecting up to a 20ml CSF sample for the study.

The 24 hr and 6 week +/3 week postoperative LP vital signs will be collected and recorded by the study team before and after the LP if performed on outpatient basis. If the LP is performed while the patient is an inpatient, the study team will not collect and record vital signs, as these will already be obtained by the patient's clinical care team and recorded in the patient's hospital record per standard of care.

The LP will then be performed at the L4-5 interspace or the interspace above or below this level, depending on which space feels more open by palpation and/or appears more open by ultrasound visualization. The LP will be performed using a spinal anesthesia kit per the manufacturer's directions under sterile conditions. The participant may be continuously monitored for 15 minutes or longer if deemed necessary.

Blood will be drawn by sterile venipuncture, or from an arterial or an IV line if already in place, at the same time as the lumbar punctures.

7.1.3 Neurocognitive Testing, Delirium Testing, and Other Assessments

Neurocognitive testing and assessments may be performed remotely via teleconference or may be performed in-person in one of the study testing offices located in Duke South or other appropriate location preoperatively (baseline) and/or at 6 weeks +/- 3 weeks after surgery. They will also occur for any patients who have a 1 year post-operative study visit. Tests from the following standardized tests and assessments list will be utilized, which have equivalent alternate forms (to minimize test-retest practice effects) and have English, French and Spanish language versions:

a. Hopkins Verbal Learning Test, Revised (HVLT-R) - an auditory-verbal word-list task used to assess learning, short-term recall, long-term recall and recognition memory that is co-normed with the BVMT-R (see immediately below).
b. Brief Visuospatial Memory Test, Revised (BVMT-R) – a visual measure of constructional praxis, spatial/figure learning, short-term recall, long-term recall and recognition memory that is co-normed with the HVLT-R (see immediately above).
c. Digit Span subtest from the Wechsler Adult Intelligence Scale, 3rd Revision (WAIS-III) – a test of immediate auditory-verbal recall and complex attention (i.e., working memory).
d. Digit Symbol Coding subtest from the Wechsler Adult Intelligence Scale, 3rd Revision (WAIS-III) - a matching-to-sample visual scanning and visuomotor production task.
e. Trail Making Test (TMT), Parts A & B – a test of simple visuomotor scanning and complex sequencing and set-switching executive demands.
f. Controlled Oral Word Association Test (COWAT) – a test of lexical verbal fluency and information retrieval efficiency.
g. Grooved Pegboard Test – a timed test of bilateral manual dexterity and motor speed.
h. Wechsler Test of Adult Reading (WTAR) – a non-phonemic reading test used to assess crystalized pronunciation knowledge; a reliable estimate of premorbid intellectual

functioning.

i. MoCA: used to screen for cognitive impairment.

j. STOP-Bang brief questionnaire: to assess sleep apnea risk during one of the participants' trial visits.

k. Visual analogue scale (VAS) pain assessment at each trial visit (preoperative, 6 weeks +/- 3 weeks postop.

Other assessments performed at both the preoperative and 6 week (+/- 3 week) postop visits may include:

a. List of medications participants are currently taking at each trial visit.

b. Timed up and Go task/test, Romberg assessment, and falls assessments. In particular, patients will undergo a brief falls risk assessment questionnaire, which contains questions from the Elderly Falls Screening test (Cwikel J et al, Disabil Rehab, 1998) and the Fall Risk Screening test (Tromp et al, J Clin Epidemiology, 2001), and questions about whether they have fallen recently.

Relevant tests listed above may be recorded using a digital voice recorder for quality assurance purposes. Quality assurance activities will include reviewing of testing audio files, generating performance review documents, and providing feedback on accuracy and completeness. There is no risk to the trial participants since participant identifiers will not be provided. Trial participants will not be identified by name on the recording. Only the trial number, test date, and initials of the tester will be provided on the digital file label. The link between trial number and participant identifiers will be maintained. These files will be stored on locked computers within locked offices to which only authorized research personnel will have access. These files will be destroyed after the primary outcome analysis of this research trial is complete.

Delirium Testing will be performed twice daily starting on the day of surgery until postoperative day 5 or hospital discharge (whichever occurs first), as well as at the baseline study visit and the 6 week +/- 3 weeks postoperative study visit, as well as the 1 year visit that some patients may have, using the CAM-ICU instrument in intubated patients (Ely EW et al, 2001) and the 3D-CAM instrument in non-intubated patients (Marcantonio E et al, 2014), based on the enhanced sensitivity of these instruments in these respective patient populations (Kuczmarska A et al, 2016). On the day of surgery, delirium testing will be performed once while the patient is in the post-anesthesia care unit (PACU) after surgery and once later in the afternoon or early evening on the day of surgery, unless the PACU delirium assessment is performed after 4:00 PM. In that case, no additional afternoon/evening testing assessment will be performed on that day. If the surgery ends late and if the PACU delirium assessment cannot be performed before 7:00 PM, then no delirium assessments will be performed on the day of surgery. Inter-rater reliability for these assessments will be assessed as previously described (Marcantonio E et al, 2014).

7.1.4 Quality of Life Assessment

Our approach to measuring QOL for this trial involves the use of a battery of well-tested and well-validated instruments that together cover the relevant domains of interest. For this trial, we propose to use an assessment instrument that the Randt group developed, the SF-36 generic QOL instrument, from their longer instruments used in the Health Insurance Experiment and in the Medical Outcomes Study, as well as functional status measures (the Duke Activity Status Index [DASI]); psychological measures, including depression (the Center for Epidemiological Studies- Depression [CES-D]), the State Trait Anxiety Inventory [STAI], and the Hopkins Symptom Checklist [HSCL] (SCL-90); and

social/role functioning (including employment status).

Participants will complete these questionnaires during the baseline trial visit after completing all consent paperwork and again at the 6 week +/- 3 weeks postoperative visit. Any participant who is unable to complete this questionnaire at either trial visit will be given an e-copy or a paper copy of the questionnaire to fill out at home and to mail back to our study team at his or her convenience (in the case of paper questionnaires). Mail in questionnaires will be accepted from patients as long as they are completed by the patient within +/- 3 weeks of the preoperative study visit and before the surgery, or if they are completed within +/- 3weeks of the 6 week +/- 3 week postoperative study visit.

7.1.5 Electroencephalographic Recording

Scalp recordings of electrical activity produced by the brain (EEGs) will be obtained for 3-60 minutes before the start of anesthesia, as well as during and right after anesthesia and surgery. The EEG will be recorded from 32 electrodes embedded in an elastic scalp electrocap, using a state-of-the-art, battery-operated, portable EEG system (Live-amps, Brain Vision LLC, Morrisville, NC). If necessary due to time constraints or other considerations, the study team may also use a BIS electrode strip to monitor the patient's brain waves instead of the full 32 channel EEG cap setup. The electroencephalographic data will be analyzed to determine the effect of CN-105 on EEG correlates of postoperative cognitive function and post-operative delirium, as well as to identify possible novel pre- and intraoperative EEG correlates of POCD, delirium, and/or neuroinflammation (with the latter as measured by CSF inflammatory cytokine levels at 24 hours after anesthesia and surgery). In the event that sufficient personnel or equipment are unavailable, or in the event that two MARBLE patients are having surgery at the same time, or in the event that there is insufficient time to place the EEG sensor on that patient's head before the start of surgery, the study team may not record EEG from all patients or at all points before, during, and just after surgery/anesthesia

In addition to passive EEG recordings, patients may also undergo auditory event related potential testing during EEG recording in which brief sounds will be administered via earphones during the pre-operative and/or intra-operative EEG recordings, when the pre-op and surgical workflow allows and when study personnel are available. The volume of these brief intermittently played sounds would be well within the range of normal daily exposures, i.e., <= 80 dB, a level that would be safe for even a full day of continuous exposure (MRC Institute of Hearing Research, 1996), which is much more than is proposed here. Patients may further undergo brief audiometric screening in which sounds are delivered via headphones to ensure that they are able to hear the presented sounds at a volume of 80 dB or less without auditory assist devices (i.e., without hearing aids, cochlear implants, or BAHA devices). This would not require any additional visits. EEG responses to auditory event-related potentials have been associated with neurocognitive reserve previously (Speer and Soldan, 2015). Collecting this data may increase the team's ability to identify EEG-based biomarkers of perioperative neurocognitive resilience.

7.2 Standard-of-Care Trial Procedures

7.2.1 Anesthetic Treatment

All participants will receive either general anesthesia or sedation (i.e. monitored anesthesia care); patients may also receive regional or neuraxial nerve blocks as adjuncts.

The duration of anesthetic exposure shall be recorded for each participant, and intraoperative anesthetic medication administration and physiologic monitoring data will be extracted from the electronic anesthetic records for post-hoc analyses.

7.3 Laboratory Procedures/Evaluations

7.3.1 Clinical Laboratory Evaluations

A basic metabolic panel (i.e. Na^+, K^+, Cl^-, CO^2, BUN, Cr and glucose) and a complete blood count (with differential) will be measured at three time points: within 2 weeks before anesthesia and surgery (and before the first dose of CN-105), 1 day after the start of anesthesia/surgery (i.e., after ~4 doses of CN-105), and at the 6 week +/- 3 weeks postoperative study visit.

7.3.2 Other Assays or Procedures

We will measure CN-105 levels in both the serum and CSF at the 24-hour postoperative time point. This will help us determine how the CN-105 doses administered to patients in this study translate into serum drug concentrations, and will help us determine the extent of CN-105 penetration in the human CNS (as assayed in the CSF). We note that we do not plan to perform these measurements at the preoperative time point (as there should be no CN-105 in the serum or blood before the first dose is administered), or at the 6 week +/- 3 weeks postoperative study appointment (as the last CN-105 dose will be given no later than on postoperative day 3, and thus CN-105 levels would be expected to be undetectable ~6 weeks later).

We will perform serum and CSF CN-105 measurements together with our colleagues at MPI research (Mattawan, MI; now part of Charles River Laboratories), with whom AegisCN has previously performed these measurements. In brief, CN-105 will be detected via a previously validated high-performance liquid chromatography-tandem mass spectrometry assay (LC-MS/MS). The liquid chromatography system uses an ACE 3 C8 column, 2.1 x 50 mm (3 µm particle size) with an isocratic flow consisting of water/acetonitrile/heptafluorobutyric acid (76/24/0.05, v/v/v) at a flow rate of 200 µL/minute (Agilent 1200 Binary Pump) and a post column infusion consisting of isopropanol/acetic acid (100/1, v/v) at a flow rate of 800 µL/minute (Agilent 1100 LC Quaternary Pump). The analyte and internal standard (acebutolol hydrochloride) are detected using a SCIEX API 5000 triple quadrupole LC-MS/MS system equipped with an ESI (TurboIonSpray®) ionization source operated in the positive ion mode. Multiple reaction monitoring transitions of the respective $[M+H]^+$ ions are used to monitor CN-105 and acebutolol hydrochloride.

7.3.3 Specimen Preparation, Handling, and Storage

Blood samples will be used for genotyping and metabolic/inflammatory marker assays. Whole-blood specimens (10 mL total volume) will be collected sometime between the patient being consented and scheduled anesthesia/surgery start time, 24 +/- 2 hours later, and 6 weeks +/- 3 weeks later.

Whole-blood specimens will be collected with EDTA as the anticoagulant, obtained either from an arterial or IV catheter or from sterile venipuncture.

Cerebrospinal fluid specimens will be analyzed for amyloid beta, tau, and other related AD-associated neural markers and inflammatory cytokines. Cerebrospinal fluid specimens will be collected when the LP is performed before surgery (i.e. between the time of consent and anesthesia/surgery start), 24 hours after the start of anesthesia/surgery, and at the 6 week +/- 3 weeks follow up visit.

Cerebrospinal fluid and blood samples will be collected by a trained care provider under sterile

conditions. Blood and CSF samples will be stored at -80°C. All assay measurements will be performed at DUMC or in the accredited laboratory of a collaborator. All CSF and blood samples will be held in freezers in the Department of Anesthesiology and/or DUMC research facilities.

7.3.4 Specimen Shipment

Relevant specimens will be shipped on dry ice by FedEx overnight express to relevant collaborating labs for measurements, such as to MPI/Charles River and to the ADNI biomarker core lab at the University of Pennsylvania or other appropriate laboratory for tau, phospho-tau, and A® measurements.

7.4 Trial Schedule

7.4.1 Initial Consent and Visit

The following procedures and assessments will be conducted at the time of initial study consent or between the Initial study consent and the day of surgery:

Informed consent

Enrollment

Demographics (age, sex, and race)

Medical history

Neurocognitive testing will be done either in-person or remotely via teleconference (may include WTAR; HVLT-R; BVMT-R; WAIS-III —Digit Span, Digit Symbol Coding subtests, TrailTMT, Parts A & B, COWAT and Grooved Pegboard; MoCA) as well as the STOPBang assessment, VAS pain assessment, Falls assessments, Timed Up and Go Test, and Romberg Test

DASI

Psychological measures (including CES-D, STAI, SCL-90, SF-36)

Record concomitant medications administered per standard of care

Delirium Screening with the 3D-CAM instrument

Collect CSF sample/ LP prior to surgery (i.e. between the time of consent and the time of scheduled anesthesia/surgery start, which means it could be done at the initial visit or anytime between the time of consent at the initial visit and the time of scheduled anesthesia/surgery start on the day of surgery)

Collect research blood sample prior to surgery (i.e. between the time of consent and the time of scheduled anesthesia/surgery start, which means it could be done at the initial visit or anytime between the time of consent at the initial visit and the time of scheduled anesthesia/surgery start on the day of surgery)

LP vital signs will be collected and recorded by the study team before and after the LP if performed on outpatient basis (between

the time of consent and surgery day).

If the LP is performed while the patient is an inpatient (i.e. on the day of surgery), the study team will not collect and record vital signs, as these will already be obtained by the patient's clinical care team and recorded in the hospital electronic medical record for the patient, per standard of care.

-Randomization assignments will be made within 1 week prior to the patient's scheduled surgery.

7.4.2 Day 0 (Day of Surgery)

The following procedures and assessments will be conducted at this visit:

Collect CSF sample/ LP prior to surgery (if not done at the time of the Initial Consent and Visit)

Concomitant medications administered per standard of care.

Collect blood sample (if not done at the time of the Initial Consent and Visit)

Laboratory assessments

Study drug or placebo administration (within one hour prior to scheduled or actual surgery start time, and subsequent drug doses will be given every 6 hours thereafter until 3 days after surgery or hospital discharge, whichever comes first, up to a maximum of 13 doses) by the patient's clinical care team. Each case start time will be defined by the study team as either the start of the surgery/procedure or the start of anesthesia. If the case gets delayed by more than 6 hr and we've already given the first dose, we will give an additional dose prior to surgery, but under no conditions will the patient be given more than 13 doses total of CN-105.

EEG (within 2 hours before surgery as well as during and just aftersurgery). In the event that sufficient personnel or equipment are unavailable, or in the event that two MARBLE patients are having surgery at the same time, or in the event that there is insufficient time to place the EEG sensor on that patient's head before the start of surgery, the study team may not record EEG from all patients or at all points before, during, and just after surgery/anesthesia. Auditory tones will be played and auditory event-related potentials will be recorded if the pre-op and surgical workflow allows and if study personnel are available.

Vital signs (including heart rate) will be obtained and entered into the anesthetic record during the surgery by the clinical care team as per GCP, standard of care, and the American Society of Anesthesiology guidelines .

Continuous heart rate monitoring will be obtained for up to 15 minutes prior to surgery and during the surgery, to assess heart rate variability (R-R interval) as an index of vagal nerve function.

Grade II or higher AEs (and all SAEs) will be monitored and recorded from just after the first study drug or placebo administration until the end of the 6 week +/- 3 weeks postoperative study follow up visit.

Delirium Screening may be administered with 3D-CAM or CAM-ICU up to two times after surgery.

7.4.3 Day 1 (First Day after Surgery, Hospitalization)

The following procedures and assessments will be conducted at this visit:

Collect CSF sample/ LP

LP vital signs will be collected and recorded by the study team before and after the LP only if the LP is performed on an outpatient basis.

Concomitant medications will be administered according to standard of care

Collect blood sample

Laboratory assessments

Study drug or placebo administration (every 6 hours until 3 days after surgery or hospital discharge, whichever comes first, up to 13 doses maximum) by the patient's clinical care team

Grade II or higher AEs recorded

Delirium Screening will be administered with 3D-CAM or CAM-ICU

7.4.4 Days 2-4 (Hospitalization)

The following procedures and assessments will be conducted at this visit:

Concomitant medications will be administered according to standard of care

Study drug or placebo administration (every 6 hours until the 3rd day after surgery or hospital discharge, whichever comes first, for a maximum of 13 doses) given by the patient's clinical care team.

Grade II or higher AEs recorded

Delirium Screening will be administered with 3D-CAM or CAM-ICU until postoperative day 5 or hospital discharge (whichever occurs first).

7.4.5 6 weeks (+/- 3 weeks) Follow-Up

The following procedures and assessments will be conducted at this visit:

Neurocognitive testing will be done either in-person or remotely via teleconference (may include HVLT-R; BVMT-R; WAIS-III —Digit Span, Digit Symbol Coding subtests, TMT Parts A & B, COWAT, MoCA, and Grooved Pegboard; STOPBang (only if it wasn't performed at the preoperative visit) VAS pain assessment, Falls assessments, Timed Up and Go Test, Romberg Test)

Laboratory assessments

DASI

Psychological measures (including CES-D, STAI, Hopkins SCL-90)

SF-36

Collect CSF samples / LP

Vital signs will be collected and recorded by the study team before and after the LP, only

if the patient is not already hospitalized as an inpatient at this time.

Collect blood samples

Grade II or higher AEs recorded

Delirium assessment (using either the 3D-CAM instrument, or the CAM-ICU instrument in the unlikely case that the patient is intubated at the time of this study visit).

STOP-Bang sleep apnea screening assessment may be performed, if it was not already completed at the baseline study visit or at another point during the study.

7.4.6 Schedule of Events

Table 2: Schedule of Study Events for Trial

	Screening/ Baseline Study Visit	Within 1 week before surgery	Day 0 Surgery	Day 1 [a] Hospitalization	Days 2-4 Hospitalization	6 weeks (+/- 21 days) Follow-up
Informed consent	X					
Enrollment	X					
Randomization		X				
Demographics (age, sex, and race)	X					
Vital signs			X [b, e]	X [b]		X [b]
Medical history	X					
Neurocognitive testing [c]	X					X
Continuous Heart Rate Recording			X			
Laboratory assessments (additional routine clinical pre- + post-op labs will also be recorded)			X	X		X
Duke Activity Status Index	X					X
Psychological/physical measures [d]	X					X
Short Form-36	X					X
Lumbar puncture, Collect CSF samples			X [e]	X		X
Concomitant medications	X		X	X	X	X
Collect blood sample			X [f]	X		X
Drug administration [g]			X	X	X	
Electroencephalogram (EEG)			X [h]			
Grade II or higher AEs/ all serious AEs			X	X	X	X
3D-CAM or CAM-ICU for delirium screening	X		X	X	X [i]	X
Biomarker assays			X	X		X
Genotyping assays			X [f]			
Inflammatory protein analysis			X	X		X
CSF protein analysis			X	X		X

AE, adverse event; CSF, cerebrospinal fluid;
QOL, quality of life

[a] 24 (+/- 2) hours post-surgery.
[b] LP vital signs will be collected and recorded by the study team before and after the LP only if it is performed on an outpatient basis.
[c] May include Hopkins Verbal Learning Test-Revised; BVMT-R ; Wechsler Adult Intelligence Scale-III—Digit Span, Digit Symbol subtests; Wechsler Test of Adult Reading; Trail Making Test Parts A & B; Grooved Pegboard; Montreal Cognitive Assessment (MoCA); STOPBang Questionnaire; visual analog scale pain assessment;
[d] May include Center for Epidemiological Studies-Depression, State Trait Anxiety Inventory, Hopkins Symptom Checklist, Falls assessments, Timed Up and Go, and Romberg Assessments.
[e] Between the time of consent and the scheduled anesthesia/surgery start time on the day of surgery.
[f] Between the time of consent and the scheduled anesthesia/surgery start time on the day of surgery.
[g] w/in 1 hr before the scheduled or actual start time of surgery and continuing every 6 hours through postoperative day 3 (up to 13 doses maximum) or hospital discharge, whichever occurs first
[h] Immediately before , during, and after surgery/anesthesia. In the event that sufficient personnel or equipment are unavailable, or in the event that two MARBLE patients are having surgery at the same time, or in the event that there is insufficient time to place the EEG sensor on that patient's head before the start of surgery, the study team may not record EEG from all patients or at all points before, during, and just after surgery/anesthesia. Auditory tones will be played, and auditory event-related potentials will be recorded if the pre-op and surgical workflow allows and if study personnel are available.
[I] This will be administered until postoperative day 5 or hospital discharge (whichever occurs first).

8 ASSESSMENT OF SAFETY

8.1 Specification of Safety Parameters

8.1.1 Definition of Adverse Events (AEs)

An AE is any untoward medical occurrence associated with the use of a drug in a participant, whether or not considered drug related. An AE can, therefore, be any unfavorable and unintended sign, including an abnormal laboratory finding, for example, symptom, or disease temporally associated with the use of a pharmaceutical product. Diseases, signs, symptoms, or laboratory abnormalities already existing at randomization are not considered AEs unless they worsen (i.e., increase in intensity or frequency). Surgical procedures planned before randomization and the conditions leading to the need for these surgical procedures are not AEs.

8.1.2 Definition of Serious Adverse Events

An AE is considered serious if any of the following occur or the investigator believes any of the following outcomes may occur:

1. Death.
2. Life-threatening AE: places the participant, in the view of the investigator, at immediate risk of death at the time of the event as it occurred. It does not include an AE that, had it occurred in a more severe form, might have caused death.
3. Persistent or significant disability or incapacity or substantial disruption of the ability to conduct normal life functions.
4. Congenital abnormality or birth defect.
5. Requires inpatient hospitalization or prolongation of existing hospitalization with the following exceptions:

    Preplanned (before the trial) hospital admissions, unless the hospitalization is prolonged

    Planned admissions (as part of a trial, e.g. routine biopsies)

    Re-hospitalizations (which occur less than 24 hours after discharge from index hospitalization)

    Elective procedures

    Emergency room visits

6. Important medical events that may not result in death, be life threatening, or require inpatient hospitalization may be considered a serious AE (SAE) when, based on appropriate medical judgment, they may jeopardize the participant and may require medical or surgical intervention to prevent one of the serious outcomes listed above. This determination is based on the opinion of the investigator.
7. SAEs that are thought to be related (i.e. more likely than not to be related) to the study drug will be reported to the FDA and all other appropriate regulatory bodies within 15 days of the study time documenting the SAE. SAEs thought not to related to the study drug will reported on a yearly basis.

8.1.3 Definition of Unanticipated Problems

Unanticipated problems involving risks to participants or others include, in general, any incident, experience, or outcome that meets all of the following criteria:

Unexpected in terms of nature, severity, or frequency given (a) the research procedures that are described in the protocol-related documents, such as the IRB-approved research protocol and informed consent document; and (b) the characteristics of the participant population being studied

Related or possibly related to participation in the research ("possibly related" means there is a reasonable possibility that the incident, experience, or outcome may have been caused by the procedures involved in the research)

Suggests that the research places participants or others at a greater risk of harm (including physical, psychological, economic, or social harm) than was previously known or recognized

8.2 Classification of an Adverse Event

8.2.1 Severity of Event

Assessment of severity of an event will be based on the following:

Mild: Is generally transient, does not interfere with usual activities, and is easily tolerated by the participant.

Moderate: Causes sufficient discomfort and interferes with usual activities.

Severe: Causes considerable interference with usual activities and may be incapacitating.

Whenever appropriate and/or possible, we will define AE's by the NIH Common Terminology Criteria for Adverse Events (available online at https://ctep.cancer.gov/protocoldevelopment/electronic_applications/docs/CTCAE_v5_Quick_Reference_5x7.pdf

Multiple grade I (i.e. mild) AEs per CTCAE criterion typically occur after major non-cardiac surgery in older adults (i.e. our study population). Examples of such grade I AEs that occur in virtually every older adult after major non-cardiac surgery include hemoglobin declines to between 10 mg/dL and the lower limit of the normal range, serum Ca++ drops to between 8.0 and the lower limit of the normal range, WBC count increases to the 10-20 range, etc. Thus, to avoid capturing a massive amount of such grade I AEs that are unlikely to be related to study drug administration, here we will capture and report just those AEs of grade II or higher, and all SAEs.

8.2.2 Relationship of Study Agent

Assessment of the relationship of an event to the study agent will be based on the following:

Unrelated = None; Remote

- None (unrelated, not related, no relation)
  - Unreasonable temporal relationship between administration of the study agent and the onset of the AE (e.g., the event occurred either before, or too long after, administration of the study agent for it to be considered drug related)
  - Causal relationship between the study agent and the AE is biologically implausible (e.g., death as a passenger in an automobile accident)
  - Clearly more likely alternative explanation for the AE is present (e.g., typical adverse reaction to a concomitant drug and/or typical disease-

related event)

- Remote (unlikely, doubtful, improbable)
  Little evidence to suggest there is a causal relationship and there is another reasonable explanation for the event.

Related = Possible, Probable

- Possible
  - There is some evidence to suggest a causal relationship (e.g., the event occurred within a reasonable time frame after administration of the study agent); however, the influence of other factors may have contributed to the event (e.g., the participant's clinical condition, other concomitant medications, or concurrent illness)
- Probable
  - There is evidence to suggest a causal relationship between the event and the study agent, and the influence of other factors is unlikely.

The investigator should consider the following when assessing causality:

Temporal associations between the study agent and the event

Pre-existing risk factors or concurrent illness

Plausible mechanism

8.2.3 Expectedness

The investigator will be responsible for determining whether a grade II or higher AE is expected or unexpected. A grade II or higher AE will be considered unexpected if the nature, severity, or frequency of the event is not consistent with the risk information previously described for the study agent in the protocol.

8.3 Time Period and Frequency for Event Assessment and Follow-up

The investigator will record all reportable events starting from the time just after the first administration of study drug (or placebo) until the last trial-related procedure/visit. At each trial visit, the investigator or study team will inquire about the occurrence of grade II or higher AE and any SAEs since the last visit. Events will be followed for outcome information until resolution or stabilization. Adverse events of grade II or higher for participants who discontinue trial participation will be collected/reported through at least the time of discontinuation.

When additional relevant information (final diagnosis, outcome, results of specific investigations, etc.) becomes available, the investigator will submit this additional information to the DSMB at the time of the next DSMB report (or earlier, if deemed appropriate by the PI).

8.4 Reporting Procedures

All grade II or higher AEs that occur between the time immediately following the first administration of study drug (or placebo) until the last trial-related procedure/visit will be recorded. Adverse events may be volunteered by subjects and/or solicited at each assessment by the investigator or designee.

Recording will be performed in a concise manner using standard, acceptable medical terms. The research team (and the Principal Investigator) will make every effort to establish a diagnosis of the event based on the presenting signs, symptoms, and/or clinical information. In such cases, the diagnosis will be documented as an AE/SAE.

Diseases, signs, symptoms, or laboratory abnormalities already existing at enrollment will not considered AEs unless they worsen (i.e., increase in intensity or frequency). Surgical procedures planned before enrollment and the conditions leading to these measures are not AEs, and the normal post-operative recovery course of a surgical procedure planned before enrollment also will not constitute an AE. Thus, for example, if a study patient has a planned knee replacement surgery before he or she enrolls in the study, the patient has this surgery, and a postoperative knee x-ray shows the normal postoperative presence of the replacement knee, this will not be considered an AE.

If, in the investigator's judgment, a clinically significant worsening from baseline is observed in any laboratory or other test parameter (e.g., ECG), physical examination finding, or vital sign, a corresponding clinical AE should be recorded. If the cause is not known, the abnormal test or finding should be recorded as an AE, using appropriate medical terminology (e.g., thrombocytopenia, peripheral edema, QT prolongation). If this AE is of grade II or higher per CTCAE criteria, this event will be recorded and reported to the DSMB+.

8.4.1 Serious Adverse Event Reporting

8.4.1.1 Reporting to the DSMB and IRB

All dru-related SAEs (i.e. if thought to be drug- related, or more likely than not to be related to the drug), occurring between between the time immediately following the first administration of study drug (or placebo) until the last trial-related procedure/visit will be reported by the investigator or qualified designee within 5 working day of first becoming aware of the event to the IRB and DSMB. For SAEs deemed "probably" drug-related, reporting to the IRB and/or FDA will occur within appropriate time windows, per institutional and FDA requirements.

8.4.1.2 Reporting to Regulatory Authorities

All SAEs will be reviewed for expectedness by the Principal Investigator or another investigator designated by the Principal Investigator. SAEs determined to be "probably" related to the investigational drug and that are unexpected according to the protocol will be reported in an expedited manner to the FDA, in accordance with 21 CFR 312.32. For unexpected drug-related SAEs that result in death or are considered life threatening, they will be reported to the FDA as soon as possible but in no case later than 7 calendar days after the sponsor's initial receipt of the information. For all other unexpected, drug-related SAEs, reporting to the FDA will occur no later than 15 days after the event. Non drug-related SAEs will be reported to the FDA on a yearly basis.

8.5 Trial Halting Rules

At the end of each 67 patient dose cohort, grade II or higher AEs (and all SAEs) will be provided to and reviewed by the DSMB, which will then consider whether the study can continue or whether it needs to be stopped and/or other corrective action needs to take place. Due to a clerical error by the Investigational Drug Services that prepares our drug/placebo doses, 69 patients were randomized to the first patient cohort rather than 67. Additionally, because enrollment happens at consent, and not all patients that consent will be randomized, we will keep enrolling patients until we reach 67 consented and randomized patients in each of the last two cohorts.

8.6 Safety Oversight

A DSMB composed of clinical/translational neuroscience investigators from outside of Duke (i.e. a DSMB+) will be convened before trial initiation and will evaluate the incidence of AEs in the placebo vs CN-105 treatment groups at the completion of each 67 patient cohort, as enrollment for the next 67 patient study cohort (and the next successive higher dose of CN-105) is beginning.

The DSMB+ will be empowered to halt the study during enrollment of the next 67 patient cohort if the following two conditions are met during the DSMB+ review of the prior 67 patient study cohort. First, that there is an apparent, consistent and persistent evidence of net harm (i.e. grade II or higher AE's, or SAEs, that occur at significantly increased frequency in patients who receive CN-105 vs placebo and which are viewed as likely resulting from CN-105 treatment rather than other factors or chance), and second that this net harm is perceived to overwhelm any benefit from study drug treatment and the overall knowledge to be obtained from the study. In this way, participants can be prevented from receiving higher doses of CN-105 if we determine that lower doses are consistently associated with an increased incidence of grade II or higher AEs.

9 CLINICAL MONITORING

Clinical site monitoring will be conducted by the on-site study team to ensure that the rights and well-being of human subjects are protected, that the reported trial data are accurate, complete, and verifiable, and that the conduct of the trial is in compliance with the currently approved protocol/amendments, with GCP, and with applicable regulatory requirements. The study team will perform this monitoring at least on a quarterly basis.

10 STATISTICAL CONSIDERATIONS

10.1 Description of Statistical Methods and Analysis Plans

10.1.1 General Approach

Data will be analyzed using standard analysis programs, including SAS and SPSS. R may also be used if necessary.

10.1.2 Safety and Feasibility Endpoints

Safety Analysis: Our DSMB will examine all grade II or higher AEs and all SAEs in CN-105 and placebo treated patients. The expected rate of moderate and severe adverse events in this patient population is 5% each, based on historical Duke orthopedic surgery data; thus we expect similar AE rates in this trial in placebo- treated patients. At the end of each 67 patient randomized group, the DSMB will examine grade II or higher AE rates in drug-treated vs placebo-treated patients, and consider whether the study should be stopped or whether other corrective action should take place. An independent statistician will prepare summary reports (including all grade II or higher AE rates in drug vs placebo treated patients) for review by the DSMB+ at the end of each 67 patient randomized group. Thus, the study team (and the primary study statistician) will thus remain blinded to patient treatment assignment throughout the course of the study. Due to a clerical error by the Investigational Drug Services that prepares our drug/placebo doses, 69 patients were randomized to the first patient cohort rather than 67. Additionally, because enrollment happens at consent, and not all patients that consent will be randomized, we will keep enrolling patients until we reach 67 consented and randomized patients in each of the last two cohorts.

Assuming that we continue with all three dose groups, at study completion we will have ≥117 total CN-105 treated patients and ≥39 placebo-treated patients. Given the aforementioned expected grade II or higher AE rates in placebo-treated patients, at completion we will have >80% power (with a=0.05) in a two- sample two-sided unpooled variance chi-square test to detect an absolute difference of 14.8% between grade II or higher AE rates seen in placebo vs CN-105 treated patients. We will also track the rates of other grade II or higher AEs recorded by the patient's surgical team in the medical record; at study completion we will use 2 sided t tests to compare the rates of any other grade II or higher AEs between placebo vs CN-105 treated patients.

Feasibility Analysis: We will report the observed rate of doses administered within the required time window and the 95% confidence interval. Each dose will be counted as an independent observation. The total number of doses correctly administered (i.e. within 90 min of the scheduled administration time) divided by the number of prescribed doses will define our dosing feasibility rate. Post-hoc feasibility analysis will be performed among all patients, as well as specifically in patients receiving the study drug only, and by dose level to determine if the drug itself or drug dose level affects feasibility.

10.1.3 Analysis of the Secondary Endpoints

CSF Inflammatory Cytokine Analysis: Non-parametric 2-sided tests will be used to detect differences in CSF inflammatory cytokine increases from before surgery to 24 h after surgery in CN-105 vs placebo treated patients. For our primary analysis, we will group all three CN-105 drug dose groups together into one larger group of ≥117 CN-105 treated patients. In unpublished data from the MADCO-PC study, we observed a pre- to 24 h post-op CSF IL-6 change of 21.55 pg/ml (SD=44.72; log normal distribution; see Supplement Appendix Figure 2). Markov-chain Monte Carlo simulations based on these data demonstrate that this study (with ≥ 117 CN-105 treated and ≥ 39 placebo treated patients) will have >80% power (with 〈=0.05) in a 2-sided Wilcoxon Rank Sum test to detect a 34% smaller increase in IL-6 levels from before to 24 h after surgery in CN-105 vs placebo treated patients. Based on our preliminary MADCO-PC data, this sample size will also provide >80% power to detect a 36% smaller increase in G-CSF, a 32% smaller decrease in IL-8, and a 44% smaller decrease in MCP-1 levels from before surgery to 24 h after surgery in CN-105 vs placebo-treated patients. A post-hoc Kruskal-Wallis analysis will be used to compare the change in each of these inflammatory cytokines from before surgery to 24 h after surgery by CN-105 dose level. We will also examine CSF inflammatory cytokine levels 6 weeks +/- 3 weeks after surgery in all study patients to evaluate whether CN-105 treatment results in a long lasting reduction of CSF inflammatory cytokine levels.

Cognitive Data Analysis: We will compare continuous cognitive index change scores (Newman MF et al, 2001) between patients who receive CN-105 vs placebo using a 2-sided t-test. Based on the cognitive change variance seen in MADCO-PC patients (SD=0.21), this study (with ≥117 CN-105 treated and ≥ 39 placebo treated patients) will provide 80% power with 〈=0.05 to detect a half SD difference (i.e. 0.1 units) in CCI score between CN-105 vs placebo treated patients. Post-hoc ANOVA will determine if there are differences in cognitive index values by CN-105 dose level. We will also perform a chi-squared analysis (or Fisher's exact test if needed) to determine whether the rate of POCD as a dichotomous outcome (defined by a 1 SD drop in any of the 4 cognitive domains we have previously studied- 1- verbal memory, 2- visual memory, 3- attention/concentration, and 4- executive function) differs between CN-105 vs placebo treated patients.

Delirium analysis: Patients will be divided into those with delirium (i.e., 1 or more positive delirium

assessments) vs those without delirium (no positive delirium assessments). We will compare delirium rates between CN-105 treated and placebo treated patients using a two-sided chi-square test. With an expected delirium rate of 40% in placebo treated patients, this study (with >117 CN-105 treated patients, and >39 placebo treated patients) will provide 80% power to detect a 25% lower absolute rate of delirium in CN-105 vs placebo treated patients. Post-hoc ANOVA testing will evaluate for differences in delirium rates between CN-105 drug dose level groups, with post-hoc pairwise comparison tests as appropriate. Additional post-hoc analyses will evaluate delirium duration and severity between CN-105 vs placebo treated patients.

10.2 Sample Size

We planned to enroll up to 250 patients total in this phase 2 escalating dose study, to obtain three successive groups of 67 randomized patients each. Because enrollment happens at consent, and not all patients that consent will be randomized, we will keep enrolling patients until we reach 67 consented and randomized patients in each cohort. In each group of 67 patients, 50 will receive a given dose of CN-105 and 17 will receive placebo. In our recent MADCO-PC study, ~80% of patients returned for the 6 week assessment. Thus, enrolling 67 patients in each group ensures ≥52 patients (i.e. 39 CN-105-treated; 13 placebo-treated) will complete the 3 month postop assessment.Due to a clerical error by the Investigational Drug Services that prepares our drug/placebo doses, 69 patients were randomized to the first patient cohort rather than 67. . There will be no baseline preoperative cognitive status inclusion/exclusion criteria. Our prior work suggests ~15% of Duke orthopedic surgery patients over age 60 have MCI, ~5% have dementia, and ~80% are cognitively normal. All primary analyses here will be performed on patients grouped together (independent of baseline cognitive status); exploratory analyses will examine effects stratified by baseline cognitive strata, dose level, and by APOE4 carrier status.

Given the aforementioned expected 5% AE rates in placebo-treated patients, at completion we will have >80% power (with a=0.05) in a two-sample two-sided unpooled variance chi-square test to detect an absolute difference of 14.8% between grade II or higher AE rates seen in placebo vs CN-105 treated patients (pooling the three dose levels of CN-105 together). We will also track the rates of other grade II or higher AEs recorded by the patient's surgical team in the medical record; at study completion we will use 2-sided t tests to compare the rates of any other grade II or higher AEs between placebo vs CN-105 treated patients.

10.3 Measures to Minimize Bias

10.3.1 Enrollment/Randomization/Masking Procedures

Participants will be randomized to receive either CN-105 or placebo after they consent to participate in the trial, within 1 week prior to their index surgery. Participants will be enrolled in 1 of 3 dose cohorts (see Table 1) of 67 subjects each. An independent statistician will prepare dose stratified mixed block size randomization lists in a 3 to 1 ratio to achieve the desired distribution of drug and placebo. In each block of 67 trial participants, 50 will receive a given dose of CN-105 and 17 will receive placebo, in a double-blind fashion. CN-105 will be dissolved in normal saline, and either it or normal saline placebo will be administered as an IV drip over 15-30 minutes. Due to a clerical error by the Investigational Drug Services that prepares our drug/placebo doses, 69 patients were randomized to the first patient cohort rather than 67. Additionally, because enrollment happens at consent, and not all patients that consent will be randomized, we will keep enrolling patients until we reach 67 consented and randomized patients in each of the last two cohorts.

CN-105 and placebo will be prepared in identical bags labelled simply with the trial participant's number (1–203) to keep all trial staff and clinicians blinded to randomization. Only the

investigational pharmacist will have the randomization list that explains which drug (CN-105 or placebo) each participant is to receive and what dose of CN-105 or placebo will be administered every 6 hours (+/- 90 minutes) starting within 1 h before the scheduled or actual start of anesthesia/surgery and continuing until the end of postoperative day 3 (or when the participant is discharged home, whichever comes first), up to a maximum of 13 doses. Each case start time will be defined by the study team as either the start of the surgery/procedure or the start of anesthesia. In order to minimize bias, investigators conducting the laboratory assays on the CSF and blood samples will be blinded to trial group assignment during the course of the trial.

10.3.2 Procedures to Ensure the Success of Blinding

All personnel directly involved with study subjects (e.g., the PI, nurses, study coordinator, laboratory staff) will be blinded to treatment assignment (i.e. CN-105 vs placebo). All assays will be performed by laboratory staff blinded to treatment group/randomization.

The independent study statistician will prepare a randomization assignment log/schedule for the study, which only he and the investigational pharmacy staff will have access to, the PI, the primary study statistician, the co-investigator(s), nor any other members of the study team will have access to the randomization assignment log/schedule. The randomization assignment log/schedule will be saved in a password protected file, to which only the independent study statistician and the investigational pharmacy staff will have access. The randomization assignment log/schedule will not be pulled up on a computer screen anytime the PI, primary study statistician, co-investigator(s) or any other members of the study team are in the room. Pharmacy personnel who will prepare the study drug will be un-blinded (by necessity). Pharmacy personnel will prepare infusion bags of CN-105 and placebo and label each bag with the subject number for whom it was intended.

For DSMB reports, the primary study statistician will pull data in a blinded fashion and give it to the independent study statistician, who will prepare a report containing analysis of the unblinded data for the DSMB plus.

In the event of blinding failure, we will document the exact nature of the unblinding incident and take it into account during study analysis.

The independent statistician will share the randomization list with the primary study statistician only upon the completion of all 6 week postoperative follow-up visits, and only after all data has been double-entered into the study redcap database and checked for accuracy by both the primary study statistician and/or the PI (or another individual blinded to randomization and selected/designated to complete this task by the PI). The primary study statistician (Ms. Mary Cooter) will remain blinded through the duration of the study, and will be unblinded (and receive a copy of the randomization assignment log/schedule) only once all assays have been completed and all data have been double entered into the study database (with any disagreements resolved), i.e. once the final study data set is completed and locked. Only at this point will the primary study statistician be unblinded, in order to complete the final study data analysis.

In the event of a suspected safety issue regarding the study drug and to ensure patient safety, the DSMB-Plus has the authority to unblind the study PI and the project manager at any time during the study if necessary. If the study continues, the primary study statistician and the rest of the study team will remain blinded to help ensure the integrity of data collection and analysis.

11 SOURCE DOCUMENTS AND ACCESS TO DATA/DOCUMENTS

Source data are all information, original records of clinical findings, observations, or other activities in a clinical trial necessary for the reconstruction and evaluation of the trial. Examples of these original documents and data records include, but are not limited to, CRFs, hospital records, clinical and office charts, laboratory notes, memoranda, participant records, recorded audio tapes of counseling sessions, recorded data from automated instruments, copies or transcriptions certified after verification as being accurate and complete, microfiches, photographic negatives, microfilm or magnetic media, x-rays, and participant files and records kept at the pharmacy, laboratories, and medico-technical departments involved in the clinical trial. Access to source data will be limited to members of the research team who have undergone appropriate regulatory training and who are listed on the IRB protocol for this study.

To ensure that anyone who would access the patient medical record has adequate knowledge that the patient is participating in a clinical trial, the study coordinator will enter a note into each consented patient's Duke medical record indicating that the patient has been consented into this study.

12 QUALITY ASSURANCE AND QUALITY CONTROL

The principle investigator and study team will ensure that this clinical trial is conducted according to written SOPS and that data are generated, documented (recorded), and reported in compliance with the protocol, GCP, and the applicable regulatory requirements (e.g., Good Laboratory Practices, Good Manufacturing Practices).

The study team will provide direct access to all trial-related sites, source data/documents, and reports for the purpose of monitoring and auditing by local and regulatory authorities.

The clinical trial personnel will receive training in maintenance of all of the following:

1. Personnel signature log
2. Correspondence log (e-mail and phone)
3. Trial protocol and amendments signed by investigator

   Copy of IRB/EC-approval

   Copy of IRB/EC-approved ICF and amendments

   Copy of laboratory certification

   Copy of investigator's curriculum vita

   Copy of IRB/EC membership that approved the trial and name of the chair (if possible)

   Copy of training and audit reports
4. Updated subject enrollment/completed log
5. File with the identification numbers of case report forms (CRFs) corresponding to serum/CSF samples
6. File of completed CRFs and signed ICFs

Case report forms will be locked in the PI's office, the offices of Clinical Anesthesiology Research Endeavors staff, or stored on Duke University Health System (DUHS) or Department of Anesthesiology computer servers within the Duke firewall.

Quality control procedures will be implemented beginning with the data entry system, and data QC checks that will be run on the database will be generated. Any missing data or data anomalies will be communicated to the site for clarification/resolution.

The accuracy of data analysis will be verified by each investigator reviewing the primary data and its analysis. The accuracy and precision of laboratory assays will be assured with the measurement of appropriate controls for each assay. Investigators conducting the laboratory assays will be blinded as to a participant's (and sample's) trial arm assignment, thus eliminating the possibility of investigator bias while conducting these assays.

13 ETHICS/PROTECTION OF HUMAN SUBJECTS

13.1 Ethical Standard

The investigator will ensure that this trial is conducted in full conformity with regulations for the protection of human subjects of research codified in 45 Code of Federal Regulations [CFR] Part 46, 21 CFR Part 50, 21 CFR Part 56, and/or the International Council on Harmonisation E6.

13.2 Institutional Review Board

The protocol, ICF, recruitment materials, and all participant materials will be submitted to the IRB for review and approval. Approval of both the protocol and the consent form must be obtained before any participant is enrolled. Any amendment to the protocol will require review and approval by the IRB before the changes are implemented to the trial. All changes to the consent form will be IRB approved; a determination will be made regarding whether previously consented participants need to be reconsented.

13.3 Informed Consent Process

13.3.1 Consent and Other Informational Documents Provided to Participants

Consent forms describing in detail the study agent, trial procedures, and risks are given to the participant, and written documentation of informed consent is required before administering study product.

13.3.2 Consent Procedures and Documentation

Patients will be consented by one of the trial investigators or coordinators, all of whom are trained in participant enrollment. If the patient consents to participate in the trial, the trial coordinator will then set up an appointment for the patient to have preoperative or baseline cognitive testing. Furthermore, given concerns over COVID-19 transmission and safety for both research participants and study staff, when possible we use electronic consent (eConsent) for patients using the REDCap (Research Electronic Data Capture), which is a secure, web-based application designed to support data capture. Participants are sent the currently approved version of the consent via email, at which point the study staff can go over this with the patient (either in person or over a virtual platform like Zoom). If the consent is done using the electronic consent, the patient provides a signature at the end (using their finger or a mouse/cursor), and once they accept, the ICF is saved in the REDCap database and a copy is emailed to the patient. Duke implemented eConsent through REDCap last year, and the files live behind Duke's firewall on Duke servers.

Informed consent will be obtained from each participant himself or herself whenever possible. In those situations in which a participant is not competent to give informed consent, then the LAR will provide the consent on behalf of the participant. We will initially use consent of the LAR for participants deemed not to have medical decision-making capacity; however, should these participants regain medical decision-making capacity after enrollment, they will be re-consented before continuation of participation in trial. The participant's capacity to consent will be assessed at each research visit by a qualified licensed person per DUHS policy.

If a participant is able to give informed consent to participate in the trial but physically cannot sign the consent form (due to blindness, hand injury, or other physical impairment), the participant's LAR may sign the consent form on behalf of and at the direction of the participant. In such cases, the participant must be able to understand the potential risks, benefits, and alternatives to trial participation, and all questions and concerns of the participant will be addressed. Participants or their LARs will sign all consent paperwork before taking part in any trial procedures or cognitive testing.

13.4 Participant and Data Confidentiality

Participant confidentiality is strictly held in trust by the participating investigators, their staff, and the sponsor and their agents. This confidentiality is extended to cover testing of biological samples in addition to the clinical information relating to participants. Therefore, the trial protocol, documentation, data, and all other information generated will be held in strict confidence. No information concerning the trial or the data will be released to any unauthorized third party without previous written approval of the sponsor.

Representatives of the IRB or pharmaceutical company supplying study product may inspect all documents and records required to be maintained by the investigator, including but not limited to, medical records (office, clinic, or hospital) and pharmacy records for the participants in this trial. The clinical trial site will permit access to such records.

The trial participant's contact information will be securely stored for internal use during the trial. At the end of the trial, all records will continue to be kept in a secure location for as long a period as dictated by the Duke IRB and institutional regulations.

Trial participant research data, which is for purposes of statistical analysis and scientific reporting, will be kept in the Duke Anesthesiology department. This will not include the participant's contact or identifying information. Rather, individual participants and their research data will be identified by a unique trial identification number. The trial data entry and trial management systems used by clinical sites and by research staff will be secured and password protected. At the end of the trial, all trial databases will be de-identified and archived in the Duke Anesthesiology Department.

13.5 Research Use of Stored Human Samples, Specimens, or Data

Data collected for this trial will be analyzed and stored at the Duke Anesthesiology Clinical Anesthesia Research Endeavors offices or other Duke Anesthesiology Department offices, as appropriate. After the trial is completed, the de-identified, archived data will be stored for use by other researchers, including those outside of the trial.
Permission to transmit data to this data repository will be included in the informed consent.

With the participant's approval and as approved by local IRBs, de-identified biological samples will be stored at Duke with the same goal as the sharing of data with collaborating investigators. These samples could be used for research into the causes of POCD and delirium, as well as AD, their complications and other conditions for which older surgical patients are at increased risk, and to improve treatment. Our repository will also be provided with a code-link that will allow linking the biological specimens with the phenotypic data from each participant, maintaining the masking of the identity of the participant.

During the conduct of the trial, an individual participant can choose to withdraw consent to have biological specimens stored for future research. However, withdrawal of consent with regard to biosample storage will not be possible after the trial is completed.

When the trial is completed, access to trial data and/or samples will be controlled by the Duke Neurologic Outcomes Research Group.

14 DATA HANDLING AND RECORDKEEPING

14.1 Data Collection and Management Responsibilities

Forms describing the collection of CSF and plasma aliquots will be completed at every sample draw. Case report forms will be completed at the time of enrollment and during the operative procedure. Data collected will incorporate variables reported in the subject's medical record and anesthetic record as dictated by standard of care practices and will include, but not be limited to, imaging studies, neurological exam, records of any postoperative infections or antibiotic usage, microbiological culture data, records of any delayed wound healing or other wound healing issues, medications, presence of mechanical ventilation and/or tracheostomy, laboratory profiles, ECGs, and type of diet.

14.2 Trial Records Retention

Trial documents will be retained for a minimum of 2 years after a marketing application is approved for the drug, or, if an application is not approved for the drug, until 2 years after shipment and delivery of the drug for investigational use is discontinued and FDA has been notified. Per Duke IRB regulations, all trial documents will be retained for 6 years after the completion of the study.

14.3 Protocol Deviations

A protocol deviation is any noncompliance with the clinical trial protocol, GCP, or manual of procedures requirements. The noncompliance may be on the part of either the participant, the investigator, or the trial site staff. As a result of deviations, corrective actions are to be developed by the site and implemented promptly.

These practices are consistent with ICH E6:

4.5 Compliance with Protocol, sections 4.5.1, 4.5.2, and 4.5.3

5.1 Quality Assurance and Quality Control, section 5.1.1

5.20 Noncompliance, sections 5.20.1, and 5.20.2.

It is the responsibility of the study team to use continuous vigilance to identify and promptly report

deviations to the IRB. All deviations must be addressed in trial source documents. The site PI/trial staff is responsible for knowing and adhering to their IRB requirements.

14.4 Publication and Data Sharing Policy

This trial will comply with the NIH public access policy, which ensures that the public has access to the published results of NIH-funded research. It requires scientists to submit final peer- reviewed journal manuscripts that arise from NIH funds to the digital archive PubMed Central upon acceptance for publication.

FDAAA mandates that a "responsible party" (i.e., the sponsor or designated PI) register and report results of certain "applicable clinical trials". Since this is an applicable clinical trial, the study team will register the trial with clinicaltrials.gov and will report all results as required.

15 CONFLICT OF INTEREST POLICY

The independence of this trial from any actual or perceived influence, such as by the pharmaceutical industry, is critical. Therefore, any actual conflict of interest of persons who have a role in the design, conduct, analysis, publication, or any aspect of this trial will be disclosed and managed. Furthermore, persons who have a perceived conflict of interest will be required to have such conflicts managed in a way that is appropriate to their participation in the trial. The trial leadership has established policies and procedures for all trial group members to disclose all conflicts of interest and will establish a mechanism for the management of all reported dualities of interest.

This is a blinded study (which should reduce bias), and the study PI (Dr. Berger) himself has no COI with respect to this study, CN-105 itself, or AEGIS CN. Nonetheless since Duke University itself owns the IP on CN-105 (which was discovered and developed at Duke), Duke itself has a conflict of interest with respect to this CN-105 trial. Because of Duke's conflict of interest, the study PI (Dr. Berger) has met with the Duke University Research Integrity Office and the Conflict of Interest Committee to discuss this. The Duke Research Integrity Office and this Committee have agreed that this study can proceed at Duke under the approval of an External Institutional Review Board and the monitoring of an external DSMB plus, provided that the DSMB plus is comprised of qualified individuals from outside Duke who themselves have no other conflicts of interest, and that the study is reviewed and approved by an external IRB.

16 LITERATURE REFERENCES

1. Yancey AK, Ortega AN, Kumanyika SK. Effective recruitment and retention of minority research participants. *Annu Rev Public Health*. 2006;27:1-28. doi:10.1146/annurev.publhealth.27.021405.102113.

2. Monk TG, Weldon BC, Garvan CW, et al. Predictors of cognitive dysfunction after major noncardiac surgery. *Anesthesiology*. 2008;108(1):18-30. doi:10.1097/01.anes.0000296071.19434.1e.

3. Leung JM, Sands LP, Wang Y, et al. Apolipoprotein E e4 allele increases the risk of early postoperative delirium in older patients undergoing noncardiac surgery. *Anesthesiology*. 2007;107(3):406-411. doi:10.1097/01.anes.0000278905.07899.df.

4. James ML, Sullivan PM, Lascola CD, Vitek MP, Laskowitz DT. Pharmacogenomic effects of apolipoprotein e on intracerebral hemorrhage. *Stroke J Cereb Circ*. 2009;40(2):632-639. doi:10.1161/STROKEAHA.108.530402.

5. Wang H, Christensen DJ, Vitek MP, Sullivan PM, Laskowitz DT. APOE genotype affects outcome in a murine model of sepsis: implications for a new treatment strategy. *Anaesth Intensive Care*. 2009;37(1):38-45.

6. Wilcock DM, Gharkholonarehe N, Van Nostrand WE, Davis J, Vitek MP, Colton CA. Amyloid reduction by amyloid-beta vaccination also reduces mouse tau pathology and protects from neuron loss in two mouse models of Alzheimer's disease. *J Neurosci* 2009;29(25):7957-7965. doi:10.1523/JNEUROSCI.1339-09.2009.

7. Lo RY, Hubbard AE, Shaw LM, et al. Longitudinal change of biomarkers in cognitivedecline. *Arch Neurol*. 2011;68(10):1257-1266. doi:10.1001/archneurol.2011.123.

8. Berger M, Nadler JW, Friedman A, et al. The Effect of Propofol Versus Isoflurane Anesthesia on Human Cerebrospinal Fluid Markers of Alzheimer's Disease: Results ofa Randomized Trial. *J Alzheimers Dis JAD*. 2016;52(4):1299-1310. doi:10.3233/JAD-151190.

9. Cwikel JG, Fried AV, Biderman A, Galinsky D. Validation of a fall-risk screening test, the Elderly Fall Screening Test (EFST), for community-dwelling elderly. *Disabil Rehabil.* 1998 May;20(5):161-7.

10. Tromp AM, Pluijm SM, Smit JH, Deeg DJ, Bouter LM, Lips P. Fall-risk screening test: a prospective study on predictors for falls in community-dwelling elderly. *J Clin Epidemiol.* 2001 Aug;54(8):837-44.

11. Guptill JT, Raja SM, Boakye-Agyeman F, Noveck R, Ramey S, Tu TM, Laskowitz DT.Phase 1 Randomized, Double-Blind, Placebo-Controlled Study to Determine the Safety, Tolerability, and Pharmacokinetics of a Single Escalating Dose and Repeated Doses of CN-105 in Healthy Adult Subjects. *J Clin Pharmacol*. 2017 Jun;57(6):770-776. doi: 10.1002/jcph.853.

12. Giattino CM, Gardner JE, Sbahi FM, Roberts KC, Cooter M, Moretti E, Browndyke JN, Mathew JP, Woldorff MG, Berger M; MADCO-PC Investigators. Intraoperative Frontal Alpha-Band Power Correlates with Preoperative Neurocognitive Function in Older Adults. *Front Syst Neurosci.* 2017 May 8;11:24. doi: 10.3389/fnsys.2017.00024

13. Corriveau Ra, Koroshetz Wj, Gladman Jt, Jeon S, Babcock D Et Al. Alzheimer's Disease-Related Dementias Summit 2016: National Research Priorities. *Neurology*. 2017 Dec 5;89(23):2381-2391. Doi: 10.1212/Wnl.0000000000004717.

14. Berger M, Burke J, Eckenhoff R, Mathew J. Alzheimer's disease, anesthesia, and surgery: a clinically focused review. *J Cardiothorac Vasc Anesth.* 2014 Dec;28(6):1609-23. doi: 10.1053/j.jvca.2014.04.014

15. Berger M, Nadler JW, Browndyke J, Terrando N, Ponnusamy V, Cohen HJ, Whitson HE, Mathew JP. Postoperative Cognitive Dysfunction: Minding the Gaps in Our Knowledge of a

Common Postoperative Complication in the Elderly. *Anesthesiol Clin.* 2015 Sep;33(3):517-50. doi: 10.1016/j.anclin.2015.05.008.

16. Almeida C, Choy EH, Hewlett S, et al. Biologic interventions for fatigue in rheumatoid arthritis. *Cochrane Database Syst Rev*. 2016(6): CD008334. 69.

17. Bae SC, Gun SC, Mok CC, et al. Improved health outcomes with etanercept versus usual DMARD therapy in an Asian population with established rheumatoid arthritis. BMC *Musculoskelet Disord.* 2013; 14:13.

18. Newman MF, Kirchner JL, Phillips-Bute B, Gaver V, Grocott H, Jones RH, Mark DB, Reves JG, Blumenthal JA; Neurological Outcome Research Group and the Cardiothoracic Anesthesiology Research Endeavors Investigators. Longitudinal assessment of neurocognitive function after coronary-artery bypass surgery. *N Engl J Med*. 2001 Feb 8;344(6):395-402.

19. Marcantonio ER, Ngo LH, O'Connor M, Jones RN, Crane PK, Metzger ED, Inouye SK. 3D-CAM: derivation and validation of a 3-minute diagnostic interview for CAM-defined delirium: a cross-sectional diagnostic test study. *Ann Intern Med.* 2014 Oct 21;161(8):554-61. doi: 10.7326/M14-0865.

20. Ely EW, Margolin R, Francis J, May L, Truman B, Dittus R, Speroff T, Gautam S, Bernard GR, Inouye SK. Evaluation of delirium in critically ill patients: validation of the Confusion Assessment Method for the Intensive Care Unit (CAM-ICU). *Crit Care Med.* 2001 Jul;29(7):1370-9.

21. Kuczmarska A, Ngo LH, Guess J, O'Connor MA, Branford-White L, Palihnich K, Gallagher J, Marcantonio ER: Detection of Delirium in Hospitalized Older General Medicine Patients: A Comparison of the 3D-CAM and CAM-ICU. *J Gen Intern Med* 2016; 31: 297-303

22. *Medical Research Council Institute of Hearing Research. Safety Policy Document*. Nottingham: MRC Institute of Hearing Research; 1996.

23. Speer ME, Soldan A. Cognitive reserve modulates ERPs associated with verbal working memory in healthy younger and older adults. Neurobiol Aging. 2015 Mar;36(3):1424-34. doi: 10.1016/j.neurobiolaging.2014.12.025. Epub 2014 Dec 29. PMID: 25619663; PMCID: PMC4346428.

Statistical Analysis Plan

Modulating **A**poE signalling to **R**educe **B**rain inflammation, de**L**irium and postop**E**rative cognitive dysfunction (**MARBLE**):

A Phase 2 Trial to Evaluate the Efficacy and Feasibility of the Apolipoprotein E Mimetic Peptide, CN-105, in Preventing Postoperative Cognitive Dysfunction and Delirium and Blocking Their Underlying Neural Mechanisms

SAP Version: 3.0

SAP Date: 1/19/2024

Statistician: Mary Cooter Wright

Primary Investigator: Miles Berger

Duke Health Department of Anesthesiology

IRB: Pro00088855

FDA IND#: 142091

ClinicalTrials.gov Identifier: NCT03802396

Revisions/Notes:

Study protocol and methods originally described in publication: VanDusen et. al. J Alzheimers Dis. 2020;75(4):1319-1328. doi: 10.3233/JAD-191185 and formally summarized herein

V2.0 4/25/2023 Revised methods for AE analysis and FWER control

V3.0 1/19/2024 Finalized definition of per-protocol cohort. Finalized adjustment covariates in multivariable models

Introduction: Postoperative cognitive dysfunction (POCD; also known as Neurocognitive Disorder-Postoperative, or NCD) and delirium each occur in up to 40% of the ≥16 million adults age ≥60 who undergo surgery each year, and both are associated with decreased quality of life, increased one-year mortality, and a possible increased dementia risk. While there are several behavioral interventions such as the Hospital Elder Life Program (HELP) and the ABCDEF bundle that have demonstrated efficacy for reducing delirium incidence, there are no currently FDA-approved drugs for preventing delirium or other types of PND, likely due to our poor understanding of their pathogenesis.

One potential target implicated in both neuro-inflammation and AD pathology is the late-onset AD genetic risk factor APOE4, whose protein product apoE4 increases glial activation and pro-inflammatory cytokine levels in animal models. In murine models, blocking apoE4 with the apoE mimetic peptide drug CN-105 reduced neuro-inflammation and improved cognitive, neurobehavioral, and motor outcomes in traumatic brain injury, ischemic stroke, and cerebral hemorrhage models. Additionally, a phase I trial found that CN-105 was safe at doses up to 20 times greater than those given in preclinical studies, with no serious adverse events (SAEs).

Based on these data, we hypothesize that CN-105 will be safe and effective for preventing PND in older adults. Thus, we have initiated MARBLE, a phase II randomized controlled trial (RCT) to determine the safety, feasibility and efficacy of perioperative CN-105 treatment in older surgical patients at risk for PND.

1. **Study Objective:** MARBLE is a phase II clinical trial designed to evaluate the maximum safe dose of perioperative CN-105 administration in older non-cardiac, non-neurological surgery patients. It is secondarily intended to evaluate the feasibility and potential of CN-105 for preventing PND and reducing postoperative neuroinflammation.

2. **Hypotheses:**
   a. Primary: CN-105 will be safe resulting in similar rates of adverse events as the placebo patients
   b. Secondary
      i. CN-105 administration will be feasible with 90% or more of study doses given within the protocol specified time window
      ii. CN-105 will decrease neuroinflammation (as measured by CSF cytokine levels) compared to placebo treated patients
      iii. CN-105 will improve CCI change from baseline to 6 weeks compared to placebo treated patients
      iv. CN-105 will decrease the incidence and severity of postoperative delirium compared to placebo treated patients

3. **Study population or dataset:**

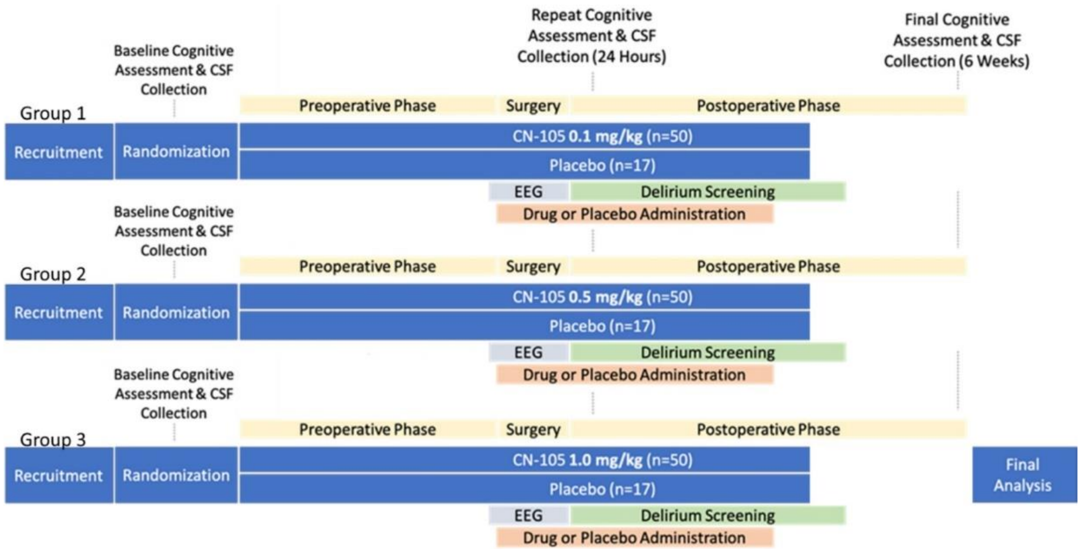

Sample Size Calculations: Based on prior studies, we expect ≥80% of patients to complete all aspects of the study (drug administration, blood and CSF sampling, neurocognitive testing, EEG recording, quality of life assessments, and delirium screening), yielding ≥117 CN-105-treated patients across the three dose groups and ≥39 placebo-treated patients. We anticipate an AE incidence of 5% among placebo-treated patients, based on prior AE rates in older Duke non-cardiac surgical patients. Based on these parameters, this sample size provides >80% power (with α=0.05) in a two- sample two-sided un-pooled variance chi-square test to detect an absolute difference of 14.8% between AE rates in placebo versus CN-105 treated patients.

Randomization: After enrollment, participants are randomized to receive CN-105 or placebo at a 3:1 ratio. There are three successive escalating dose groups of 67 patients; in each group, 50 patients receive CN-105 and 17 receive placebo. CN-105 dosage is 0.1 mg/kg, 0.5 mg/kg and 1 mg/kg in the three groups, respectively. An independent statistician will prepare dose stratified mixed block size randomization lists in a 3 to 1 ratio to achieve the desired distribution of drug and placebo.

Blinding: In each block of 67 trial participants, 50 will receive a given dose of CN-105 and 17 will receive placebo, in a double-blind fashion. CN-105 will be dissolved in normal saline, and either it or normal saline placebo will be administered as an IV drip over 15-30 minutes. CN-105 and placebo will be prepared in identical bags labelled simply with the trial participant's number (1–201), to keep all trial staff and clinicians blinded to randomization. Only the investigational pharmacist will have the randomization list that explains which drug (CN-105 or placebo) each participant is to receive and what dose of CN-105 or placebo will be administered every 6 hours (+/- 90 minutes) starting within 1 h before the start of anesthesia and continuing until the end of postoperative day 3 (or when the participant is discharged home, whichever comes first), up to a maximum of 13 doses. In order to minimize bias, investigators conducting the laboratory assays on the CSF and blood samples will be blinded to trial group assignment during the course of the trial. All personnel directly involved with study subjects (e.g., the PI, nurses, study coordinator, laboratory staff) will be blinded to treatment assignment (i.e. CN-105 vs placebo). Pharmacy personnel who will prepare the study drug will be unblinded (by necessity). Pharmacy personnel will prepare infusion bags of CN-105 and placebo and label each bag with the subject number for whom it was intended. Study statistician (MCW) will remained blinded to group until analysis datasets are locked and analysis for primary and secondary analyses are completed.

Inclusion/Exclusion

In order to be eligible to participate in this trial, an individual must meet all of the following criteria:

1. Age ≥ 60
2. Ability to speak English
3. Undergoing non-cardiac, non-neurologic surgical procedures; surgery scheduled to last > 2 hours; due to be admitted to the hospital following surgery

An individual who meets any of the following criteria will be excluded from participation in this trial:

1. Inmate of a correctional facility
2. Scheduled to receive systemic chemotherapy between the time of the two cognitive testing sessions
3. Known inability to undergo LPs, due to anticoagulant use, severe anxiety, or other clinical contraindication known ahead of time.
4. Inappropriate for study inclusion based on the judgement of the principal investigator.
5. If a patient undergoes major head trauma that occurs between the time of the two cognitive testing sessions, then they will be withdrawn from the study.

Stopping Rule: MARBLE may be stopped if after any group of 67 patients the rate of grade III or higher SAEs (per 2018 CTCAE guidelines [42]) in drug-treated patients is >10% and more than three times the rate of such events in the placebo-treated group. Considering the wide range of surgical procedures and patient comorbidities that could contribute to AEs in this study, the DSMB+ has been advised to use both this quantitative cutoff and its clinical judgement in considering whether to recommend that the study be stopped or not.

4. Outcomes:

Primary Outcome Measure: Rate of adverse events (AEs) of grade II or higher, per CTCAE criteria through 6-week study follow-up period

Secondary Outcome Measures:

1. The feasibility of perioperative CN-105 administration is assessed by tracking the percentage of doses given within the correct time window based on study team prescribed dosing schedule and medical administration record (MAR) (i.e. within 1 hour prior to the scheduled or actual start time of the surgery, and within a +/- 90 minute time window for subsequent doses, which are administered every 6 hours after the start of surgery).
2. Change in cerebrospinal fluid (CSF) IL-6, Il-8, MCP-1, G-CSF cytokine levels between drug vs placebo treated patients. Participants undergo baseline CSF and blood sampling within two months prior to surgery; repeat CSF and blood samples are obtained 24 ± two hours after the start of surgery, and six ± three weeks after surgery. CSF specimens are used to determine the effects of CN-105 treatments on levels of the cytokines IL-6, IL-8, G-CSF, and MCP-1 using Meso Scale Discovery multiplex assays
3. Baseline to 6-week Change in cognitive change index (CCI) and cognitive domains between drug vs placebo treated patients as well as incidence of NCD.
   Cognition is assessed with a standard test battery (Table below), by staff trained by a board-certified neuropsychologist within two months before and again six weeks after surgery. Individual test scores will be combined by factor analysis into cognitive domain factors. The mean of these cognitive domain factor scores yields the Continuous Cognitive Index (CCI), and CCI change from before to after surgery thus quantifies the degree of learning/cognitive improvement or cognitive decline. Since changes in specific cognitive domains have been observed from before to after surgery, we will also examine the change in each the individual cognitive domain factor scores from before to six weeks after surgery between drug vs placebo treated patients.

   The incidence of NCD-postoperative, mild and/or major, between drug- vs placebo- treated patients will also be examined as an exploratory outcome. NCD postoperative (mild) will be defined as a 1–2 standard deviation (SD) decrease in score on any one of the four cognitive domain factors (which are used in calculating the Continuous Cognitive Index as described above) combined with a subjective cognitive complaint. Major NCD, postoperative will be defined as a ≥2 SD drop in any of these four cognitive domain factors. Here, a 1 or 2 standard deviation in any one of the four cognitive domain factors refers to the standard deviation in each of these factors in the entire population under study here at the baseline/preoperative timepoint. Subjective cognitive complaints will be assessed using the Cognitive Difficulties Scale, which our group has previously used to examine the association between subjective and objective cognitive deficits after surgery. The Cognitive Difficulties Scale is administered to MARBLE study patients both before and six weeks after surgery. NCD postoperative (major) will be defined as a 2 SD decrease in any one of the four cognitive domain factors, combined with a postoperative deficit in ability to perform one or more activities of daily living (ADLs). Patients’ ability to perform ADL’s will be assessed using the Duke Activity Status Index, which is administered to MARBLE study patients both before and six weeks after surgery.

The MARBLE Cognitive Testing Battery

Test	Cognitive Function Assessed
Brief Visuospatial Memory Test, Revised	Visuospatial learning and recall
Controlled Oral Word Association Test	Verbal Fluency and Information Retrieval
Hopkins Verbal Learning Test, Revised	Auditory learning and verbal recall
Lafayette Grooved Pegboard Test	Manual dexterity and motor speed
Montreal Cognitive Assessment	Mild cognitive impairment screening
Trail Making Test, Parts A & B	Complex executive functioning skills (eg, logical task switching)
Wechsler Adult Intelligence Scale, 3rd Revision Digit Span Subtest	Immediate auditory-verbal recall and complex attention
Wechsler Adult Intelligence Scale, 3rd Revision Digit Symbol Coding Subtest	Visual scanning and visuomotor production
Wechsler Test of Adult Reading	Premorbid intellectual function

4. Incidence and severity of delirium between drug vs. placebo treated patients
   Delirium is assessed at the initial baseline study visit and twice daily during postoperative days one through day five using the 3D-CAM in non-intubated patients and the CAM-ICU in intubated patients. Delirium assessors are trained with materials from the Hebrew SeniorLife Program/Harvard Medical School SAGES study group, and begin conducting delirium assessments on study patients only once they demonstrate ≥90% agreement with standardized training assessments. To date, the accuracy of our delirium assessors measured using these standardized video-taped assessments is 93%, and all assessors receive feedback to further improve their accuracy after this process. Delirium severity will be assessed by the 3D-CAM as well. Scores on the 3D CAM (in non-intubated patients) are used to determine delirium symptom severity based on a 0 - 20 point scale of the test.

5. Variables of Interest

Variable	Description
Randomization Assignment	Primary analysis will be 2 level drug groups (placebo vs. CN-105), and secondary analysis will incorporate dose level (1 v 2 v 3 vs placebo)
Age*	numeric
Sex	Male v Female
Race/Ethnicity*	As in NIH notice White, Black, Asian, Hispanic, Other (includes American Indian or Alaska Native, Native Hawaiian or other pacific Islander) Not reported/Refused
APOE Genotype*	APOE4 carrier v non-carrier
ASA PS	Anesthesiology rate case severity
Van Walraven Elixhauser Comorbidity index*	ICD based mortality risk index
Surgical Service*	Thoracic, Urology, Gynecology, General, Orthopedic,
surgery duration*	Minutes from incision to procedure end
Estimated blood loss*	volume
Baseline CCI	
Years of Education	

6. Statistical Methods and results

6.1. Data management:

Adherence and protocol deviations: Study drug administration will be cross-referenced between study team defined dosing schedule and the medication administration record (MAR). Additionally, 24h CSF samples will be analyzed for levels of CN-105 to determine if study drug reached measurable levels in the CSF.

Description of Analysis population: The ITT population will be defined by randomization assignment among those who underwent surgery on protocol, this will be a modified ITT as patients who were randomized but withdrawn prior to surgery will be excluded.

The per-protocol population will be defined as those who received ≥80% of study doses per schedule up to the time of outcome evaluation (eg. for 24h outcomes doses within 24h of first dose will be considered, whereas for the safety outcome all doses during hospitalization will be evaluated).

Statistical Software: Statistical analysis will be performed in either SAS v9.4 (SAS INC Cary, NC) or R v 4.2 or higher (R Foundation for statistical computing, Vienna, Austria).

6.2. Statistical Considerations:

Level of statistical significance: All hypothesis testing will be two-sided and statistical significance will be assessed at the alpha = 0.05 level

Multiplicity considerations: We will pursue multiple comparison correction (via Holm a step-down Bonferroni which is a FWER method to control error rate at the 0.05 level) for secondary and exploratory outcomes defined by multiple measures but not across aims.

For AEs by body system:

For inflammatory cytokines: we have prespecified

For cognitive domains:

Reporting metrics: We will explore numeric variable data distributions via visualizations (histograms, QQ plots) and assess normality via Shapiro-Wilks test. For numeric variables found to be normally distributed we will report mean (SD) and if non-normally distributed median [Q1, Q3]. Univariable analysis of numeric variables by treatment group will be either parametric or non-parametric based on data distributions. Categorical variables will be reported as count (%) and if data is sparse non-parametric methods will be used for hypothesis testing. All effect estimates will be provided with point estimates (mean difference, mean ratio, odds ratio, etc.) and variability (95% confidence intervals, SD, Quartiles, etc.).

Approach to missing data: Missing data will be categorized by cause, and baseline characteristics of patients who do not complete the study will be compared to those who do, to evaluate for response bias. Multiple imputation will be pursued if found necessary for the analysis of efficacy endpoints. In the case that imputation is used, sensitivity analyses will also be performed using only actual data to ensure that the results are not biased by the imputation strategy.

6.3. Analysis Plan Primary Outcome:

To study the primary safety outcome (composite rate of any Grade II or higher AE) we will first tabulate the incidence of events by event name by 2-level treatment and control groups, then calculate relative risks (95% CIs) and produce a rainfall plot visualization for the top 20 as below:

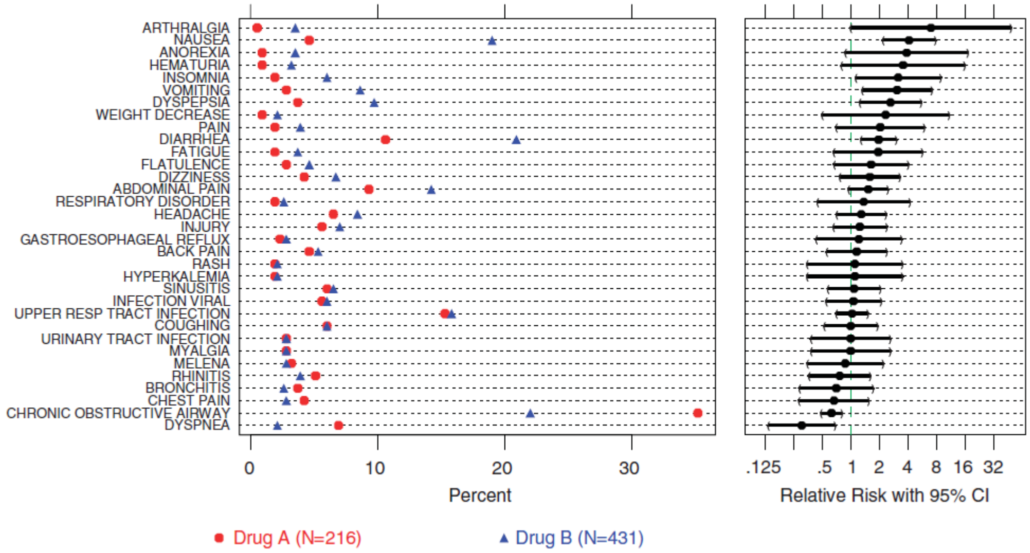

Figure 9. Most frequent on-therapy adverse events sorted by relative risk.

Next we will tabulate the incidence of events by system name, calculate relative risk (95% CI) and produce a rainfall plot visualization as above. Inference as to significance of a difference in safety events will be conducted via the Crowe method for multi-stage multiplicity correction. Which proceeds as follows:

Suppose there are s classes of events with k_i events in each class, $1 \leq i \leq s$. To apply DFDR using the updated methodology, apply the following steps:

(1) Calculate p^*_{ij} as the FDR-adjusted p-values within each of s event groups;
(2) Define $p^*_i = \min(p^*_{ij}, \quad 1 \leq j \leq k_i)$;
(3) Calculate p^{**}_i as the FDR-adjusted p-values of p^*_i;
(4) Define $F = \{p_{ij} | p^{**}_i \leq \alpha\}$. Calculate FDR-adjusted p-values $p^{(F)}_{ij}$ for all p_{ij} in F;
(5) Events are significant if $p^{**}_i \leq \alpha$ and $p^{(F)}_{ij} \leq \alpha$.

1) Analyze within each system, eg within Cardiac then within vascular, and do FDR correction

2) Take the smallest corrected p-value from each system

3) Perform FDR correction at the system level based on the minimum p from each system

4) For all systems with system corrected p-values below alpha, calculate event level FDR corrected p-values

5) If corrected p-value for events considered in part 4 are below alpha then the event has a significant difference

Citations:

Crowe BJ, Xia HA, Berlin JA, et al. Recommendations for safety planning, data collection, evaluation and reporting during drug, biologic and vaccine development: A report of the safety planning, evaluation, and reporting team. Clin Trials 2009; 6: 430–40.

Mehrotra DV, Adewale AJ. Flagging clinical adverse experiences: Reducing false discoveries without materially compromising power for detecting true signals. Stat Med 2012; 31: 1918–30.

For any events identified as significantly different between the treatment and control group we will conduct exploratory multivariable logistic regression analysis. Multivariable logistic regression analysis will include a term for treatment group (placebo v control) and adjustment terms for age, sex, race, APOE4 (carrier v non-carrier) Van Walraven Elixhauser comorbidity index, surgery duration, and EBL.

$$Binary\ Indicator\ of\ Event\ Incidence = \beta_0 + \beta_1 * treatment + \beta_L * L$$

Table X Logistic Regression OR Estimates for Adjusted Models of Binary Incidence of Grade II or higher AEs

Covariate	OR	95% C.I.	P-Value
Treatment Group (CN-105 v Placebo)	XXX	(XXX , XXX)	XXX
Sex (Female v Male)	XXX	(XXX, XXX)	XXX
Age	XXX	(XXX, XXX)	XXX
Race (Reference: White/Caucasian)			XXX
African American/Black	XXX	(XXX, XXX)	XXX
Asian	XXX	(XXX, XXX)	XXX
Other	XXX	(XXX, XXX)	XXX
APOE4 (Carrier v Non-Carrier)	XXX	(XXX, XXX)	XXX
Van Walraven Elixhauser Comorbidity Index	XXX	(XXX, XXX)	XXX
Surgery Duration (per hour)	XXX	(XXX, XXX)	XXX
Estimated Blood Loss (per 100 ml)	XXX	(XXX, XXX)	XXX

After completing primary analysis comparing the treatment to control groups we will pursue exploratory analysis comparing events between the 3 dose levels to controls. Should any event be indicated as having a significant dose specific difference we will pursue additional multivariable analysis as below:

$$Binary\ Incidence\ of\ Event\ Incidence = \beta_0 + \beta_1 * Dose1 + \beta_2 * Dose2 + \beta_3 * Dose3 + \beta_L * L$$

Table X Logistic Regression OR Estimates for Adjusted Models of Binary Incidence of Grade II or higher AEs

Covariate	OR	95% C.I.	P-Value
Dose Group (Reference Placebo)			XXX
CN-105 Dose 1	XXX	(XXX , XXX)	XXX
CN-105 Dose 2	XXX	(XXX , XXX)	XXX
CN-105 Dose 3	XXX	(XXX , XXX)	XXX
Sex (Female v Male)	XXX	(XXX, XXX)	XXX
Age	XXX	(XXX, XXX)	XXX
Race (Reference: White/Caucasian)			XXX
African American/Black	XXX	(XXX, XXX)	XXX
Asian	XXX	(XXX, XXX)	XXX
Other	XXX	(XXX, XXX)	XXX
APOE4 (Carrier v Non-Carrier)	XXX	(XXX, XXX)	XXX
Van Walraven Elixhauser Comorbidity Index	XXX	(XXX, XXX)	XXX
Surgery Duration (per hour)	XXX	(XXX, XXX)	XXX
Estimated Blood Loss (per 100 ml)	XXX	(XXX, XXX)	XXX

6.4. Analysis Plan Secondary Outcome 1:

The feasibility analysis will be performed via construction of confidence intervals for the rate of dose administration per protocol. Post-hoc analysis will be performed among drug treated patients and by dose level to determine if the drug itself or drug dose level affects feasibility.

<u>6.4. Analysis Plan Secondary Outcome 2:</u>
For our secondary efficacy endpoints, we will perform t-tests, Wilcoxon rank sum tests, or chi-square tests as appropriate to compare outcomes for drug and placebo treated patients. Subsequently we will use linear or logistic regression models to investigate potential dose-response and subgroup effects for each endpoint.

Model definition for binary outcomes:

$$Binary\ Indicator\ of\ Event\ Incidence = \beta_0 + \beta_1 * treatment + \beta_L * L$$

Table X Logistic Regression OR Estimates for Adjusted Models

Covariate	OR	95% C.I.	P-Value
Treatment Group (CN-105 v Placebo)	XXX	(XXX , XXX)	XXX
Sex (Female v Male)	XXX	(XXX, XXX)	XXX
Age	XXX	(XXX, XXX)	XXX
Race (Reference: White/Caucasian)			XXX
African American/Black	XXX	(XXX, XXX)	XXX
Asian	XXX	(XXX, XXX)	XXX
Other	XXX	(XXX, XXX)	XXX
APOE4 (Carrier v Non-Carrier)	XXX	(XXX, XXX)	XXX
Van Walraven Elixhauser Comorbidity Index	XXX	(XXX, XXX)	XXX
Surgery Duration (per hour)	XXX	(XXX, XXX)	XXX
Estimated Blood Loss (per 100 ml)	XXX	(XXX, XXX)	XXX

Model definition for numeric outcomes:

$$Outcome\ Value = \beta_0 + \beta_1 * treatment + \beta_L * L$$

Table X Linear Regression Mean Difference Estimates for Adjusted Models

Covariate	Mean Difference	95% C.I.	P-Value
Treatment Group (CN-105 v Placebo)	XXX	(XXX , XXX)	XXX
Sex (Female v Male)	XXX	(XXX, XXX)	XXX
Age	XXX	(XXX, XXX)	XXX
Race (Reference: White/Caucasian)			XXX
African American/Black	XXX	(XXX, XXX)	XXX
Asian	XXX	(XXX, XXX)	XXX
Other	XXX	(XXX, XXX)	XXX
APOE4 (Carrier v Non-Carrier)	XXX	(XXX, XXX)	XXX
Van Walraven Elixhauser Comorbidity Index	XXX	(XXX, XXX)	XXX
Surgery Duration (per hour)	XXX	(XXX, XXX)	XXX
Estimated Blood Loss (per 100 ml)	XXX	(XXX, XXX)	XXX

Additional exploratory sensitivity analyses will be conducted for primary and secondary outcomes investigating potential interaction effects or important subgroups. These additional exploratory analyses will be performed in case there are interaction effects between CN-105 (whether beneficial or harmful) and sex, baseline/preoperative cognitive status, Racial groups, or APOE genotype.
