# Supplementary material for: Apolipoprotein E Mimetic Peptide CN-105 and Postoperative Delirium in Older Patients: The Phase 2 MARBLE Randomized Clinical Trial
Source: JAMA Netw Open. 2026 Apr 3;9(4):e262289. doi: 10.1001/jamanetworkopen.2026.2289 (PMC13049496; doi:10.1001/jamanetworkopen.2026.2289)
Supplement: Supplement 2. — eMethods eResults eTable 1. Additional Preoperative Patient Characteristics (Intention to Treat) eTable 2. Multivariable Logistic Regression Results with Firth Correction for Incidence of Grade 2 or Higher Adverse Events eTable 3. Multivariable Negative Binomial Regression Results for the Number of Grade 2 or Higher Adverse Events per Patient eTable 4. Summary of Adverse Events by Surgical Service for the CTCAEv5.0-Classified Body Systems, Renal/Urinary and Nervous System eTable 5. Summary of Adverse Events by CTCAEv5.0-Classified Body System (Renal/Urinary and Nervous System) and Surgical Service eTable 6. Summary of Adverse Events by CTCAEv5.0-Classified Body System (Renal/Urinary and Nervous System) and CN-105 Dose Level, for Rates > 0 eTable 7. Summary of Severe Adverse Event Rates by CTCAEv5.0-Classified Body System, for Systems with ≥1 SAE eTable 8. Multivariable Logistic Regression Model with Firth Correction for Delirium Incidence eTable 9. Multivariable Proportional Odds Model for Delirium Severity eTable 10. Delirium Incidence by CN-105 Dose Level vs Placebo eTable 11. Delirium Severity by CN-105 Dose Level vs Placebo eTable 12. Effect of CN-105 on Change in Cognitive Domain Scores From Before to 6-Weeks After Surgery eTable 13. Multivariable Linear Regression Model of Preoperative to 6 Week Postoperative Cognitive Change (Continuous Cognitive Index Change) eTable 14. Preoperative to 6 Week Postoperative Cognitive Change (Continuous Cognitive Index Change) by CN-105 Dose Level vs Placebo eTable 15. Preoperative and 24 Hour Postoperative CSF Alzheimer’s Disease-Related Biomarkers (Intention to Treat) eFigure. Box and Whisker Plots of Blood Plasma and Cerebrospinal Fluid (CSF) Levels of CN-105 at 24 Hours After Surgery by Group (Placebo vs CN-105 Dose Levels) eReferences [file jamanetwopen-e262289-s002.pdf]

Supplemental Online Content

Timko NJ, Cooter Wright M, Smith MR, et al; for the MARBLE Study Investigators. The apolipoprotein E mimetic peptide CN-105 and postoperative delirium in older patients: the phase 2 MARBLE randomized clinical trial. *JAMA Netw Open*. 2026;9(4):e262289. doi:10.1001/jamanetworkopen.2026.2289

eMethods

eResults

eTable 1. Additional Preoperative Patient Characteristics (Intention to Treat)

eTable 2. Multivariable Logistic Regression Results with Firth Correction for Incidence of Grade 2 or Higher Adverse Events

eTable 3. Multivariable Negative Binomial Regression Results for the Number of Grade 2 or Higher Adverse Events per Patient

eTable 4. Summary of Adverse Events by Surgical Service for the CTCAEv5.0-Classified Body Systems, Renal/Urinary and Nervous System

eTable 5. Summary of Adverse Events by CTCAEv5.0-Classified Body System (Renal/Urinary and Nervous System) and Surgical Service

eTable 6. Summary of Adverse Events by CTCAEv5.0-Classified Body System (Renal/Urinary and Nervous System) and CN-105 Dose Level, for Rates > 0

eTable 7. Summary of Severe Adverse Event Rates by CTCAEv5.0-Classified Body System, for Systems with ≥1 SAE

eTable 8. Multivariable Logistic Regression Model with Firth Correction for Delirium Incidence

eTable 9. Multivariable Proportional Odds Model for Delirium Severity

eTable 10. Delirium Incidence by CN-105 Dose Level vs Placebo

eTable 11. Delirium Severity by CN-105 Dose Level vs Placebo

eTable 12. Effect of CN-105 on Change in Cognitive Domain Scores From Before to 6-Weeks After Surgery

© 2026 Timko NJ et al. *JAMA Network Open*

eTable 13. Multivariable Linear Regression Model of Preoperative to 6 Week Postoperative Cognitive Change (Continuous Cognitive Index Change)

eTable 14. Preoperative to 6 Week Postoperative Cognitive Change (Continuous Cognitive Index Change) by CN-105 Dose Level vs Placebo

eTable 15. Preoperative and 24 Hour Postoperative CSF Alzheimer's Disease-Related Biomarkers (Intention to Treat)

eFigure. Box and Whisker Plots of Blood Plasma and Cerebrospinal Fluid (CSF) Levels of CN-105 at 24 Hours After Surgery by Group (Placebo vs CN-105 Dose Levels)

eReferences

This supplemental material has been provided by the authors to give readers additional information about their work.

© 2026 Timko NJ et al. *JAMA Network Open*

eMethods

Enrollment Inclusion/Exclusion Criteria

Since CN-105's beneficial effects are not dependent on apolipoprotein E (*APOE*) allele background,[1-6] enrollment was not restricted by *APOE* ε4 allele (*APOE4*) carrier status.

CN-105 or Placebo Preparation and Administration

CN-105, an ApoE mimetic pentapeptide (Ac-VSRRR-NH_2), was manufactured by University of Iowa Pharmaceuticals (Iowa City, IA), supplied as a sterile frozen liquid in 10 mL amber glass vials (CN-105, 50 mg [12.5 mg/mL], and mannitol, 80 mg [20 mg/mL], reconstituted in 4 mL of sterile water for injection), and stored in a -80° C freezer. CN-105 solutions were thawed at ambient temperature, gently mixed, and diluted (based on patient weight and desired dose) in 100 mL normal saline (0.9% Sodium Chloride for Injection, USP) and administered within 24 hours. Placebo was 100 mL normal saline (0.9% Sodium Chloride for Injection, USP). If dosing solutions were prepared more than 1 hour before the planned start of the study drug infusion, the infusion bag was refrigerated at 2 to 8° C. Each dose of CN-105 or placebo was administered intravenously (IV) over 10 minutes using a 1.2-micron in-line filter.

Mannitol was used as a diluent for the CN-105 peptide, in line with prior studies of CN-105 in humans, because mannitol is required for lyophilization of the hygroscopic CN-105 peptide due to its triple arginine moiety.[7,8] In this study, CN-105 stock solutions contained 12.5 mg/mL of CN-105 and 20 mg/mL mannitol dissolved in sterile water for injection, which was subsequently diluted in 100 mL 0.9% normal saline; placebo patients simply received 100 mL 0.9% normal saline. When the **M**odulating **A**poE signaling to **R**educe **B**rain inflammation, de**L**irium and postop**E**rative cognitive dysfunction (MARBLE) study was designed, we selected 0.9% normal saline as the placebo to maintain consistency with all previous and ongoing CN-105 clinical trials in the United States and Singapore.[7,8] For the CN-105 dose-range used here (0.1-1 mg/kg), the CN-105 treated patients therefore received a mannitol dose ranging from 0.16 to 1.6 mg/kg, which is several orders of magnitude lower than the mannitol dose (0.5-2 g/kg or 500 to 2000 mg/kg) used clinically to treat brain edema and elevated intracranial pressure.[9] Thus, the mannitol dose that CN-105 treated patients received in this study was likely too low to be of clinical significance; it is highly unlikely that this small amount of mannitol could explain the lower rate of adverse events seen in the CN-105 vs placebo patients.

Data Safety Monitoring Board

This phase 2 study, MARBLE, was designed to be stopped if the rate of grade 3 or higher severe adverse events (SAEs)[10] in drug-treated patients was >10% and more than three times the rate of SAEs in the placebo-treated group within either the first or second cohorts. Since we anticipated that a large variety of surgical procedures and patient comorbidities would contribute to adverse events in this study, the independent data safety monitoring board (DSMB) was advised to use both this quantitative cutoff and its clinical judgement in determining whether the study should be stopped.

Blood and Cerebrospinal Fluid Sampling

Blood samples were collected by sterile venipuncture, IV line, or central line, and centrifuged to separate plasma from the red cell pellet and buffy coat. Cerebrospinal fluid (CSF) samples were collected by lumbar puncture at the L4-5 spinal level or the next interspace above or below, with a 25 or 27g pencil point needle using a spinal anesthesia kit (Smiths Medical; Dublin, OH), according to our standardized protocol to minimize pain/adverse events (AEs).[11] The first 1 mL CSF was discarded to reduce blood contamination, and then ~12 mL of CSF was

© 2026 Timko NJ et al. *JAMA Network Open*

collected using a fresh 10 mL syringe. CSF and blood samples were processed as described,[12,13] and stored at -80 ºC. CSF was aliquoted in Sarstedt polypropylene microcentrifuge tubes (VWR; Radnor, PA).

CN-105 Blood Plasma and Cerebrospinal Fluid Assays

The Duke Proteomics and Metabolomics Core Facility (DPMCF) developed an LC-MS/MS assay to specifically measure the CN-105 levels with multiple reaction monitoring (MRM), which greatly increases the detection limit and minimizes the signal interference from other compounds.[14] Data was obtained using ultraperformance liquid chromatography-tandem mass spectrometry (UPLC–MS/MS) on a Waters TQ-S mass spectrometer (Waters Corporation, Milford, MA) with calibration curves for CN-105, consistent with the FDA Guidance for Bioanalytical Method Validation.[15]

Plasma and CSF Sample Preparation

Plasma and CSF samples from MARBLE participants were transferred to DPMCF on dry ice. Plasma samples were then thawed on ice, and a 50 µL plasma sample was spiked into the Eppendorf vial and mixed with 200 µL 5% trichloroacetic acid (Sigma-Aldrich, Inc.; St. Louis, MO) and 10 µL of 1 µg/mL heavy labeled CN-105 (custom synthesized by JPT Peptide Technologies GmbH, Berlin, Germany, and stored at -80 °C). The sample was then vortexed and then kept at -20 °C for 20 minutes. Subsequently, the sample was centrifuged for 4 minutes at 20,000 RCF at 5 °C. The entire sample supernatant was then transferred into the well of a 1 mL 96-well NUNC plate (Thermo Scientific; Waltham, MA), which was centrifuged at 800 RCF for 2 minutes at 10 °C before instrument injection.

After thawing CSF samples on ice, a 30 µL CSF sample was spiked into the well of a 1 mL 96-well NUNC plate and mixed with 60 µL 5% trichloroacetic acid and 10 µL 0.5 µg/mL heavy labeled CN-105, vortexed, and then kept at -20 °C for 20 minutes. Subsequently, the plate was centrifuged for 10 minutes at 800 RCF at 10 °C. 70 µL supernatant was transferred into the well of a new 1 mL 96-well NUNC plate and then centrifuged at 800 RCF for 2 minutes at 10 °C before instrument injection.

Preparation of Calibration Standards and Quality Control (QC) Samples

CN-105 was dissolved at 5 mg/mL in water and stored at -20 °C and was used as a standard solution. Two pool samples (from the first ten plasma samples and the first six CSF samples, respectively) were used as study pool quality control (SPQC). SPQC samples, a series of standard solutions and three QC samples (low, medium, and high concentration) were prepared with the same protocol as real samples.

Sample Analysis by UPLC–MS/MS

Samples were analyzed using a Waters TQ-S MS system with Acquity UPLC (Milford, MA). Software Masslyn 4.2 was used for data acquisition. The liquid chromatography separation was performed on an Acquity UPLC (Milford, MA) CSH phenyl-hexyl column (2.1 x 100mm, 1.7 µm) with mobile phase A (0.1% formic acid in water) and mobile phase B (0.1% formic acid in acetonitrile). The flow rate was 0.4 mL/min. The linear gradient was 0 min, 100% A; 1.5 min, 50% A; 2.5-4.0 min, 0% A, 4.1-5.6 min, 100% A. The autosampler was set at 10°C and the column was kept at 45°C. The injection volume was 5 µL.

Mass spectra were acquired under positive electrospray ionization (ESI) with capillary voltage of 0.6 kV, desolvation temperature of 650 °C, source temperature of 150 °C, and a desolvation gas flow of 850 L/hr. CN-105 was quantified with multiple reaction monitoring (MRM) as the detection approach:

© 2026 Timko NJ et al. *JAMA Network Open*

Target MRM transition for CN-105: m/z 357.7 → m/z 349.1 and 336.4

Labeled Internal standard MRM transition for CN-105: m/z 362.8 → m/z 114.0

The retention time was 1.9 minutes. All data was analyzed in Skyline v23.1.0.268 (www.skyline.ms)[16] which includes raw data import, peak integration, and a linear regression fit with $1/x^2$ weighting for the calibration curve. The variation in retention time for the majority of samples was ± 0.05 minutes.

Plasma sample calibration curves included nine different concentration values (1/2/5/20/100/500/2,000/5,000/10,000 ng/mL), and QC samples were 50, 200, and 1,000 ng/mL. CSF sample calibration curves included seven different concentration values (0.5/2/10/50/200/500/1,000 ng/mL) and QC samples are 5, 20, and 100 ng/mL. Each calibrator's residual bias was calculated after regression fit, and any points where the residual fell outside of 15% were removed from the calibration curve equation.

Cerebrospinal Fluid Alzheimer's Disease Biomarker Assays

CSF β-amyloid 42 (Aβ42), total tau (t-tau), and phosphorylated tau 181 (p-tau181) were measured using the Roche Elecsys cobas e601 module (Roche Diagnostics, Basel, Switzerland).[17,18]

Cerebrospinal Fluid Samples and Neuroinflammatory Cytokine Assays

CSF cytokine assays were performed using the Meso Scale Discovery (MSD) multiplex platform (Meso Scale Diagnostics, LLC, Rockville, MD) by technicians blinded to randomization assignment and sample time point. Specifically, IL-6 and IL-8 were measured using the custom human MSD VPLEX Proinflammatory assay, MCP-1 was measured using the VPLEX Chemokine assay, and G-CSF was measured using the UPLEX 8 assay. All assays used a total volume of 200 µL of CSF from each sample tube. CSF was diluted 2-fold for the interleukin-6 (IL-6), IL-8, and monocyte chemoattractant protein-1 (MCP-1) assays, and 4-fold for granulocyte colony stimulating factor (G-CSF) assay. All samples were run in duplicate. A control CSF sample was created by pooling 9 CSF samples from another study[12] and run in duplicate on all MSD assay plates to evaluate inter-assay variability.

VPLEX kits from MSD provide the lower limit of detection (LLOD; concentration of the signal 2.5 standard deviations above background) and upper and lower limits of quantitation (ULOQ and LLOQ; highest and lowest concentration at which the coefficient of variation is <20% and the recovery of each analyte is 80-120% of the known value, respectively) that define the detectable levels of each analyte. Samples with values below the LLOQ were imputed as ½ the LLOQ.

Intra-assay coefficients of variation were calculated as %CV for standard, control, and samples run in duplicate on each plate, and then averaged to a mean %CV. Inter-assay coefficients of variation were calculated as %CV (SD/Mean*100) for pooled control samples run in duplicate on all plates.

Cognitive Testing Battery and Analysis

Cognition was assessed in MARBLE[19] using a standardized cognitive test battery,[20,21] administered by staff trained by a board-certified neuropsychologist (J.N.B.), within 2 months before (baseline/preoperative) and again 6 weeks after surgery. This test battery included the following assessments (Test name—Cognitive function assessed): (1) Brief Visuospatial Memory Test, Revised—Visuospatial learning and recall; (2) Controlled Oral Word Association Test—Verbal fluency and information retrieval; (3) Hopkins Verbal Learning Test, Revised—Auditory learning and verbal recall; (4) Lafeyette Grooved Pegboard Test—Manual dexterity and motor speed; (5) Montreal Cognitive Assessment—Mild cognitive impairment screening; (6) Trail Making Test, Parts A & B—Complex executive functioning skills (e.g., logical task switching); (7) Wechsler Adult Intelligence Scale, 3rd Revision Digit Span Subtest—Immediate auditory-verbal recall and complex attention; (8) Wechsler Adult Intelligence Scale, 3rd

Revision Digit Symbol Coding Subtest—Visual scanning and visuomotor production; and (9) Wechsler Test of Adult Reading—Premorbid intellectual function.

Individual test scores were standardized (via z-score transformation relative to baseline mean and variance) and grouped by a board-certified neuropsychologist (J.N.B.) into four *a priori* defined cognitive domains (Attention, Verbal Memory, Visual Memory, and Executive Functioning/Processing Speed). Domain scores were calculated as the average of the standardized test scores. The mean of these cognitive domain scores yields the global Continuous Cognitive Index (CCI), which is used to quantify overall cognitive function. Thus, CCI change from before to after surgery quantifies the degree of learning/cognitive improvement or cognitive decline.

Since changes in specific cognitive domains have been observed from before to after surgery,[26] we examined the change in each individual cognitive domain scores from before to 6 weeks after surgery between CN-105 vs placebo treated patients. This is the first human study to measure cognitive change between CN-105 vs placebo treated patients; thus, we had no a priori hypothesis about which particular cognitive domain(s) will be affected the most or the least by CN-105 treatment. We examined differences between CN-105 vs placebo treated patients in each of the cognitive domain score changes (from before to after surgery) as an exploratory outcome.

The incidence of postoperative cognitive dysfunction (POCD), also known as neurocognitive disorder-postoperative (NCD-postoperative), mild and/or major,[24,25] between CN-105 vs placebo treated patients was examined as an exploratory outcome. NCD-postoperative, mild, was defined as a 1–2 standard deviation (SD) decrease in score on any one of the four cognitive domain (used in calculating the CCI, as described above) combined with a subjective cognitive complaint. The value for a 1 or 2 SD decrease in any one of the four cognitive domains was defined by the SD in the entire study population at the baseline/preoperative timepoint. Subjective cognitive complaints were assessed using the Cognitive Difficulties Scale,[27] which our group has previously used to examine the association between subjective and objective cognitive deficits after surgery.[28] The Cognitive Difficulties Scale was administered both before and 6 weeks after surgery. We defined a subjective cognitive complaint as any reported subjective cognitive difficulty at the 6-week visit (irrespective of the baseline response). NCD-postoperative, major, was defined as a ≥2 SD decrease in any one of the four cognitive domain factors, combined with a postoperative deficit in ability to perform one or more activities of daily living (ADLs), and a subjective cognitive complaint. Patients' ability to perform ADLs was assessed using the Duke Activity Status Index,[29] which was administered both before and 6 weeks after surgery.

© 2026 Timko NJ et al. *JAMA Network Open*

eResults

Cerebrospinal Fluid Samples and Neuroinflammatory Cytokine Assays

For Figure 4, panels a-d, CSF samples and cytokine data were obtained from 171 participants (45 placebo, 126 CN-105) before surgery and 141 participants (38 placebo, 103 CN-105) at the 24-hour postoperative time point. Thus, data on baseline to 24-hour postoperative CSF cytokine level change was available from 135 (37 placebo, 98 CN-105) participants.

Using VPLEX kits from MSD, the median LLOD (LLOQ, ULOQ) in pg/ml was 0.07 (0.633, 488) for IL-6, 0.03 (0.591, 375) for IL-8, and 0.05 (1.09, 375) for MCP-1. UPLEX assays, e.g., for G-CSF, are not validated and do not have LLOQ or ULOQ values on the MSD platform, though the median LLOD for UPLEX G-CSF was 1.87.pg/ml. The percent of samples below the LLOD was 0% for IL-6, IL-8, and MCP-1, and 2.8 % for G-CSF.

The intra-assay mean (%CV) of control CSF samples (vs. manufacturer reported values, when available) were as follows: IL-6 (4.0% vs. 4.0%), IL-8 (2.2% vs. 3.1%), MCP-1 (3.1% vs. 8.0%), and G-CSF (8.9%). The inter-assay mean (%CV) of control CSF samples (vs manufacturer reported values, when available) were as follows: IL-6 (6.1% vs. 6.4%), IL-8 (3.4% vs. 6.4%), MCP-1 (4.1% vs. 5.8%), and G-CSF (19.3%).

Postoperative Hospital and Intensive Care Unit Length of Stay

In exploratory analyses, postoperative hospital and intensive care unit (ICU) length of stay was evaluated among CN-105 vs placebo patients. The hospital length of stay was a median [IQR] of 1 [1-2] days in the CN-105 group vs 1 [1-3] days in the placebo group (Wilcoxon p=.16). Furthermore, 2 (4.1%) patients in the placebo group and 1 (0.7%) patient in the CN-105 group required ICU admission after surgery, Fisher test p=.17. The ICU lengths of stay were 23 hours and 72 hours in the two placebo patients, and 3 hours in the CN-105 patient.

Incidence and Number of Grade ≥3 Adverse Events

Grade ≥3 AE incidence was 43.8% (N=60/137) in the CN-105 group vs 55.1% (27/49) in the placebo group; χ^2 p=.17. The median number of grade ≥3 AEs per patient in the CN-105 group vs the placebo group was median [IQR], 0 [0,1] vs 1 [0,1], Wilcoxon p=.08.

© 2026 Timko NJ et al. *JAMA Network Open*

eTable 1: Additional Preoperative Patient Characteristics (Intention to Treat)

	Placebo (N=49)	CN-105 (N=137)	Total (N=186)
**Quality of Life Questionnaires**			
DASI[a]	19.8 (16.8)	20.6 (16.1)	20.4 (16.2)
IADL[a]	6.8 (1.8)	7.1 (2.5)	7.0 (2.4)
Mental Abilities[a]	73.4 (19.2)	73.6 (19.1)	73.5 (19.1)
CES-D	9.6 (8.4)	9.2 (7.1)	9.3 (7.4)
STAI	30.3 (8.7)	30.7 (9.5)	30.6 (9.3)
SF-36 General Health	58.3 (9.3)	60.2 (9.8)	59.7 (9.7)
***APOE* Genotype by Allele**[b]			
ε2/ε2	0 (0.0%)	1 (0.7%)	1 (0.5%)
ε2/ε3	1 (2.1%)	11 (8.1%)	12 (6.5%)
ε2/ε4	1 (2.1%)	4 (2.9%)	5 (2.7%)
ε3/ε3	33 (68.8%)	80 (58.8%)	113 (61.4%)
ε3/ε4	12 (25.0%)	37 (27.2%)	49 (26.6%)
ε4/ε4	1 (2.1%)	3 (2.2%)	4 (2.2%)

Data represents mean (SD) or number (%). Quality of life measures in this table were obtained prospectively from questionnaires that patients filled out as part of this study. [a]Quality of life (QOL) test data were not obtained from 10 subjects due to COVID-19 pandemic restrictions. This selection of QOL questionnaires was based on a QOL battery used in prior work at Duke.[30] [b]Apolipoprotein E (*APOE*) genotype was unable to be obtained for 2 subjects. Abbreviation: DASI, Duke Activity Status Index; IADL, Instrumental Activities of Daily Living; CES-D, Center for Epidemiological Studies Depression Scale; STAI, State Trait Anxiety Index; SF-36, 36-Item Short Form Health Survey.

© 2026 Timko NJ et al. *JAMA Network Open*

eTable 2: Multivariable Logistic Regression Results with Firth Correction for Incidence of Grade 2 or Higher Adverse Events

Variable	Odds Ratio (95% CI)	P-Value
CN-105 vs Placebo	0.47 (0.17, 1.25)	.13
Age (per year)	1.07 (0.99, 1.16)	.07
Sex (Female vs Male)	1.68 (0.74, 3.83)	.22
Race		
Black or African American vs White	2.89 (0.46, 18.10)	.14
Other vs White	0.45 (0.10, 2.08)	.13
APOE ε4 Carrier vs Non-Carrier	1.32 (0.58, 3.00)	.52
Elixhauser Van Walraven Score (per point)	1.06 (0.98, 1.15)	.15
EBL (per 100 mL)	1.16 (0.91, 1.49)	.24
Surgery Duration (per hour)	1.08 (0.84, 1.39)	.57

CN-105 (N=137), Placebo (N=49).

© 2026 Timko NJ et al. *JAMA Network Open*

eTable 3: Multivariable Negative Binomial Regression Results for the Number of Grade 2 or Higher Adverse Events per Patient

Variable	Incidence Rate Ratio (95% CI)	P-Value
CN-105 vs Placebo	0.65 (0.47, 0.91)	.01
Age (per year)	1.02 (0.99, 1.05)	.13
Sex (Female vs Male)	1.12 (0.81, 1.54)	.50
Race		
Black or African American vs White	0.72 (0.39, 1.33)	.30
Other vs White	0.36 (0.14, 0.92)	.03
APOE4 Carrier vs Non-Carrier	1.24 (0.89, 1.72)	.20
Elixhauser Van Walraven Score (per point)	1.06 (1.02, 1.09)	.001
Estimated Blood Loss (per 100 mL)	1.05 (0.99, 1.11)	.14
Surgery Duration (per hour)	1.14 (1.04, 1.25)	.006

CN-105 (N=137), Placebo (N=49).

© 2026 Timko NJ et al. *JAMA Network Open*

eTable 4: Summary of Adverse Events by Surgical Service for the CTCAEv5.0–Classified Body Systems, Renal/Urinary and Nervous System

**Surgical Service (Number of Surgeries: N Placebo/N CN-105)**	**Placebo (N=49)**	**CN-105 (N=137)**
**Renal/Urinary Adverse Events**	8 (16.3%)	8 (5.8%)
Thoracic Surgery (3/8)	0 (0)	0 (0)
General Surgery (6/24)	0 (0)	0 (0)
Gynecologic Surgery (4/3)	0 (0)	0 (0)
Orthopedic Surgery (18/59)	3 (6.1%)	5 (3.6%)
Otolaryngologic Surgery (3/6)	1 (2.0%)	0 (0)
Plastic Surgery (1/7)	0 (0)	0 (0)
Urologic Surgery (14/30)	4 (8.2%)	3 (2.2%)
**Nervous System Adverse Events**	8 (16.3%)	9 (6.6%)
Thoracic Surgery (3/8)	0 (0)	2 (1.5%)
General Surgery (6/24)	0 (0)	1 (4.2%)
Gynecologic Surgery (4/3)	0 (0)	0 (0)
Orthopedic Surgery (18/59)	6 (12.2%)	3 (2.2%)
Otolaryngologic Surgery (3/6)	2 (6.1%)	0 (0.0%)
Plastic Surgery (1/7)	0 (0.0%)	1 (0.7%)
Urologic Surgery (14/30)	0 (0.0%)	2 (1.5%)

Data represent number (%). The surgical service column presents the number of surgeries (N Placebo/N CN-105) by surgical service. The percentage are column-wise and represent the number of patients who underwent each surgery type and had an AE, divided by the total number of placebo or CN-105 patients.

© 2026 Timko NJ et al. *JAMA Network Open*

eTable 5: Summary of Adverse Events by CTCAEv5.0–Classified Body System (Renal/Urinary and Nervous System) and Surgical Service

	Placebo (N=49)	CN-105 (N=137)	Total (N=186)	Surgical Service
**Renal/Urinary Adverse Events**	8 (16.3%)	8 (5.8%)	16 (8.6%)	8 Orthopedic surgery (3 Placebo, 5 CN-105), 1 Otolaryngologic surgery (Placebo), and 7 Urologic surgery (4 Placebo, 3 CN-105)
Urinary tract obstruction	0 (0.0%)	2 (1.5%)	2 (1.1%)	2 Orthopedic surgery (CN-105)
Urinary retention	3 (6.1%)	5 (3.6%)	8 (4.3%)	7 Orthopedic surgery (3 Placebo, 4 CN-105), 1 Urologic surgery (CN-105)
Hematuria	1 (2.0%)	1 (0.7%)	2 (1.1%)	2 Urologic surgery
Cystitis noninfective (vs prostatitis, dysuria, proteinuria)	0 (0.0%)	2 (1.5%)	2 (1.5%)	1 Orthopedic surgery, 1 Urologic surgery
Urinary urgency	1 (2.0%)	0 (0.0%)	1 (0.5%)	Urologic surgery
Bladder spasm	1 (2.0%)	0 (0.0%)	1 (0.5%)	Urologic surgery
Urinary incontinence	3 (6.1%)	0 (0.0%)	3 (1.6%)	1 Otolaryngologic surgery, 2 Urologic surgery
**Nervous System Adverse Events**	8 (16.3%)	9 (6.6%)	17 (9.1%)	2 Thoracic surgery (2 CN-105), 1 General surgery (CN-105), 9 Orthopedic surgery (6 Placebo, 3 CN-105), 2 Otolaryngologic surgery (2 Placebo), 1 Plastic surgery (CN-105), and 2 Urologic surgery (2 CN-105)
Syncope	3 (6.1%)	1 (0.7%)	4 (2.2%)	2 Orthopedic surgery (2 Placebo), 1 Otolaryngologic surgery (Placebo), 1 Urologic surgery (CN-105)
Radiculitis	0 (0.0%)	1 (0.7%)	1 (0.5%)	Urologic surgery
Headache	1 (2.0%)	3 (2.2%)	4 (2.2%)	2 Thoracic surgery (2 CN-105), 1 Orthopedic surgery (Placebo), 1 Plastic surgery (CN-105)
Dizziness	5 (10.2%)	3 (2.2%)	8 (4.3%)	7 Orthopedic surgery (4 Placebo, 3 CN-105), 1 Otolaryngologic surgery (Placebo)
Vasovagal reaction	0 (0.0%)	2 (1.5%)	2 (1.1%)	1 General surgery, 1 Plastic surgery
Cerebrospinal fluid leakage	1 (2.0%)	0 (0.0%)	1 (0.5%)	1 Orthopedic surgery

Data represent number (%).

© 2026 Timko NJ et al. *JAMA Network Open*

eTable 6: Summary of Adverse Events by CTCAEv5.0–Classified Body System (Renal/Urinary and Nervous System) and CN-105 Dose Level, for Rates > 0

	Placebo (N=49)	0.1 mg/kg CN-105 (N=48)	0.5 mg/kg CN-105 (N=48)	1 mg/kg CN-105 (N=41)
**Renal/Urinary Adverse Events**	8 (16.3%)	5 (10.4%)	1 (2.1%)	2 (4.9%)
Urinary tract obstruction	0 (0.0%)	1 (2.1%)	1 (2.1%)	0 (0.0%)
Urinary retention	3 (6.1%)	2 (4.2%)	1 (2.1%)	2 (4.9%)
Hematuria	1 (2.0%)	1 (2.1%)	0 (0.0%)	0 (0.0%)
Cystitis–noninfective (vs prostatitis, dysuria, proteinuria)	0 (0.0%)	2 (4.2%)	0 (0.0%)	0 (0.0%)
Urinary urgency	1 (2.0%)	0 (0.0%)	0 (0.0%)	0 (0.0%)
Bladder spasm	1 (2.0%)	0 (0.0%)	0 (0.0%)	0 (0.0%)
Urinary incontinence	3 (6.1%)	0 (0.0%)	0 (0.0%)	0 (0.0%)
**Nervous System Adverse Events**	8 (16.3%)	4 (8.3%)	2 (4.2%)	3 (7.3%)
Syncope	3 (6.1%)	1 (2.1%)	0 (0.0%)	0 (0.0%)
Radiculitis	0 (0.0%)	1 (2.1%)	0 (0.0%)	0 (0.0%)
Headache	1 (2.0%)	2 (4.2%)	1 (2.1%)	0 (0.0%)
Dizziness	5 (10.2%)	0 (0.0%)	1 (2.1%)	2 (4.9%)
Vasovagal reaction	0 (0.0%)	1 (2.1%)	0 (0.0%)	1 (2.4%)
Cerebrospinal fluid leakage	1 (2.0%)	0 (0.0%)	0 (0.0%)	0 (0.0%)

Data represent number (%).

© 2026 Timko NJ et al. *JAMA Network Open*

eTable 7: Summary of Severe Adverse Event Rates by CTCAEv5.0–Classified Body System, for Systems with ≥ 1 SAE

Body System	Placebo (N=49)	CN-105 (N=137)	Total (N=186)	RR [95% CI]	P-Value	Adjusted P-Value
Vascular Disorders	4 (8.2%)	1 (0.7%)	5 (2.7%)	0.09 [0.01-0.78]	.02	.13
Blood and Lymphatic System	3 (6.1%)	1 (0.7%)	4 (2.2%)	0.12 [0.01, 1.12]	.06	.29
Metabolism and Nutrition	0 (0.0%)	1 (0.7%)	1 (0.5%)	–	–	–
Gastrointestinal	2 (4.1%)	4 (2.9%)	6 (3.2%)	0.72 [0.14-3.78]	.66	>.99
Infections and Infestations	1 (2.0%)	0 (0.0%)	1 (0.5%)	–	–	–
Injury, Poisoning, Procedural	1 (2.0%)	2 (1.5%)	3 (1.6%)	0.72 [0.07, 7.72]	>.99	>.99
General Disorders	1 (2.0%)	0 (0.0%)	1 (0.5%)	–	–	–
Respiratory, Thoracic and Mediastinal	4 (8.2%)	1 (0.7%)	5 (2.7%)	0.09 [0.01-0.78]	.02	.13
Renal and Urinary	0 (0.0%)	1 (0.7%)	1 (0.5%)	–	–	–
Nervous System	1 (2.0%)	0 (0.0%)	1 (0.5%)	–	–	–
Neoplasms	0 (0.0%)	1 (0.7%)	1 (0.5%)	–	–	–
Surgical and Medical Procedures	2 (4.1%)	1 (0.7%)	3 (1.6%)	0.18 [0.02, 1.93]	.17	.68
Psychiatric Disorders	0 (0.0%)	1 (0.7%)	1 (0.5%)	–	–	
Cardiac Disorders	1 (2.0%)	1 (0.7%)	2 (1.1%)	0.36 [0.02, 5.61]	.46	>.99
Skin and Subcutaneous Tissue	1 (2.0%)	0 (0.0%)	1 (0.5%)	–	–	–

Data represent number (%) or relative risk [95% confidence interval]. P-values by Fisher's Exact Test. Relative risk (RR) and P-values reported in body systems with >0 events in both groups. Adjusted P-values account for multiple testing using the Holm method. Abbreviation: SAE, severe adverse event; RR, relative risk; CI, confidence interval.

© 2026 Timko NJ et al. *JAMA Network Open*

eTable 8: Multivariable Logistic Regression Model with Firth Correction for Delirium Incidence

Variable	Odds Ratio (95% CI)	P-Value
CN-105 vs Placebo	0.61 (0.27, 1.37)	.23
Age (per year)	1.09 (1.02, 1.17)	.02
Sex (Female vs Male)	0.83 (0.37, 1.85)	.64
Race		
Black or African American vs White	2.42 (0.72, 8.13)	.43
Other vs White	1.88 (0.36, 9.73)	.83
*APOE*4 Carrier vs Non-Carrier	1.94 (0.88, 4.27)	.10
Elixhauser Van Walraven Score (per point)	1.05 (0.97, 1.14)	.20
Estimated blood loss (per 100 mL)	0.99 (0.83, 1.18)	.88
Surgery Duration (per hour)	0.82 (0.61, 1.09)	.17

CN-105 (N=137), Placebo (N=49). Abbreviation: CI, confidence interval.

© 2026 Timko NJ et al. *JAMA Network Open*

eTable 9: Multivariable Proportional Odds Model for Delirium Severity

Variable	Odds Ratio (95% CI)	P-Value
CN-105 vs Placebo	0.69 (0.37, 1.28)	.24
Age (per year)	1.11 (1.06, 1.17)	<.001
Sex (Female vs Male)	0.65 (0.36, 1.16)	.15
Race		
Black or African American vs White	2.10 (0.77, 5.70)	.15
Other vs White	0.38 (0.10, 148)	.17
*APOE*4 Carrier vs Non-Carrier	2.21 (1.23, 3.99)	.008
Elixhauser Van Walraven Score (per point)	1.04 (0.98, 1.10)	.23
Estimated blood loss (per 100 mL)	1.05 (0.93, 1.20)	.40
Surgery Duration (per hour)	1.00 (0.84, 1.19)	.98

CN-105 (N=137), Placebo (N=49). Abbreviation: CI, confidence interval.

© 2026 Timko NJ et al. *JAMA Network Open*

eTable 10: Delirium Incidence by CN-105 Dose Level vs Placebo

	No Delirium	Yes Delirium	Univariable Logistic Regression Odds Ratio (95% CI)	P-Value
Placebo	36 (73.5%)	13 (26.5%)	Reference	Reference
0.1 mg/Kg	41 (89.1%)	5 (10.9%)	0.34 (0.11, 1.04)	.06
0.5 mg/Kg	36 (75.0%)	12 (25.0%)	0.92 (0.37, 2.29)	.86
1.0 mg/Kg	32 (78.1%)	9 (22.0%)	0.78 (0.29, 2.06)	.62

Data represent number (%). CN-105 (N=137), Placebo (N=49). Abbreviation: CI, confidence interval.

© 2026 Timko NJ et al. *JAMA Network Open*

eTable 11: Delirium Severity by CN-105 Dose Level vs Placebo

	Median [Q1, Q3]	Univariable Proportional Odds Model Odds Ratio (95% CI)	P-Value
Placebo	2 [1, 2]	Reference	Reference
0.1 mg/Kg	1 [0, 2]	0.50 (0.24, 1.04)	.06
0.5 mg/Kg	1 [1, 2]	0.84 (0.41, 1.73)	.64
1.0 mg/Kg	1 [1, 2]	0.68 (0.32, 1.44)	.31

Abbreviation: CI, confidence interval.

© 2026 Timko NJ et al. *JAMA Network Open*

eTable 12: Effect of CN-105 on Change in Cognitive Domain Scores From Before to 6-Weeks After Surgery

	Placebo (N=36)	CN-105 (N=109)	P-Value
Global Cognitive Change[a]	0.08 (0.33)	0.06 (0.34)	.80
Attention	0.08 (0.74)	-0.02 (0.64)	.44
Verbal memory	0.01 (0.66)	0.06 (0.65)	.70
Visual memory	0.24 (0.64)	0.17 (0.74)	.57
Executive Functioning / Processing Speed[a]	0.001 (0.26)	0.04 (0.41)	.49

Data represents mean (SD). There were 143/186 (77%) subjects who completed cognitive testing at baseline and six weeks, of which 108 subjects were in the CN-105 group and 35 subjects were in the placebo group. P-values are from 2-group t-tests.
[a]Missing for 1 placebo subject and 1 CN-105 subject.

© 2026 Timko NJ et al. *JAMA Network Open*

eTable 13: Multivariable Linear Regression Model of Preoperative to 6 Week Postoperative Cognitive Change (Continuous Cognitive Index Change)

Variable	Mean Difference (95% CI)	P-Value
CN-105 vs Placebo	-0.04 (-0.18, 0.09)	.53
Age (per year)	-0.01 (-0.02, 0.00)	.21
Sex (Female vs Male)	0.03 (-0.10, 0.15)	.65
Race		
Black or African American vs White	0.08 (-0.13, 0.30)	.46
Other vs White	0.02 (-0.29, 0.33)	.89
APOE4 Carrier vs Non-Carrier	0.01 (-0.12, 0.13)	.90
Elixhauser Van Walraven Score (per point)	0.00 (-0.01, 0.01)	.92
Estimated blood loss (per 100 mL)	-0.01 (-0.04, 0.02)	.55
Surgery Duration (per hour)	-0.02 (-0.06, 0.01)	.21

There were 143/186 (77%) subjects who completed cognitive testing at baseline and six weeks, of which 108 subjects were in the CN-105 group and 35 subjects were in the placebo group.

© 2026 Timko NJ et al. *JAMA Network Open*

eTable 14: Preoperative to 6 Week Postoperative Cognitive Change (Continuous Cognitive Index Change) by CN-105 Dose Level vs Placebo

	Mean (SD)	$\hat{\beta}$ (95% CI)	P-Value
Placebo (N=35)	0.08 (0.33)	Reference	Reference
0.1 mg/kg CN-105 (N=31)	-0.02 (0.33)	-0.09 (-0.25, 0.07)	.25
0.5 mg/kg CN-105 (N=38)	0.01 (0.31)	-0.07 (-0.22, 0.08)	.38
1.0 mg/kg CN-105 (N=39)	0.17 (0.35)	0.10 (-0.06, 0.25)	.22

Data in the table above is from only those patients who completed both baseline and six-week postoperative cognitive testing. Individual CN-105 dose levels had no effect on postoperative CCI change

© 2026 Timko NJ et al. *JAMA Network Open*

eTable 15: Preoperative and 24 Hour Postoperative CSF Alzheimer's Disease-Related Biomarkers (Intention to Treat)[a]

	Placebo (N=49)	CN-105 (N=137)	Total (N=186)	P-Value
**Aβ42 Positive Status[b]**				
Baseline/Preoperative	10 (21.7%)	37 (28.7%)	47 (26.9%)	.36
**Aβ42 or t-tau Positive Status[b]**				
Baseline/Preoperative	12 (26.1%)	48 (37.2%)	60 (34.3%)	.17
**t-tau (pg/mL)[b]**				
Baseline/Preoperative	201.1 [168.3, 235.0]	206.0 [156.0, 264.0]	203.6 [158.4, 259.7]	.98
24 Hour	190.0 [159.9, 233.0]	197.8 [150.8, 262]	197.3 [151.5, 251.9]	.70
24 Hour Change	-9.1 [-24.0, 2.0]	-7.0 [-15.7, 3.7]	-7.1 [-18.0, 2.5]	.30
**p-tau181 (pg/mL)[b]**				
Baseline/Preoperative	16.6 [14.1, 20.9]	17.4 [12.9, 22.4]	17.4 [13.3, 22.2]	.89
24 Hour	15.8 [13.2, 21.2]	16.9 [12.8, 22.9]	16.5 [12.9, 22.6]	.71
24 Hour Change	-0.6 [-1.9, 0.1]	-0.2 [-1.2, 0.4]	-0.4 [-1.3, 0.4]	.17
**Aβ42 (pg/mL)[b]**				
Baseline/Preoperative	1350 [1064, 1700]	1287 [976, 1700]	1295 [980, 1700]	.83
24 Hour	1321 [982, 1551]	1256 [980, 1700]	1256 [981, 1691]	.72
24 Hour Change	-41 [-160, 0]	0 [-82, 44]	-2 [-111, 39]	.10
**t-tau/Aβ42[b]**				
Baseline/Preoperative	0.14 [0.13, 0.18]	0.14 [0.12, 0.19]	0.14 [0.12, 0.19]	.95
24 Hour	0.14 [0.13, 0.18]	0.15 [0.12, 0.18]	0.15 [0.13, 0.18]	.74
24 Hour Change	-0.001 [-0.01, 0.01]	0.002 [-0.01, 0.01]	0.001 [-0.01, 0.01]	.60
**p-tau181/Aβ42[b]**				
Baseline/Preoperative	0.01 [0.01, 0.02]	0.01 [0.01, 0.02]	0.01 [0.01, 0.02]	.94
24 Hour	0.01 [0.01, 0.01]	0.01 [0.01, 0.02]	0.01 [0.01, 0.02]	.68
24 Hour Change	-0.0002 [-0.001, 0.001]	-0.00005 [-0.001, 0.001]	-0.0001 [-0.001, 0.001]	.72

Data represents median [IQR] or number (%). 24 Hour Change represents 24 hour postoperative CSF AD biomarker levels minus baseline CSF AD biomarker levels. [a]Alzheimer's disease-related biomarkers were available for n=175 of 186 study participants at baseline/preoperative and n=140 of 186 participants at 24 hours; n=138 of 186 participants have both baseline/preoperative and 24 hours, due to patients not having CSF collected via lumbar puncture from one or the other time point (or both time points). [b]Alzheimer's disease-related biomarker data was not obtained on 11 patients who did not have CSF samples available. t-tau was defined as positive if t-tau >300 pg/mL;[17] Aβ42 was defined as positive if Aβ42 ≤1000 pg/mL.[18] Abbreviation: CSF, cerebrospinal fluid, t-tau, total tau; p-tau181, phosphorylated tau 181; Aβ42, β-amyloid 42.

© 2026 Timko NJ et al. *JAMA Network Open*

eFigure: Box and Whisker Plots of Blood Plasma and Cerebrospinal Fluid (CSF) Levels of CN-105 at 24 Hours After Surgery by Group (Placebo vs CN-105 Dose Levels)

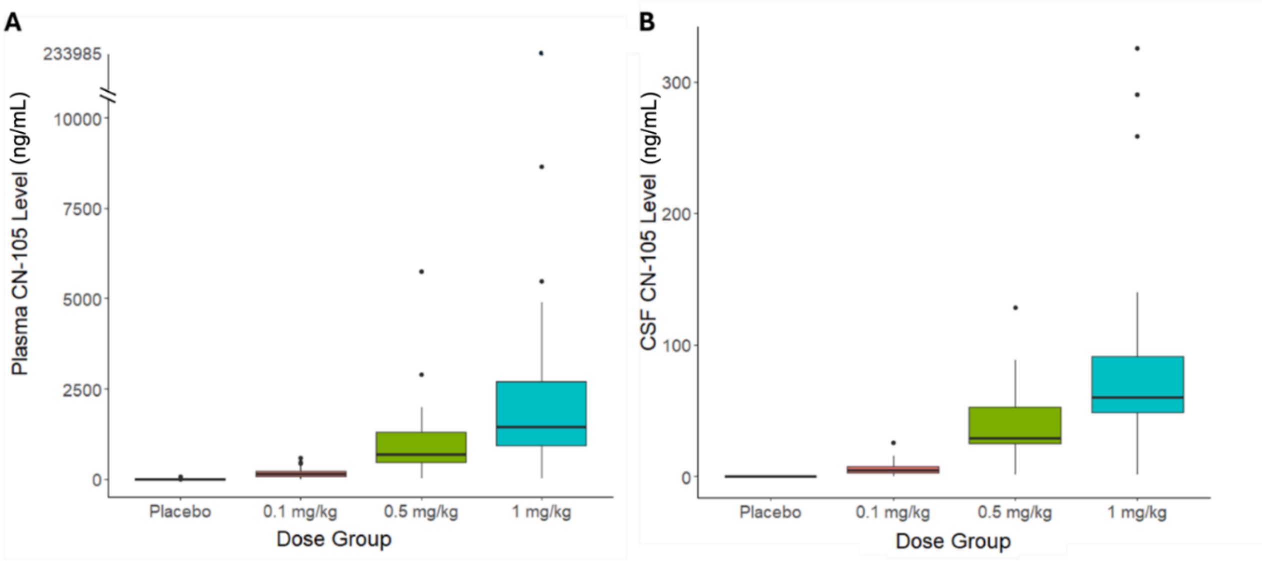

The dark black line within each box represents the group median, the upper and lower edges of each box represent the boundaries between the first and second quartiles, and the third and fourth quartiles, respectively. The vertical lines represent 1.5 times the interquartile range; data points beyond the vertical lines represent individual outliers.

© 2026 Timko NJ et al. *JAMA Network Open*

eReferences

1. Lei B, James ML, Liu J, et al. Neuroprotective pentapeptide CN-105 improves functional and histological outcomes in a murine model of intracerebral hemorrhage. *Sci Rep*. 2016;6:34834.
2. Liu J, Zhou G, Kolls BJ, et al. Apolipoprotein E mimetic peptide CN-105 improves outcome in a murine model of SAH. *Stroke Vasc Neurol*. 2018;3(4):222-230.
3. Laskowitz DT, Wang H, Chen T, et al. Neuroprotective pentapeptide CN-105 is associated with reduced sterile inflammation and improved functional outcomes in a traumatic brain injury murine model. *Sci Rep*. 2017;7:46461.
4. Van Wyck D, Kolls BJ, Wang H, Cantillana V, Maughan M, Laskowitz DT. Prophylactic treatment with CN-105 improves functional outcomes in a murine model of closed head injury. *Exp Brain Res*. 2022;240(9):2413-2423.
5. Wang H, Faw TD, Lin Y, et al. Neuroprotective Pentapeptide, CN-105, Improves Outcomes in Translational Models of Intracerebral Hemorrhage. *Neurocrit Care*. 2021;35(2):441-450.
6. Tu TM, Kolls BJ, Soderblom EJ, et al. Apolipoprotein E mimetic peptide, CN-105, improves outcomes in ischemic stroke. *Ann Clin Transl Neurol*. 2017;4(4):246-265.
7. Guptill JT, Raja SM, Boakye-Agyeman F, et al. Phase 1 Randomized, Double-Blind, Placebo-Controlled Study to Determine the Safety, Tolerability, and Pharmacokinetics of a Single Escalating Dose and Repeated Doses of CN-105 in Healthy Adult Subjects. *J Clin Pharmacol*. 2017;57(6):770-776.
8. Li S, Wangqin R, Meng X, et al. Tolerability and Pharmacokinetics of Single Escalating and Repeated Doses of CN-105 in Healthy Participants. *Clin Ther*. 2022;44(5):744-754.
9. Kim JH, Jeong H, Choo YH, et al. Optimizing Mannitol Use in Managing Increased Intracranial Pressure: A Comprehensive Review of Recent Research and Clinical Experiences. *Korean J Neurotrauma*. 2023;19(2):162-176.
10. Common Terminology Criteria for Adverse Events (CTCAE) Common Terminology Criteria for Adverse Events (CTCAE) v5.0. Accessed 11/18/2025, 2025. https://ctep.cancer.gov/protocoldevelopment/electronic_applications/docs/CTCAE_v5_Quick_Reference_5x7.pdf
11. Nobuhara CK, Bullock WM, Bunning T, et al. A protocol to reduce self-reported pain scores and adverse events following lumbar punctures in older adults. *J Neurol*. 2020;267(7):2002-2006.
12. Berger M, Browndyke JN, Cooter Wright M, et al. Postoperative changes in cognition and cerebrospinal fluid neurodegenerative disease biomarkers. *Ann Clin Transl Neurol*. 2022;9(2):155-170.
13. Berger M, Murdoch DM, Staats JS, et al. Flow Cytometry Characterization of Cerebrospinal Fluid Monocytes in Patients With Postoperative Cognitive Dysfunction: A Pilot Study. *Anesth Analg*. 2019;129(5):e150-e154.
14. Kitteringham NR, Jenkins RE, Lane CS, Elliott VL, Park BK. Multiple reaction monitoring for quantitative biomarker analysis in proteomics and metabolomics. *J Chromatogr B*. 2009;877(13):1229-1239.
15. Bioanalytical Method Validation Guidance for Industry. Accessed 09/13/2024, https://www.fda.gov/regulatory-information/search-fda-guidance-documents/bioanalytical-method-validation-guidance-industry
16. Skyline. Accessed November 20, 2024. www.skyline.ms
17. Blennow K, Shaw LM, Stomrud E, et al. Predicting clinical decline and conversion to Alzheimer's disease or dementia using novel Elecsys Abeta(1-42), pTau and tTau CSF immunoassays. *Sci Rep*. 2019;9(1):19024.
18. Elecsys® β-Amyloid (1-42) CSF II. Accessed 11/30/2024, https://diagnostics.roche.com/global/en/products/lab/elecsys-beta-amyloid-1-42-csf-ii-pid00000063.html#productSpecs
19. VanDusen KW, Eleswarpu S, Moretti EW, et al. The MARBLE Study Protocol: Modulating ApoE Signaling to Reduce Brain Inflammation, DeLirium, and PostopErative Cognitive Dysfunction. *J Alzheimers Dis*. 2020;75(4):1319-1328.
20. Newman MF, Kirchner JL, Phillips-Bute B, et al. Longitudinal assessment of neurocognitive function after coronary-artery bypass surgery. *N Engl J Med*. 2001;344(6):395-402.
21. Mathew JP, White WD, Schinderle DB, et al. Intraoperative magnesium administration does not improve neurocognitive function after cardiac surgery. *Stroke*. 2013;44(12):3407-3413.

© 2026 Timko NJ et al. *JAMA Network Open*

22. McDonagh DL, Mathew JP, White WD, et al. Cognitive function after major noncardiac surgery, apolipoprotein E4 genotype, and biomarkers of brain injury. *Anesthesiology*. 2010;112(4):852-859.
23. Giattino CM, Gardner JE, Sbahi FM, et al. Intraoperative Frontal Alpha-Band Power Correlates with Preoperative Neurocognitive Function in Older Adults. *Front Syst Neurosci*. 2017;11:24.
24. Evered L, Silbert B, Knopman DS, et al. Recommendations for the nomenclature of cognitive change associated with anaesthesia and surgery-2018. *Br J Anaesth*. 2018;121(5):1005-1012.
25. Barnett AG, van der Pols JC, Dobson AJ. Regression to the mean: what it is and how to deal with it. *Int J Epidemiol*. 2005;34(1):215-220.
26. Price CC, Tanner JJ, Schmalfuss I, et al. A pilot study evaluating presurgery neuroanatomical biomarkers for postoperative cognitive decline after total knee arthroplasty in older adults. *Anesthesiology*. 2014;120(3):601-613.
27. McNair D, RJ K. *Assessment in geriatric psychopharmacology (Chapter: Self-Assessment of Cognitive Deficits; The Cognitive Difficulties Scale)*. Mark Powley Associates; 1983.
28. Phillips-Bute B, Mathew JP, Blumenthal JA, et al. Association of neurocognitive function and quality of life 1 year after coronary artery bypass graft (CABG) surgery. *Psychosom Med*. 2006;68(3):369-375.
29. Hlatky MA, Boineau RE, Higginbotham MB, et al. A brief self-administered questionnaire to determine functional capacity (the Duke Activity Status Index). *Am J Cardiol*. 1989;64(10):651-654.
30. Newman MF, Grocott HP, Mathew JP, et al. Report of the substudy assessing the impact of neurocognitive function on quality of life 5 years after cardiac surgery. *Stroke*. 2001;32(12):2874-2881.

© 2026 Timko NJ et al. *JAMA Network Open*
