# Supplementary material for: Apolipoprotein E Mimetic Peptide CN-105 and Postoperative Delirium in Older Patients: The Phase 2 MARBLE Randomized Clinical Trial
Source: JAMA Netw Open. 2026 Apr 3;9(4):e262289. doi: 10.1001/jamanetworkopen.2026.2289 (PMC13049496; doi:10.1001/jamanetworkopen.2026.2289)
Supplement: Supplement 3. — Nonauthor Collaborators [file jamanetwopen-e262289-s003.pdf]

Supplemental Online Content: Nonauthor Collaborators
*First name, last name, and suffix (if applicable) are required and will appear in PubMed.

*Group Name(s): MARBLE Study Investigators							
***First Name and Middle Initial(s)**	***Last Name**	***Suffix (eg, Jr, III)**	Academic Degrees	Institution	Location (city, state/province, country)	Role or Contribution, eg, chair, principal investigator	Group (if more than 1 Group listed in the byline) and/or Subgroup (eg, Steering Committee)
Michael R.	Abern		MD	Department of Urology, Duke University School of Medicine	Durham, North Carolina, USA		
Leah C.	Acker		MD, PhD	Department of Anesthesiology, Duke University School of Medicine; Department of Neurobiology, Duke University School of Medicine	Durham, North Carolina, USA		
Samuel B.	Adams		MD	Department of Orthopaedic Surgery, Duke University School of Medicine	Durham, North Carolina, USA		
Peter J.	Allen		MD	Department of Surgery, Duke University School of Medicine	Durham, North Carolina, USA		
Cindy L.	Amundsen		MD	Department of Obstetrics and Gynecology, Duke University School of Medicine	Durham, North Carolina, USA		
Oke A.	Anakwenze		MD, MBA	Department of Orthopaedic Surgery, Duke University School of Medicine	Durham, North Carolina, USA		
Pallavi	Avasarala		BA	Department of Anesthesiology, Duke University School of Medicine	Durham, North Carolina, USA		
Matthew D.	Barber		MD, MHS	Department of Obstetrics and Gynecology, Duke University School of Medicine	Durham, North Carolina, USA		
Andrew	Berchuck		MD	Department of Obstetrics and Gynecology, Duke University School of Medicine	Durham, North Carolina, USA		
Daniel G.	Blazer	III	MD	Department of Surgery, Duke University School of Medicine	Durham, North Carolina, USA		

Supplemental Online Content: Nonauthor Collaborators
*First name, last name, and suffix (if applicable) are required and will appear in PubMed.

***First Name and Middle Initial(s)**	***Last Name**	***Suffix (eg, Jr, III)**	Academic Degrees	Institution	Location (city, state/province, country)	Role or Contribution, eg, chair, principal investigator	Group (if more than 1 Group listed in the byline) and/or Subgroup (eg, Steering Committee)
Rachele	Brassard		BA	Department of Anesthesiology, Duke University School of Medicine	Durham, North Carolina, USA		
Brian E.	Brigman		MD, PhD	Department of Orthopaedic Surgery, Duke University School of Medicine	Durham, North Carolina, USA		
W.M.	Bullock		MD, PhD	Department of Anesthesiology, Duke University School of Medicine	Durham, North Carolina, USA		
Roberto	Cabeza		PhD	Department of Psychiatry and Behavioral Sciences, Duke University Medical Center; Department of Psychology and Neuroscience, Duke University; Center for Cognitive Neuroscience, Duke University Medical Center	Durham, North Carolina, USA		
Morgan A.	Caldwell		BS	Department of Anesthesiology, Duke University School of Medicine	Durham, North Carolina, USA		
Soren K.	Christensen			Department of Neurology, Duke University School of Medicine	Durham, North Carolina, USA		
Catharina C.	Eisdorfer		MR	Department of Anesthesiology, Duke University School of Medicine	Durham, North Carolina, USA		
Lori	Cole-Freiburger		BA	Department of Anesthesiology, Duke University School of Medicine	Durham, North Carolina, USA		

Supplemental Online Content: Nonauthor Collaborators
*First name, last name, and suffix (if applicable) are required and will appear in PubMed.

*First Name and Middle Initial(s)	*Last Name	*Suffix (eg, Jr, III)	Academic Degrees	Institution	Location (city, state/province, country)	Role or Contribution, eg, chair, principal investigator	Group (if more than 1 Group listed in the byline) and/or Subgroup (eg, Steering Committee)
Brian J.	Colin		MD	Department of Anesthesiology, Duke University School of Medicine	Durham, North Carolina, USA		
Mitchell W.	Cox		MD	Department of Surgery, Duke University School of Medicine	Durham, North Carolina, USA		
Donna M.	Crabtree		PhD	Duke Office of Clinical Research, Duke University	Durham, North Carolina, USA		
Thomas A.	D’Amico		MD	Division of Cardiovascular and Thoracic Surgery, Department of Surgery, Duke University School of Medicine	Durham, North Carolina, USA		
Brittany A.	Davidson		MD	Department of Obstetrics and Gynecology, Duke University School of Medicine	Durham, North Carolina, USA		
James K.	DeOrio		MD	Department of Orthopaedic Surgery, Duke University School of Medicine	Durham, North Carolina, USA		
Mark E.	Easley		MD	Department of Orthopaedic Surgery, Duke University School of Medicine	Durham, North Carolina, USA		
Clay	Elliott		MS	Department of Anesthesiology, Duke University School of Medicine	Durham, North Carolina, USA		
Detlev	Erdmann		MD, PhD, MHSc	Department of Surgery, Duke University School of Medicine	Durham, North Carolina, USA		
Melissa M.	Erickson		MD	Department of Orthopaedic Surgery, Duke University School of Medicine	Durham, North Carolina, USA		
Alisan	Fathalizadeh		MD	Department of Surgery, Duke University School of Medicine	Durham, North Carolina, USA		

Supplemental Online Content: Nonauthor Collaborators
*First name, last name, and suffix (if applicable) are required and will appear in PubMed.

***First Name and Middle Initial(s)**	***Last Name**	***Suffix (eg, Jr, III)**	Academic Degrees	Institution	Location (city, state/province, country)	Role or Contribution, eg, chair, principal investigator	Group (if more than 1 Group listed in the byline) and/or Subgroup (eg, Steering Committee)
Michael N.	Ferrandino		MD	Department of Surgery, Duke University School of Medicine	Durham, North Carolina, USA		
Robert D.	Fitch		MD	Department of Orthopaedic Surgery, Duke University School of Medicine	Durham, North Carolina, USA		
Howard W.	Francis		MD, MBA	Department of Head and Neck Surgery & Communication Sciences, Duke University School of Medicine	Durham, North Carolina, USA		
Matthew	Fuller		MS	Department of Anesthesiology, Duke University School of Medicine	Durham, North Carolina, USA		
John C.	Haney		MD	Department of Surgery, Duke University School of Medicine	Durham, North Carolina, USA		
David H.	Harpole	Jr.	MD	Division of Cardiovascular and Thoracic Surgery, Department of Surgery, Duke University School of Medicine	Durham, North Carolina, USA		
Bonita L.	Hilliard		RN, BSN	Department of Anesthesiology, Duke University School of Medicine	Durham, North Carolina, USA		
Julia	Hoang		BA	Department of Anesthesiology, Duke University School of Medicine	Durham, North Carolina, USA		
Jeffrey C.	Gadsden		MD	Department of Anesthesiology, Duke University School of Medicine	Durham, North Carolina, USA		
Mark J.	Gage		MD	Department of Orthopaedic Surgery, Duke University School of Medicine	Durham, North Carolina, USA		

Supplemental Online Content: Nonauthor Collaborators
*First name, last name, and suffix (if applicable) are required and will appear in PubMed.

***First Name and Middle Initial(s)**	***Last Name**	***Suffix (eg, Jr, III)**	Academic Degrees	Institution	Location (city, state/province, country)	Role or Contribution, eg, chair, principal investigator	Group (if more than 1 Group listed in the byline) and/or Subgroup (eg, Steering Committee)
Arun	Ganesh		MD	Department of Anesthesiology, Duke University School of Medicine	Durham, North Carolina, USA		
Jaleesa	Garner		MS	Department of Anesthesiology, Duke University School of Medicine	Durham, North Carolina, USA		
Jeffrey R.	Gingrich		MD	Department of Surgery, Duke University School of Medicine	Durham, North Carolina, USA		
Summer	Granger		MS	Department of Anesthesiology, Duke University School of Medicine	Durham, North Carolina, USA		
Rachel A.	Greenup		MD, MPH	Department of Surgery, Duke University School of Medicine	Durham, North Carolina, USA		
Christine	Ha		MS	Department of Anesthesiology, Duke University School of Medicine	Durham, North Carolina, USA		
Ashraf S.	Habib		MB BCh, MSc, MHSc	Department of Anesthesiology, Duke University School of Medicine	Durham, North Carolina, USA		
Ralph	Abi Hachem		MD, MSc	Department of Surgery, Duke University School of Medicine; Department of Head and Neck Surgery & Communication Sciences, Duke University School of Medicine	Durham, North Carolina, USA		
Ashley E.	Hall		BS	Department of Anesthesiology, Duke University School of Medicine	Durham, North Carolina, USA		
Matthew G.	Hartwig		MD	Department of Surgery, Duke University School of Medicine	Durham, North Carolina, USA		

Supplemental Online Content: Nonauthor Collaborators
*First name, last name, and suffix (if applicable) are required and will appear in PubMed.

***First Name and Middle Initial(s)**	***Last Name**	***Suffix (eg, Jr, III)**	Academic Degrees	Institution	Location (city, state/province, country)	Role or Contribution, eg, chair, principal investigator	Group (if more than 1 Group listed in the byline) and/or Subgroup (eg, Steering Committee)
Laura J.	Havrilesky		MD, MHSc	Department of Obstetrics and Gynecology, Duke University School of Medicine	Durham, North Carolina, USA		
Mitchell T.	Heflin		MD	Center for the Study of Aging and Human Development, Duke University School of Medicine; Department of Medicine, Duke University School of Medicine	Durham, North Carolina, USA		
Tarrah	Henley		BS	Department of Anesthesiology, Duke University School of Medicine	Durham, North Carolina, USA		
Scott T.	Hollenbeck		MD	Department of Surgery, Duke University School of Medicine	Durham, North Carolina, USA		
Thomas J.	Hopkins		MD	Department of Anesthesiology, Duke University School of Medicine	Durham, North Carolina, USA		
Brant A.	Inman		MD, MS	Department of Surgery, Duke University School of Medicine	Durham, North Carolina, USA		
David W.	Jang		MD	Department of Surgery, Duke University School of Medicine; Department of Neurosurgery, Duke University School of Medicine	Durham, North Carolina, USA		
Russel R.	Kahmke		MD MMCi	Department of Surgery, Duke University School of Medicine	Durham, North Carolina, USA		
Isaac O.	Karikari		MD	Department of Neurosurgery, Duke University School of Medicine,	Durham, North Carolina, USA		

Supplemental Online Content: Nonauthor Collaborators
*First name, last name, and suffix (if applicable) are required and will appear in PubMed.

***First Name and Middle Initial(s)**	***Last Name**	***Suffix (eg, Jr, III)**	Academic Degrees	Institution	Location (city, state/province, country)	Role or Contribution, eg, chair, principal investigator	Group (if more than 1 Group listed in the byline) and/or Subgroup (eg, Steering Committee)
Nicholas	Kathrein		MS	Department of Anesthesiology, Duke University School of Medicine	Durham, North Carolina, USA		
Amie	Kawasaki		MD	Department of Obstetrics and Gynecology, Duke University School of Medicine	Durham, North Carolina, USA		
Deborah R.	Kaye		MD	Department of Urology, Duke University School of Medicine	Durham, North Carolina, USA		
Hadiza S.	Kazaure		MD	Department of Surgery, Duke University School of Medicine	Durham, North Carolina, USA		
Josiane	Kerbage		MD	Department of Anesthesiology, Duke University School of Medicine	Durham, North Carolina, USA		
Jacob A.	Klapper		MD	Department of Surgery, Duke University School of Medicine	Durham, North Carolina, USA		
Christopher S.	Klifto		MD	Department of Orthopaedic Surgery, Duke University School of Medicine	Durham, North Carolina, USA		
Rebecca	Klinger		MD	Department of Anesthesiology, Duke University School of Medicine	Durham, North Carolina, USA		
Stuart J.	Knechtle		MD	Department of Surgery, Duke University School of Medicine	Durham, North Carolina, USA		
Sandhya A.	Lagoo-Deenadayalan		MD, PhD	Department of Surgery, Duke University School of Medicine	Durham, North Carolina, USA		
Billy Y.	Lan		MD	Department of Surgery, Duke University School of Medicine	Durham, North Carolina, USA		
Paula S.	Lee		MD, MPH	Department of Obstetrics and Gynecology, Duke University School of Medicine	Durham, North Carolina, USA		

***First Name and Middle Initial(s)**	***Last Name**	***Suffix (eg, Jr, III)**	Academic Degrees	Institution	Location (city, state/province, country)	Role or Contribution, eg, chair, principal investigator	Group (if more than 1 Group listed in the byline) and/or Subgroup (eg, Steering Committee)
Walter T.	Lee		MD, MHS	Department of Surgery, Duke University School of Medicine	Durham, North Carolina, USA		
Howard	Levinson		MD	Department of Surgery, Duke University School of Medicine; Department of Dermatology, Duke University School of Medicine; Department of Pathology, Duke University School of Medicine	Durham, North Carolina, USA		
Brian D.	Lewis		MD	Department of Anesthesiology, Duke University School of Medicine	Durham, North Carolina, USA		
Michael E.	Lidsky		MD	Department of Surgery, Duke University School of Medicine,	Durham, North Carolina, USA		
Janice	Lim		BS	Department of Anesthesiology, Duke University School of Medicine	Durham, North Carolina, USA		
Michael E.	Lipkin		MD	Department of Surgery, Duke University School of Medicine,	Durham, North Carolina, USA		
Andrew J.	Liu		MD, MS	Department of Neurology, Duke University School of Medicine; Department of Pathology, Duke University School of Medicine	Durham, North Carolina, USA		
Christopher R.	Mantyh		MD	Department of Surgery, Duke University School of Medicine,	Durham, North Carolina, USA		
Jeffrey R.	Marcus		MD	Department of Surgery, Duke University School of Medicine,	Durham, North Carolina, USA		
Hector F.	Martinez-Wilson		MD, PhD	Department of Anesthesiology, Duke University School of Medicine	Durham, North Carolina, USA		

Supplemental Online Content: Nonauthor Collaborators
*First name, last name, and suffix (if applicable) are required and will appear in PubMed.

***First Name and Middle Initial(s)**	***Last Name**	***Suffix (eg, Jr, III)**	Academic Degrees	Institution	Location (city, state/province, country)	Role or Contribution, eg, chair, principal investigator	Group (if more than 1 Group listed in the byline) and/or Subgroup (eg, Steering Committee)
Shelley R.	McDonald		DO	Center for the Study of Aging and Human Development, Duke University School of Medicine; Department of Medicine, Duke University School of Medicine	Durham, North Carolina, USA		
Jennifer L.	McNally		MD	Department of Obstetrics and Gynecology, Duke University School of Medicine	Durham, North Carolina, USA		
Nitin L.	Mehdiratta		MD	Department of Anesthesiology, Duke University School of Medicine	Durham, North Carolina, USA		
John	Migaly		MD	Department of Surgery, Duke University School of Medicine	Durham, North Carolina, USA		
Timothy E.	Miller		MB ChB	Department of Anesthesiology, Duke University School of Medicine	Durham, North Carolina, USA		
Suhail K.	Mithani		MD	Department of Surgery, Duke University School of Medicine	Durham, North Carolina, USA		
Sean P.	Montgomery		MD	Department of Surgery, Duke University School of Medicine	Durham, North Carolina, USA		
Paul J.	Mosca		MD, PhD, MBA	Department of Surgery, Duke University School of Medicine	Durham, North Carolina, USA		
Judd W.	Moul		MD	Department of Surgery, Duke University School of Medicine	Durham, North Carolina, USA		
David M.	Murdoch		MD, PhD	Department of Medicine, Duke University School of Medicine	Durham, North Carolina, USA		
Devika	Naphade		BS	Department of Anesthesiology, Duke University School of Medicine	Durham, North Carolina, USA		

Supplemental Online Content: Nonauthor Collaborators
*First name, last name, and suffix (if applicable) are required and will appear in PubMed.

***First Name and Middle Initial(s)**	***Last Name**	***Suffix (eg, Jr, III)**	Academic Degrees	Institution	Location (city, state/province, country)	Role or Contribution, eg, chair, principal investigator	Group (if more than 1 Group listed in the byline) and/or Subgroup (eg, Steering Committee)
Christine L.	Nelson		MSN, CRNA	Department of Anesthesiology, Duke University School of Medicine	Durham, North Carolina, USA		
Thomas L.	Novick		MD	Department of Surgery, Duke University School of Medicine	Durham, North Carolina, USA		
Daniel P.	Nussbaum		MD	Department of Surgery, Duke University School of Medicine	Durham, North Carolina, USA		
Brian J.	Ohlendorf		MD	Department of Anesthesiology, Duke University School of Medicine	Durham, North Carolina, USA		
Steven A.	Olson		MD	Department of Orthopaedic Surgery, Duke University School of Medicine	Durham, North Carolina, USA		
Isaac	Oyediran		MS	Department of Anesthesiology, Duke University School of Medicine	Durham, North Carolina, USA		
Theodore N.	Pappas		MD	Department of Surgery, Duke University School of Medicine	Durham, North Carolina, USA		
John J.	Park		MD	Department of Anesthesiology, Duke University School of Medicine	Durham, North Carolina, USA		
Ashley B.	Parker		MD, MS	Department of Anesthesiology, Duke University School of Medicine	Durham, North Carolina, USA		
Andrew C.	Peterson		MD, MPH	Department of Surgery, Duke University School of Medicine	Durham, North Carolina, USA		
Christy E.	Peterson		BS	Department of Anesthesiology, Duke University School of Medicine	Durham, North Carolina, USA		
Brett T.	Phillips		MD	Department of Surgery, Duke University School of Medicine	Durham, North Carolina, USA		

Supplemental Online Content: Nonauthor Collaborators
*First name, last name, and suffix (if applicable) are required and will appear in PubMed.

***First Name and Middle Initial(s)**	***Last Name**	***Suffix (eg, Jr, III)**	Academic Degrees	Institution	Location (city, state/province, country)	Role or Contribution, eg, chair, principal investigator	Group (if more than 1 Group listed in the byline) and/or Subgroup (eg, Steering Committee)
Thomas J.	Polascik		MD	Department of Surgery, Duke University School of Medicine	Durham, North Carolina, USA		
Peter T.	Potash		MD, MS	Department of Anesthesiology, Duke University School of Medicine	Durham, North Carolina, USA		
Glenn M.	Preminger		MD	Department of Surgery, Duke University School of Medicine	Durham, North Carolina, USA		
Rebecca A.	Previs		MD, MS	Department of Obstetrics and Gynecology, Duke University School of Medicine	Durham, North Carolina, USA		
Kadiyala V.	Ravindra		MD	Department of Surgery, Duke University School of Medicine	Durham, North Carolina, USA		
Sydney M.	Record		MD	Duke University School of Medicine	Durham, North Carolina, USA		
Aparna S.	Rege		MD	Department of Surgery, Duke University School of Medicine	Durham, North Carolina, USA		
Kristen M.	Rezak		MD	Department of Surgery, Duke University School of Medicine	Durham, North Carolina, USA		
Kenneth C.	Roberts		BS	Center for Cognitive Neuroscience, Duke University Medical Center	Durham, North Carolina, USA		
Cary N.	Robertson		MD	Department of Surgery, Duke University School of Medicine	Durham, North Carolina, USA		
Daniel J.	Rocke		MD	Department of Head and Neck Surgery & Communication Sciences, Duke University School of Medicine	Durham, North Carolina, USA		
David S.	Ruch		MD	Department of Orthopaedic Surgery, Duke University School of Medicine	Durham, North Carolina, USA		

Supplemental Online Content: Nonauthor Collaborators
*First name, last name, and suffix (if applicable) are required and will appear in PubMed.

***First Name and Middle Initial(s)**	***Last Name**	***Suffix (eg, Jr, III)**	Academic Degrees	Institution	Location (city, state/province, country)	Role or Contribution, eg, chair, principal investigator	Group (if more than 1 Group listed in the byline) and/or Subgroup (eg, Steering Committee)
Sean P.	Ryan		MD	Department of Orthopaedic Surgery, Duke University School of Medicine	Durham, North Carolina, USA		
Charles D.	Scales	Jr.	MD, MSHS	Department of Surgery, Duke University School of Medicine; Department of Population Health Sciences, Duke University,	Durham, North Carolina, USA		
Randall P.	Scheri		MD	Department of Surgery, Duke University School of Medicine,	Durham, North Carolina, USA		
Angeles A.	Secord		MD	Department of Obstetrics and Gynecology, Duke University School of Medicine	Durham, North Carolina, USA		
Keri A.	Seymour		DO	Department of Surgery, Duke University School of Medicine,	Durham, North Carolina, USA		
Ankeet M.	Shah		MD	Department of Urology, Duke University School of Medicine	Durham, North Carolina, USA		
Kevin N.	Shah		MD	Department of Surgery, Duke University School of Medicine	Durham, North Carolina, USA		
Nazema Y.	Siddiqui		MD, MHSc	Department of Obstetrics and Gynecology, Duke University School of Medicine,	Durham, North Carolina, USA		
Geoffroy C.	Sisk		MD	Department of Surgery, Duke University School of Medicine	Durham, North Carolina, USA		
Shayan	Smani		BS	Department of Anesthesiology, Duke University School of Medicine	Durham, North Carolina, USA		
Kevin W.	Southerland		MD	Department of Surgery, Duke University School of Medicine	Durham, North Carolina, USA		

Supplemental Online Content: Nonauthor Collaborators
*First name, last name, and suffix (if applicable) are required and will appear in PubMed.

***First Name and Middle Initial(s)**	***Last Name**	***Suffix (eg, Jr, III)**	Academic Degrees	Institution	Location (city, state/province, country)	Role or Contribution, eg, chair, principal investigator	Group (if more than 1 Group listed in the byline) and/or Subgroup (eg, Steering Committee)
Courtney H.	Stanczak		BS	Department of Anesthesiology, Duke University School of Medicine	Durham, North Carolina, USA		
Michael T.	Stang		MD	Department of Surgery, Duke University School of Medicine	Durham, North Carolina, USA		
Samuel D.	Stanley		MD	Department of Orthopaedic Surgery, Duke University School of Medicine	Durham, North Carolina, USA		
Niccolò	Terrando		PhD	Department of Anesthesiology, Duke University School of Medicine	Durham, North Carolina, USA		
Julie K. M.	Thacker		MD	Department of Surgery, Duke University School of Medicine	Durham, North Carolina, USA		
Brittany N.	Thomas		MS	Department of Anesthesiology, Duke University School of Medicine	Durham, North Carolina, USA		
Jake P.	Thomas		MD	Department of Anesthesiology, Duke University School of Medicine	Durham, North Carolina, USA		
Yanne	Toulgoat-DuBois		BA	Department of Anesthesiology, Duke University School of Medicine	Durham, North Carolina, USA		
Daniel C.	Villalobos		MD, MS	Duke University School of Medicine	Durham, North Carolina, USA		
Anthony G.	Visco		MD	Department of Obstetrics and Gynecology, Duke University School of Medicine,	Durham, North Carolina, USA		
Alison C.	Weidner		MD, MMCi	Department of Obstetrics and Gynecology, Duke University School of Medicine,	Durham, North Carolina, USA		

Supplemental Online Content: Nonauthor Collaborators
*First name, last name, and suffix (if applicable) are required and will appear in PubMed.

***First Name and Middle Initial(s)**	***Last Name**	***Suffix (eg, Jr, III)**	Academic Degrees	Institution	Location (city, state/province, country)	Role or Contribution, eg, chair, principal investigator	Group (if more than 1 Group listed in the byline) and/or Subgroup (eg, Steering Committee)
John	Whittle		MD, MBBS	Centre for Perioperative Medicine, University College London	London, UK		
David A.	Williams		MD	Department of Anesthesiology, Duke University School of Medicine	Durham, North Carolina, USA		
Megan	Wong		MD	Duke University School of Medicine	Durham, North Carolina, USA		
Mamata	Yanamadala		MBBS	Center for the Study of Aging and Human Development, Duke University School of Medicine; Department of Medicine, Duke University School of Medicine	Durham, North Carolina, USA		
Rosa O.	Yang		MD	Duke University School of Medicine	Durham, North Carolina, USA		
Christopher C.	Young		MD	Department of Anesthesiology, Duke University School of Medicine	Durham, North Carolina, USA		
Mary	Yurashevich		MD, MPH	Department of Anesthesiology, Duke University School of Medicine	Durham, North Carolina, USA		
Sabino	Zani	Jr.	MD	Department of Surgery, Duke University School of Medicine; Department of Mechanical Engineering and Materials Science, Duke University	Durham, North Carolina, USA		
Daphne	Zhu		BS	Duke University School of Medicine	Durham, North Carolina, USA		
