# Supplementary material for: Apolipoprotein E Mimetic Peptide CN-105 and Postoperative Delirium in Older Patients: The Phase 2 MARBLE Randomized Clinical Trial
Source: JAMA Netw Open. 2026 Apr 3;9(4):e262289. doi: 10.1001/jamanetworkopen.2026.2289 (PMC13049496; doi:10.1001/jamanetworkopen.2026.2289)
Supplement: Supplement 4. — Data Sharing Statement [file jamanetwopen-e262289-s004.pdf]

Data Sharing Statement

Timko. Apolipoprotein E Mimetic Peptide CN-105 and Postoperative Delirium in Older Patients. *JAMA Netw Open*. Published April 03, 2026. doi:10.1001/jamanetworkopen.2026.2289

Data
Additional Information: Name of the trial registry: ClinicalTrials.gov URL: https://clinicaltrials.gov/study/NCT03802396?term=NCT03802396%20&rank=1
ClinicalTrials.gov Identifier: NCT03802396
Data available: Yes
Data types: Deidentified participant data, Data dictionary
How to access data: How to access data: https://clinicaltrials.gov/; Submission of a data request. Please email Dr. Bethany J. Hsia (bethany.brown@duke.edu) for more information.
When available: With publication

Supporting Documents
Document types: None

Additional Information
Who can access the data: Who can access the data: Only researchers whose proposed use of the data has been approved.
Types of analyses: Types of analyses: Only for pre-approved specific purposes.
Mechanisms of data availability: Mechanisms of data availability: Access to the primary data is available from the study principle investigator after completion of the necessary legal agreements. Please email Dr. Bethany J. Hsia (bethany.brown@duke.edu) for more information.
